# Genomic evidence supports the merging of *Camellia* sect. *Eriandria* and sect. *Theopsis*: ovary pubescence as a trait of potential ecological relevance

**DOI:** 10.3389/fpls.2026.1932891

**Published:** 2026-09-24

**Authors:** Mengwen Lyu, Min Chen, Mengwei Fang, Yin Wu, Bingling Li, Yali Zhang

**Affiliations:** 1 Forest and Fruit Tree Research Institute, Shanghai Academy of Agricultural Sciences, Shanghai, China; 2 School of Landscape Architecture, Beijing Forestry University, Beijing, China

**Keywords:** *Camellia*, ovary pubescence, population genomics, sect. *Eriandria*, sect. *Theopsis*

## Abstract

**Introduction:**

*Camellia* sect. *Eriandria* and sect. *Theopsis*, mostly endemic to China, possess a suite of desirable traits, including fragrance, floriferous flowering, and stress tolerance, which have drawn increasing research interest. However, research remains limited, and their classification has long been controversial as it relies solely on the morphological trait of ovary pubescence (OP), leading to numerous synonyms and homonyms that severely hinder germplasm conservation and utilization.

**Methods:**

In this study, we performed super genotyping-by-sequencing (super-GBS) on 87 wild accessions representing both sections, together with five outgroup accessions, generating genome-wide single nucleotide polymorphism (SNP) data. This foundational genomic resource enabled subsequent population genetic structure analyses, phylogenomic reconstruction, genome-wide selection scans (FST and XP-CLR), and genome-wide association studies (GWAS) to systematically investigate their genetic structure and evolutionary mechanisms.

**Results and discussion:**

Population structure and phylogenetic analyses revealed extensive admixture with no distinct genetic lineages between the two sections. The genome-wide mean FST was 0.013 (95% CI: 0.0125–0.0129), and standardized differentiation indices (G′ST = 0.016, Jost’s D = 0.005) confirmed the absence of a significant genetic barrier. By integrating FST, XP-CLR, and GWAS, we identified 208 candidate genes from OP-stratified genome-wide scans. However, due to the limited marker density and the absence of genome-wide significant GWAS signals, these candidate genes should be regarded as exploratory. Collectively, our genomic evidence supports the merging of *Camellia* sect. *Eriandria* and sect. *Theopsis* into a single, cohesive genetic group, and suggests that OP is a trait of potential ecological relevance rather than a reliable diagnostic character for sectional delimitation. These findings provide genome-wide evidence for taxonomic revision and a molecular foundation for germplasm conservation and breeding in *Camellia*.

## 1 Introduction

Within the family Theaceae, *Camellia* Linn. ranks among the largest genera and occupies a rather basal evolutionary position. It has traditionally attracted considerable research interest due to its immense economic and horticultural significance, encompassing woody oil production, tea cultivation, and ornamental application. Recent studies have predominantly focused on *Camellia* sect. *Thea*, sect. *Oleifera*, sect. *Camellia*, and sect. *Chrysantha* (Jin et al., 2022; Li et al., 2023, 2024; Zhang et al., 2024; Kong et al., 2025; Yang et al., 2025), while other sections remain markedly understudied, particularly sect. *Eriandria* and sect. *Theopsis*. These two sections, largely endemic to China, are traditionally distinguished by a single morphological character: the presence or absence of pubescence on the ovary (Chang, 1981). Both sections are evergreen small trees or shrubs with fragrant flowers and notable stress tolerance (Song et al., 2009; Guo et al., 2014; Zhang et al., 2021). Beyond their direct applications in beverage consumption and essential oil extraction, they are crucial germplasm resources for breeding tea flower cultivars with abundant flowering and aromatic characteristics (Dong et al., 2024). Thus, they offer substantial potential for further resource development and utilization.

To date, three major taxonomic systems have been proposed for the genus *Camellia* Linn.: the Sealy system (Sealy, 1958), the Chang system (Chang, 1981; Chang and Bartholomew, 1984; Chang and Ren, 1991, 1992), and the Ming system (Ming, 1999, 2000). All three systems are primarily constructed based on morphological characters, which are subject to subjective interpretation and environmental influences such as topography and climate. Consequently, substantial discrepancies exist among these systems regarding the delimitation of subgenera, sections, and species. The taxonomic status and species boundaries of sect. *Eriandria* and sect. *Theopsis* are particularly contentious (**Table 1**). This taxonomic uncertainty has not only resulted in nomenclatural confusion and conflicting research conclusions but has also severely hindered the systematic exploration and sustainable utilization of these two sections.

**Table 1:** Species counts for *Camellia* sect. *Eriandria* and sect. *Theopsis* across three major taxonomic classification systems.

Classification systems	Subgenus placement	Species count (Sect. *Eriandria*)	Species count (Sect. *Theopsis*)	References
Sealy System	–	6	29	Sealy (1958)
Chang System	*Camellia* subgen *Metacamellia* Hung T. Chang	14	49	Chang (1981); Chang and Bartholomew (1984); Chang and Ren (1991, 1992)
Ming System	*Camellia s*ubgen. *Thea* Chang Tax. Gen	9	19	Ming (1999, 2000)

The species count includes species and subspecies.

In recent years, the rapid development of molecular systematics and phylogenomics has not only deepened our understanding of the evolutionary patterns within *Camellia* but also prompted a critical reassessment of traditional taxonomic systems that rely on a limited suite of morphological traits. Ye (1993) pointed out that sect. *Eriandria* and sect. *Theopsis* differ exclusively in the presence or absence of ovary pubescence (OP) and should not be treated as two independent sections. Subsequent phylogenetic studies based on molecular markers have provided support for this view. Using ITS sequences, Xiao and Parks (2002) and Vijayan et al. (2009) showed that the two sections cluster together in phylogenetic trees. Lyu (2017), using chloroplast DNA fragments, further demonstrated that they form a natural monophyletic group. Hong (2011) and Jiang (2017) observed that some species of sect. *Eriandria* are more closely related to species of sect. *Theopsis*.

Hu and Liu (2024), based on chloroplast genome phylogenetic analysis, corroborated a close relationship between *C. cordifolia* and *C. cuspidata*. More recently, phylogenomic studies based on large-scale nuclear gene data have further clarified their relationship. Zan et al. (2023) reconstructed the phylogenetic framework of *Camellia* using 1,617 orthologous low-copy nuclear genes and found that species of sect. *Eriandria* and sect. *Theopsis* together form a well-supported monophyletic clade, with species reciprocally nested within it—a pattern that does not support their recognition as two independent sections as defined by traditional morphology. Zhang et al. (2023), based on analysis of 60 transcriptomes, also confirmed the close relationship between the two sections. Although the above studies consistently support the merger of the two sections across various molecular marker perspectives, most of them are limited to a few chloroplast or nuclear gene fragments. Consequently, key questions regarding their population genetic structure, signatures of natural selection, and the genetic basis underlying the diagnostic morphological trait (OP) remain largely unaddressed at the genome-wide scale.

To systematically resolve the population genetic structure, evolutionary history, and taxonomic boundaries between *Camellia* sect. *Eriandria* and sect. *Theopsis*, we sampled 87 wild accessions representing both sections, together with five outgroup accessions from other sections of *Camellia*, and generated genome-wide single nucleotide polymorphism (SNP) markers through super genotyping-by-sequencing (super-GBS). Based on this dataset, we performed integrative population genomic analyses: principal component analysis (PCA), maximum-likelihood phylogenetic reconstruction, and ADMIXTURE-based ancestry inference were applied to reveal extensive genetic admixture and shared ancestral components between the two sections; fixation index (FST) and standardized differentiation indices (G′ST and Jost’s D) were calculated to quantify the level of genetic differentiation; and genome-wide scans based on all 92 accessions grouped by OP phenotype, including FST, cross-population composite likelihood ratio (XP-CLR), and genome-wide association studies (GWAS), were employed to identify selection signatures and candidate genes associated with OP variation. In addition, Mantel tests were performed to evaluate the effects of geographic distance and elevation on genetic differentiation, and sensitivity analyses based on 51 taxonomically confirmed accessions were conducted to validate the robustness of the main conclusions. This study systematically elucidates the phylogenetic affinity between sect. *Eriandria* and sect. *Theopsis*, provides genomic evidence for their taxonomic revision, and establishes a theoretical foundation for the scientific conservation and molecular breeding of these valuable germplasm resources.

## 2 Materials and methods

### 2.1 Plant materials

A total of 92 accessions of *Camellia* were included in this study, comprising 87 focal accessions, including 26 accessions of sect. *Eriandria* and 61 accessions of sect. *Theopsis*, together with five outgroup accessions from other sections. The 87 focal accessions were collected from 11 provinces in China between 2017 and 2024 (**Figure 1**). Among them, 58 accessions were sampled from native habitats with precise latitude, longitude, and elevation, under necessary permits and in accordance with national and institutional regulations (marked as “Yes” for “Collected from native habitat” in **Supplementary Table S1**), while 29 accessions were obtained from *ex situ* conservation sites (marked as “No”). All plant materials are preserved in the common garden of the Shanghai Academy of Agricultural Sciences for long-term phenotypic observation. Species identification was rigorously performed based on consultation of type (holotype) specimen, protologue and original descriptions, and further confirmed by taxonomic experts. However, due to the absence of type specimens or insufficient original descriptions, 27 accessions in sect. *Theopsis* and nine accessions in sect. *Eriandria* remained as *species inquirendae*. These 36 accessions of uncertain status were included in our study to assess their potential taxonomic status or impacts on the main findings. In addition, five outgroup accessions from other sections of *Camellia* were included to provide a phylogenetic root and to allow trait association analyses independent of sectional assignment. Detailed information for all accessions is provided in **Supplementary Table S1**.

**Figure 1:**
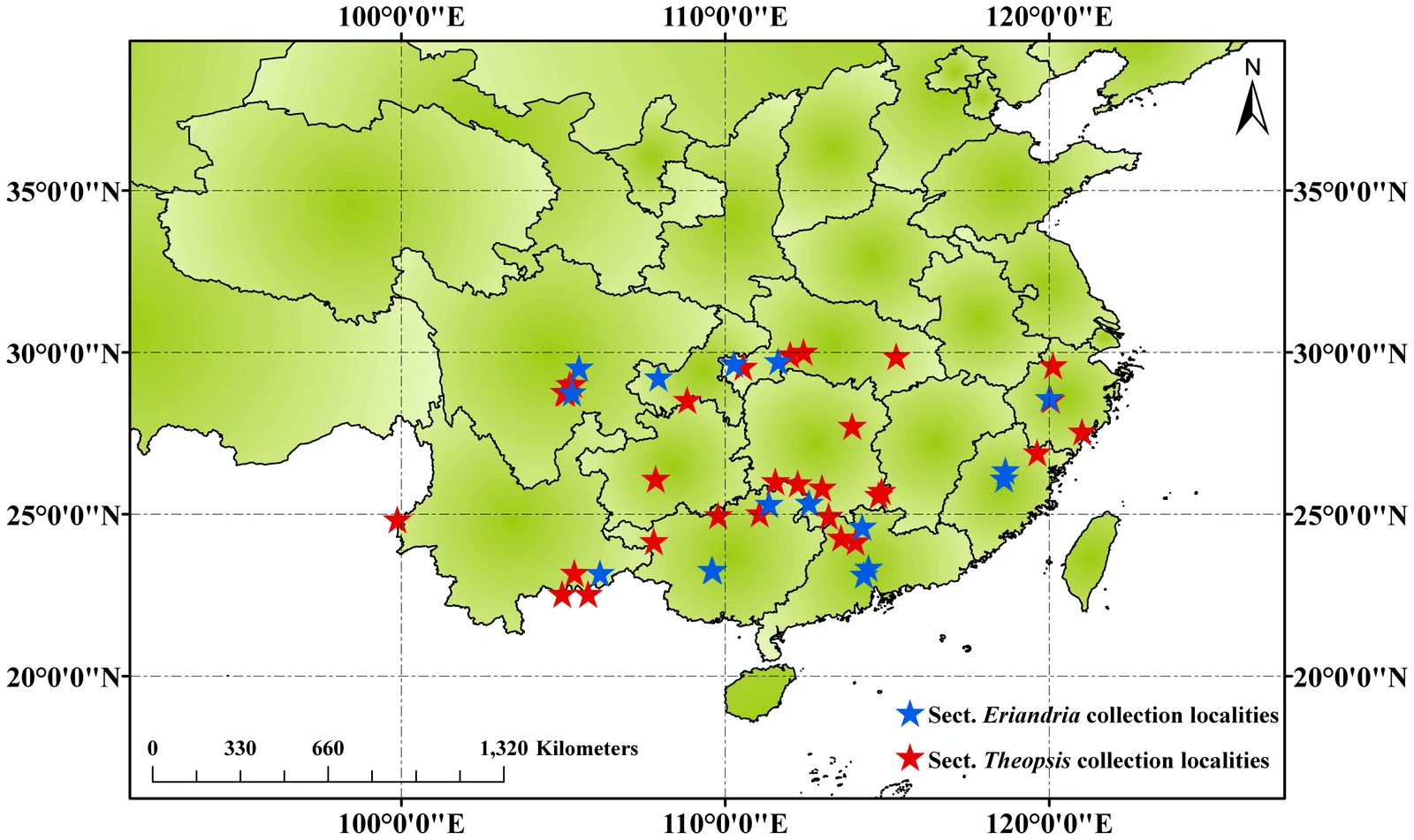
Wild germplasm sampling sites for *Camellia* sect. *Eriandria* and sect. *Theopsis.* The National Geomatics Center of China provided the standard map (GS (2024) 0650) from which this figure was created. The base map was not altered.

### 2.2 Morphological determination

The main morphological characters of all 92 accessions were observed and documented from 2021 to 2025. OP was scored as a binary trait: 1 for pubescent ovaries and 0 for glabrous ovaries (**Supplementary Table S1**). This scoring system was applied consistently to both the focal accessions and the outgroup accessions.

### 2.3 Super-GBS sequencing, SNP calling, and data quality control

#### 2.3.1 Library construction and sequencing

Young leaves were collected from all 92 accessions. High-quality genomic DNA was extracted, and super-GBS libraries were constructed, including restriction digestion, barcode adapter ligation, fragment size selection (300–700 bp), and PCR amplification. Libraries were sequenced on the Illumina Nova platform (Illumina, San Diego, CA, USA) with paired-end 150 bp reads.

#### 2.3.2 Read filtering and SNP calling

Raw reads were demultiplexed by barcode using Stacks and quality-filtered with fastp to obtain clean reads. Clean reads were then aligned to the *Camellia sinensis* reference genome (GCA_013676235.1_HZAU_G240_1.0_genomic) using Burrows–Wheeler Aligner (BWA). SNP calling was performed using GATK following standard best practices. The mean mapping rates for sect. *Eriandria*, sect. *Theopsis*, and outgroup accessions were 93.25%, 96.07%, and 96.96%, respectively (**Supplementary Table S2**), indicating comparable mapping success across categories and no substantial reference-mapping bias.

#### 2.3.3 SNP filtering

The raw VCF file was filtered using PLINK 2.0 with the following criteria: (1) only biallelic SNPs were retained (--max-alleles 2); (2) loci with genotype missing rate ≥20% were removed (--geno 0.2); (3) SNPs with minor allele frequency (MAF) <0.05 were excluded (--maf 0.05). Additionally, only SNPs located on the 15 main chromosomes (designated with a “CM” prefix in the reference genome) were retained for downstream analyses; SNPs on unanchored scaffolds were excluded due to the lack of chromosomal position information. After filtering, 35,885 high-quality SNPs were retained.

#### 2.3.4 Sample verification

To identify potential clonal or duplicate accessions, pairwise identity-by-state (IBS) distances were calculated from the filtered SNP dataset using PLINK 2.0. No sample pairs with IBS distance <0.05 were identified, indicating the absence of clonal duplicates among the 92 accessions (**Supplementary Table S4**). Per-sample missing rates and observed heterozygosity were also calculated using PLINK 2.0 to assess data quality. To test whether the per-sample divergence index was confounded by data quality, Spearman rank correlations were calculated between the divergence index and both missing rate and observed heterozygosity, based solely on the 87 focal accessions. All statistical tests were performed using Python 3.9 with SciPy.

### 2.4 Genome-wide SNP characterization and linkage disequilibrium

#### 2.4.1 SNP density and functional annotation

Genome-wide SNP density was calculated using 1 Mb sliding windows along the 15 main chromosomes and visualized as a heatmap. Functional annotation of SNPs was performed by overlapping SNP coordinates with reference genome annotations, categorizing SNPs into exonic, intronic, intergenic, upstream, downstream, and splicing regions. Transition (A ↔ G, C ↔ T) and transversion frequencies, as well as the transition (Ts)/transversion (Tv) ratio, were calculated from REF/ALT substitution patterns. All computations and visualizations were performed using Python 3.9.

#### 2.4.2 LD decay and gene-assignment window

Linkage disequilibrium (LD) decay was estimated using the 35,885 high-quality SNPs from the 87 focal accessions. Pairwise r² values were calculated for SNP pairs within 500 kb and averaged over 1 kb bins. An exponential decay model (r² = a × exp (−b × d)) was fitted to the data. Given the extremely rapid LD decay and the average marker spacing of approximately 84 kb, a conservative 50 kb physical window was adopted for SNP-to-gene assignment. Candidate genes identified within these windows should be regarded as exploratory and require functional validation.

### 2.5 Population genetic structure and genetic differentiation

#### 2.5.1 PCA and LD pruning

To avoid overestimation of population structure due to linkage disequilibrium (LD), LD pruning was performed prior to PCA using PLINK 2.0 with the --indep-pairwise 50 10 0.2 parameter (window size of 50 SNPs, step size of 10 SNPs, and r² threshold of 0.2). PCA was then conducted on the pruned SNP set using the --pca 10 option in PLINK 2.0. The first three principal components (PC1–PC3) were calculated, and the proportion of variance explained by each component was determined. PCA plots were generated using Python with matplotlib and seaborn, with samples colored by group (sect. *Eriandria* vs. sect. *Theopsis*) and outgroup accessions marked with distinct symbols.

#### 2.5.2 ADMIXTURE analysis

Population structure was inferred using ADMIXTURE based on the 87 focal accessions. The number of ancestral populations (K) was tested from 2 to 10, and the optimal K was determined by cross-validation (CV) error. Ancestry proportions were visualized using stacked bar plots.

#### 2.5.3 Phylogenetic analysis

Phylogenetic analysis was performed using IQ-TREE version 3.1.3 based on the filtered SNP dataset for 92 accessions. The best-fit nucleotide substitution model was selected by ModelFinder based on the Bayesian information criterion (BIC). The selected model was TVM + F + G4. Nodal support was assessed using 1,000 ultrafast bootstrap replicates. The tree was rooted using the five outgroup accessions.

#### 2.5.4 Pairwise genetic distance

Pairwise phylogenetic distances among the 92 accessions were extracted from the rooted maximum-likelihood tree as patristic distances using the cophenetic function in the R package ape. These distances were used to quantify genetic relatedness and were visualized as a clustered heatmap (**Figure 6**; **Supplementary Table S5**).

#### 2.5.5 FST and genetic diversity between focal sections

Weir and Cockerham’s FST between sect. *Eriandria* and sect. *Theopsis* was estimated using a custom Python script based on genotype data from the 87 focal accessions (Weir and Cockerham, 1984). For each SNP, within-population heterozygosity (HS) and total heterozygosity (HT) were calculated. Standardized G′ST and Jost’s D were also computed to provide differentiation metrics less dependent on within-population diversity. Bootstrap resampling with 1,000 replicates was performed to obtain 95% confidence intervals for mean FST.

#### 2.5.6 Isolation by distance

To evaluate the effects of geographic distance and elevation on genetic differentiation, Mantel tests were performed separately. The genetic distance matrix was computed as 1 − IBS from the filtered SNP dataset. Geographic distances (km) were calculated from latitude and longitude using the Haversine formula, and elevational distances were expressed as absolute differences in elevation between samples. Mantel tests were conducted with 999 permutations to assess significance. All analyses were performed using Python 3.9 (numpy, scipy, pandas, matplotlib).

### 2.6 Genome-wide scans for loci associated with OP

To identify genomic regions putatively associated with OP, all 92 accessions were grouped by OP phenotype (pubescent vs. glabrous) regardless of sectional assignment. Three complementary genome-wide scans (FST, XP-CLR, and GWAS) were performed based on this grouping.

#### 2.6.1 FST and XP-CLR scans by OP phenotype

To identify genomic regions associated with OP, all 92 accessions were grouped by OP phenotype into pubescent and glabrous groups. Genome-wide scans were then performed using the same sliding window size of 1 Mb and step size of 100 kb. First, Weir and Cockerham’s FST between the two phenotypic groups was calculated using VCFtools, and the top 1% of windows with the highest FST values were considered candidate regions potentially associated with OP. Second, XP-CLR was run using the pubescent and glabrous groups as contrasting populations to detect selective sweep signals.

#### 2.6.2 GWAS for OP

GWAS analysis was performed using GCTA version 1.94.1 with a mixed linear model. The binary OP phenotype (pubescent = 1, glabrous = 0) was used as the response variable. The first three principal components were included as fixed covariates to account for population structure, and the genetic relationship matrix was fitted as a random effect. A total of 35,885 high-quality SNPs (MAF ≥0.05, missing rate ≤20%, main chromosomes only) were included in the analysis. Genome-wide significance was determined using Bonferroni correction (P <1.39 × 10^−6^), and a suggestive threshold of P <1 × 10^−^4^^ was also considered. The genomic inflation factor (λ) was calculated to assess model fit. Manhattan plots and Q–Q plots were generated with Python.

### 2.7 Functional annotation of candidate genes

Candidate genes identified from the FST, XP-CLR, and GWAS scans were annotated using Gene Ontology (GO) and Kyoto Encyclopedia of Genes and Genomes (KEGG) databases. Annotation results were summarized and visualized as descriptive functional categories. Functional validation of these candidate genes requires further experimental investigation.

### 2.8 Sensitivity analysis using taxonomically confirmed accessions

To assess whether the inclusion of taxonomically uncertain accessions biased the main conclusions, key analyses were repeated using only the 51 accessions with confirmed species identification from sect. *Eriandria* and sect. *Theopsis*. PCA, ADMIXTURE, and mean FST between the two sections were recalculated based on this subset and compared with the results from the full 87-accession dataset.

## 3 Results

### 3.1 Morphological distinction and geographic distribution patterns

Sect. *Eriandria* and sect. *Theopsis* are similar in sepal, bract, and flower insertion traits, but differ consistently in OP: the former has densely pubescent ovaries, whereas the latter has glabrous ovaries (**Figures 2A, B**). This trait remained stable across five consecutive years of observation. In addition, the five outgroup accessions also varied in OP (two pubescent, three glabrous), providing an independent set for trait association analyses (**Supplementary Table S1**).

**Figure 2:**
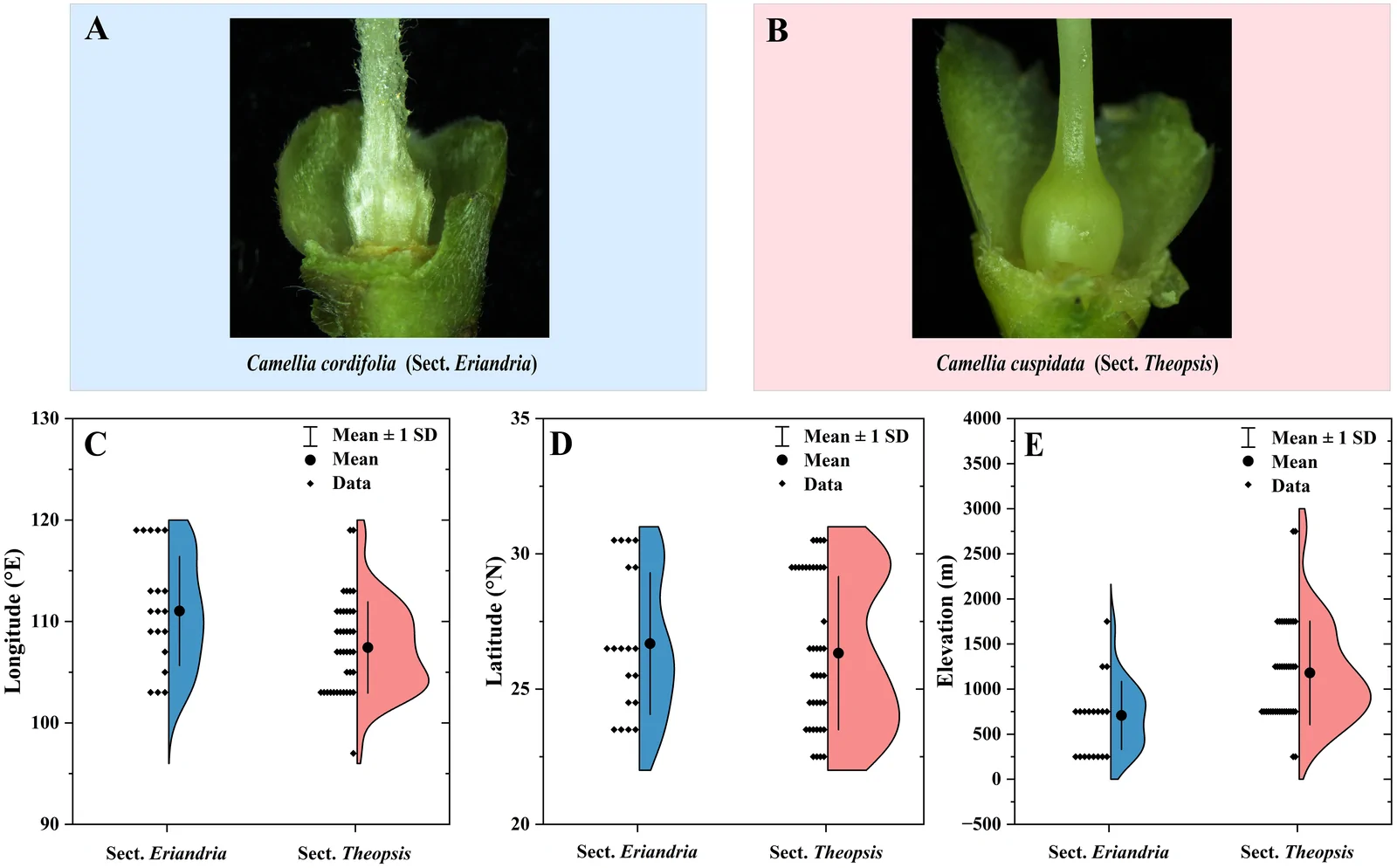
Key taxonomic traits and geographical distribution of *Camellia* sect. *Eriandria* and sect. *Theopsis.* **(A)** *C*. *cordifolia* (sect. *Eriandria*) with a pubescent ovary. **(B)** *C*. *cuspidata* (sect. *Theopsis*) with a glabrous ovary. **(C–E)** Native geographical distribution of *Camellia* sect. *Eriandria* and sect. *Theopsis* in terms of longitude, latitude, and elevation.

To characterize the ecological and biogeographic attributes of these two sections at a macroecological scale, we compiled distribution data for 58 wild accessions with confirmed geographic origins, comprising 19 accessions of sect. *Eriandria* and 39 accessions of sect. *Theopsis* (**Supplementary Table S1**). The sampled accessions span latitudes from 22.51°N (Mengzi, Yunnan Province) to 30.61°N (Changyang, Hubei Province), longitudes from 97.56°E (Yingjiang, Yunnan Province) to 119.19°E (Lishui, Zhejiang Province), and elevations ranging from 199 m to 2,855 m. Sect. *Eriandria* is predominantly distributed approximately 26°N and 118°–119°E. By contrast, sect. *Theopsis* is mainly concentrated near 29°N, with a broader longitudinal range (mostly 103°–114°E) and a more uniform distribution across 22°–26°N. In terms of elevational distribution, sect. *Theopsis* occupies a wider range, predominantly between 700 m and 2,000 m, which is higher than the typical range of sect. *Eriandria* (mostly 260–850 m) (**Figures 2C–E**). These contrasting distribution patterns are consistent with different ecological preferences between the two sections, although formal environmental association testing is required to evaluate this hypothesis.

### 3.2 Genome-wide SNP characterization and data quality control

After stringent quality filtering, a total of 35,885 high-quality SNPs were obtained from 92 accessions. The SNP density heatmap showed a relatively uniform distribution of SNPs across the 15 main chromosomes (**Figure 3A**). Ts accounted for 71.5% (25,669) of all SNPs, while Tv accounted for 28.5% (10,216), yielding a Ts/Tv ratio of 2.51 (**Figure 3B**, **Supplementary Table S3**). Functional annotation indicated that 34.3% of SNPs were located in exonic regions, 34.0% in intronic regions, 19.4% in intergenic regions, 8.2% in downstream regulatory regions, 4.0% in upstream regulatory regions, and only 0.1% at splice sites (**Figure 3C**, **Supplementary Table S3**). The observed Ts/Tv ratio is consistent with the transition bias commonly observed in plant and animal genomes. This pattern may be attributed to multiple factors, including long-term natural selection, mutational preferences, and functional constraints. Specifically, transitions in coding regions are more likely to result in synonymous substitutions, which are more readily retained during evolution. Additionally, DNA replication and repair mechanisms, as well as the structural properties of the DNA double helix, may consistently bias the Ts/Tv ratio in favor of transitions (Li et al., 1984; Collins and Jukes, 1994; Wakeley, 1996; Tang et al., 2008; Fan et al., 2018). These results provide insights into the patterns of selection pressure and mutation accumulation throughout the evolutionary history of the two sections.

**Figure 3:**
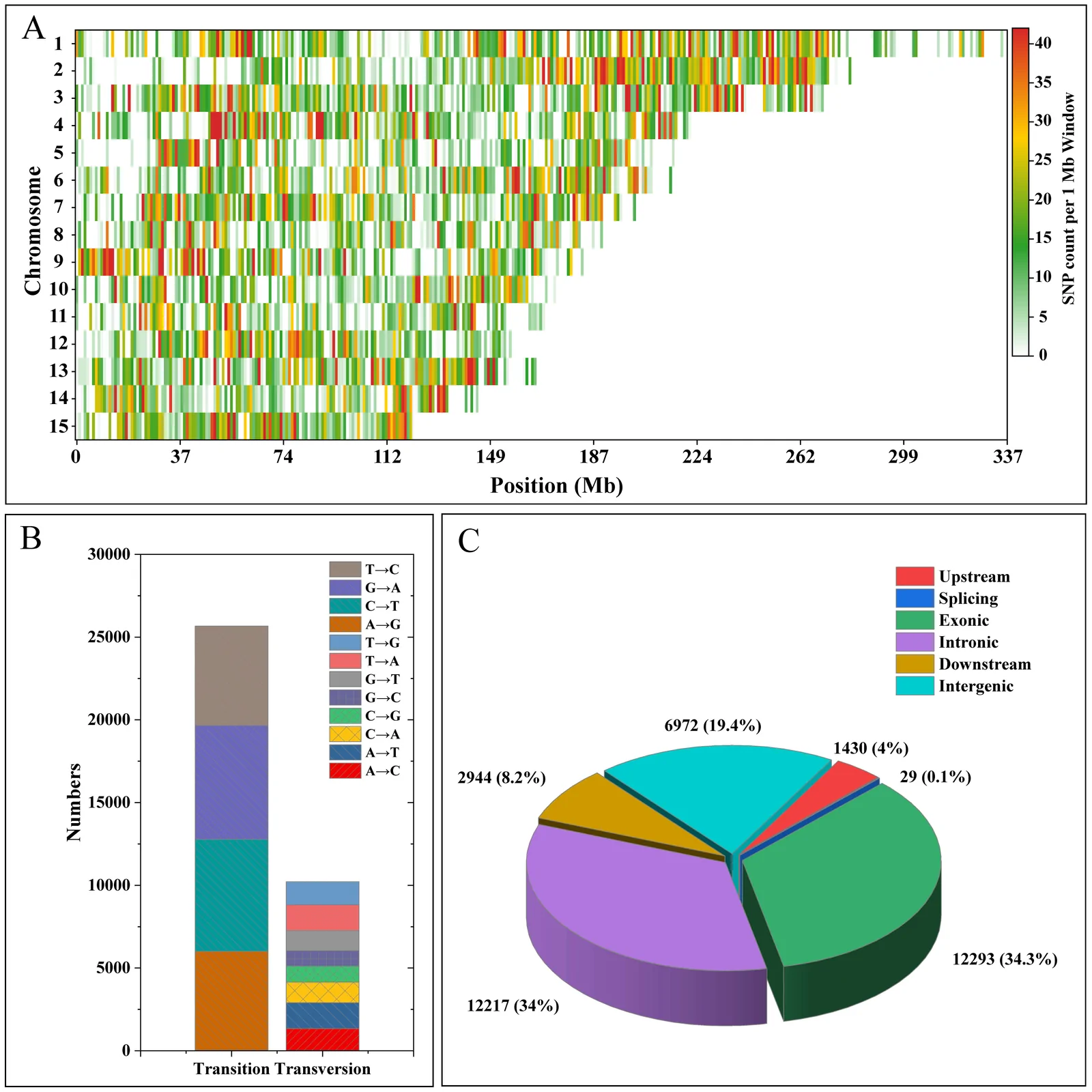
Genome-wide distribution and characteristics of SNPs identified in all 92 accessions of *Camellia*, including sect. *Eriandria*, sect. *Theopsis*, and five outgroup accessions from other sections. **(A)** Genome-wide SNP density distribution across chromosomes. **(B)** Proportion of transition and transversion substitutions. **(C)** Distribution of SNPs across different genomic features.

Clone detection identified no sample pairs with IBS distance <0.05 among the 92 accessions (**Supplementary Table S4**), indicating that all samples were genetically independent individuals. For the 87 focal accessions, no significant correlations were detected between the divergence index and missing rate (Spearman ρ = −0.072, P = 0.508) or observed heterozygosity (ρ = 0.080, P = 0.459) (**Supplementary Figures S1A, B**), confirming that the divergence index was not biased by missing-data artifacts.

Genome-wide LD decay analysis based on the 87 focal accessions showed that mean r² declined rapidly with physical distance, falling below 0.1 within 1 kb (**Supplementary Figure S1C**), indicating extremely rapid LD decay consistent with an outcrossing perennial species. Because of the low initial r² values, the LD decay distance could not be estimated at the r² = 0.2 threshold; at the r² = 0.1 threshold, the decay distance was less than 1 kb. Accordingly, a conservative 50 kb physical window was adopted for SNP-to-gene assignment.

### 3.3 Population genetic structure

PCA revealed that the first three principal components explained 19.4%, 15.2%, and 13.8% of the total genetic variance, respectively. Outgroup accessions were clearly separated from the 87 focal accessions along PC1, whereas samples of sect. *Eriandria* and sect. *Theopsis* were extensively intermixed in PC space, showing no distinct genetic clustering (**Figure 4A**). Cross-validation error of ADMIXTURE for the 87 focal accessions reached a minimum at K = 4 (CV error = 0.5261) (**Figure 4B**). At K = 4, all focal accessions exhibited admixed ancestry components, with no single ancestral component capable of distinguishing sect. *Eriandria* from sect. *Theopsis* (**Figure 4C**).

**Figure 4:**
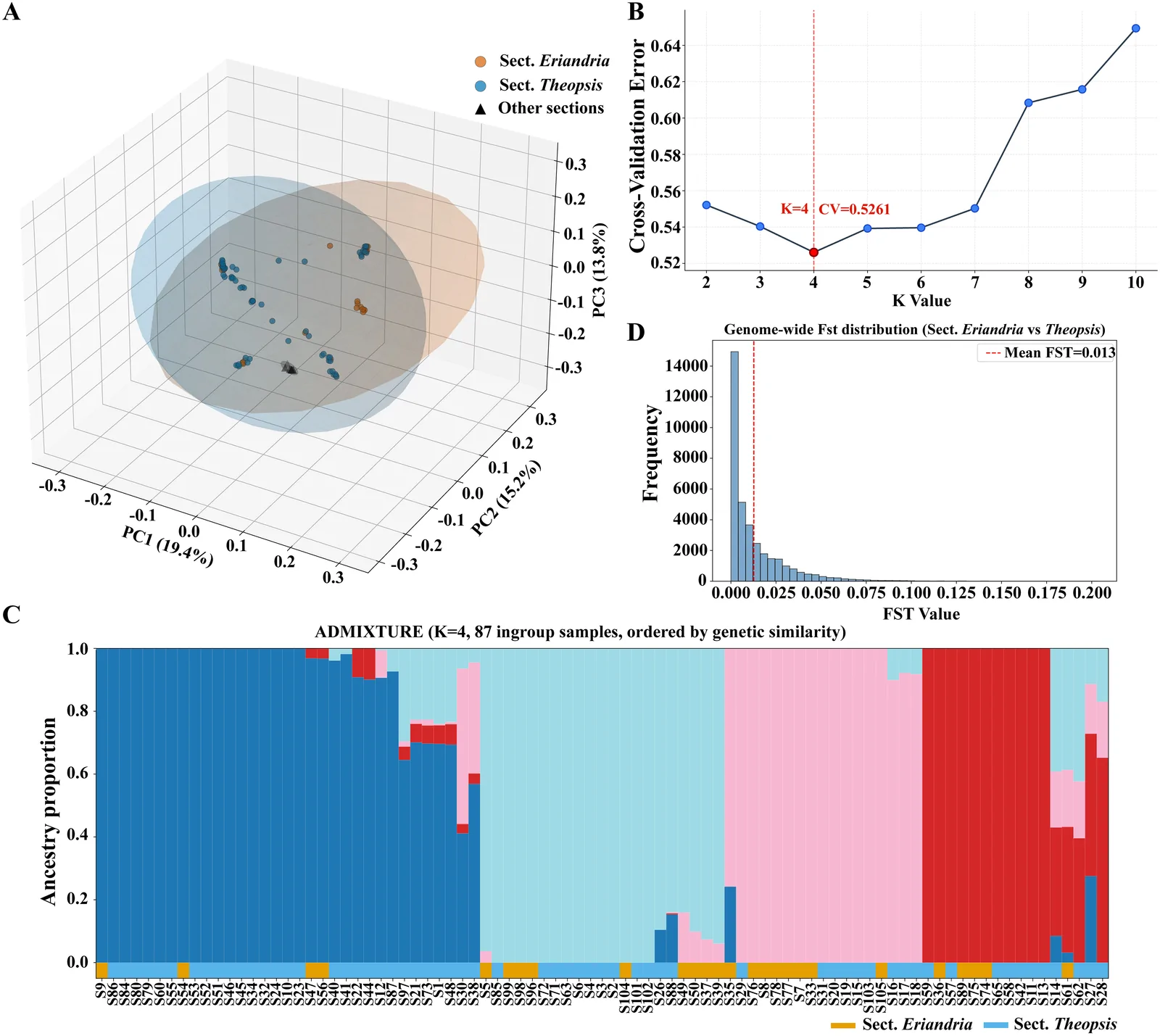
Population genetic structure and genome-wide differentiation of *Camellia* sect. *Eriandria* and sect. *Theopsis*. **(A)** Three-dimensional principal component analysis (PCA) of 92 accessions, comprising 87 accessions from the two focal sections and five outgroup accessions from other sections of *Camellia*; ellipses denote 95% confidence regions. **(B)** Cross-validation error as a function of K, estimated from the 87 focal accessions, used to determine the optimal number of ancestral populations. **(C)** Model-based clustering of the 87 focal accessions at K = 4, inferred using ADMIXTURE. **(D)** Genome-wide FST between sect. *Eriandria* and sect. *Theopsis*, calculated from the 87 focal accessions.

The genome-wide mean FST between the two focal sections, estimated from the 87 focal accessions, was 0.013 (95% bootstrap confidence interval: 0.0125–0.0129), indicating extremely low genetic differentiation. Within-population expected heterozygosity (HS = 0.230) was nearly equal to total expected heterozygosity (HT = 0.234), demonstrating that the vast majority of genetic variation resided within rather than between sections. Standardized differentiation indices G′ST = 0.016 and Jost’s D = 0.005 further confirmed the absence of a significant genetic barrier between the two sections (**Figure 4D**, **Table 2**).

**Table 2:** Genetic differentiation statistics between sect. *Eriandria* and sect. *Theopsis*.

Statistic	Value
Genome-wide mean FST	0.013
95% bootstrap confidence interval of FST	0.0125–0.0129
Within-population expected heterozygosity (HS)	0.230
Total expected heterozygosity (HT)	0.234
Standardized differentiation index (G′ST)	0.016
Jost’s D	0.005

To evaluate the potential impact of taxonomically uncertain accessions on the main conclusions, PCA, ADMIXTURE, and FST analyses were repeated using only the 51 taxonomically confirmed accessions. The sensitivity analysis yielded results consistent with the full dataset in overall pattern: PCA showed the same extensive overlap between the two sections (**Supplementary Figure S2A**); ADMIXTURE cross-validation error reached a minimum at K = 5 (CV error = 0.5278) (**Supplementary Figure S2B**), at which all accessions again exhibited admixed ancestry components with no single component distinguishing the two sections (**Supplementary Figure S2C**). The mean FST was 0.038, approximately threefold higher than the estimate from the full 87-accession dataset (0.013) (**Supplementary Figure S2D**), although both values indicate low differentiation. These results indicate that the qualitative conclusion of limited genetic differentiation between the two sections is robust, while the point estimate of FST is sensitive to sample composition.

### 3.4 Phylogenetic relationships and pairwise genetic distances

The maximum-likelihood phylogenetic tree of 92 accessions (IQ-TREE 3.1.3, TVM + F + G4 model, 1,000 ultrafast bootstrap replicates) placed the five outgroup accessions at the base of the tree, forming Cluster I. Accessions of sect. *Eriandria* and sect. *Theopsis* were highly intermingled and did not form reciprocally monophyletic groups; instead, they were divided into four major clades (Clusters II–V), consistent with the population genetic structure analysis (**Figure 5**). Bootstrap support values for most major nodes ranged from 80% to 100%, indicating a generally reliable topology. Accessions of the same species generally clustered together.

**Figure 5:**
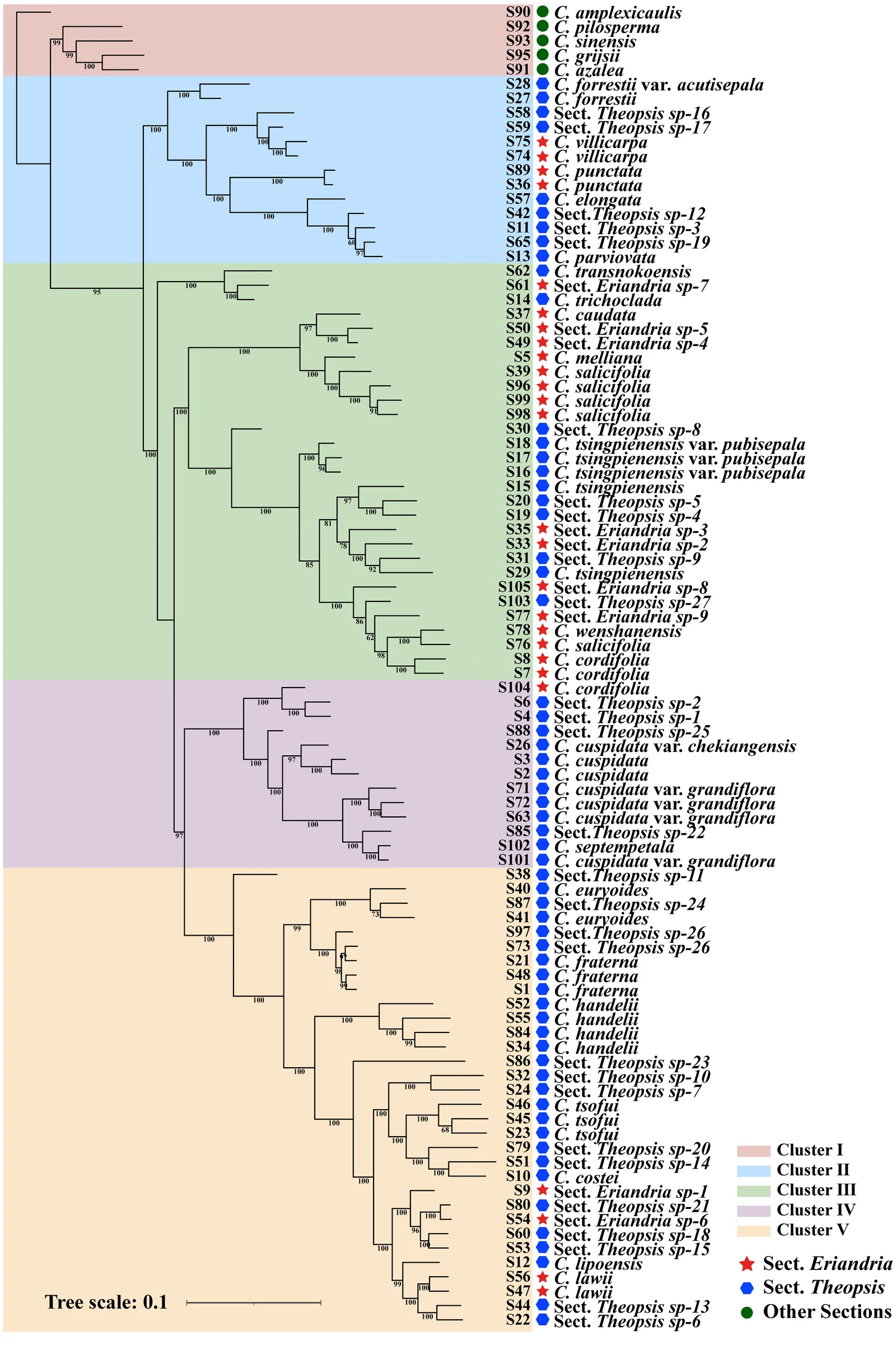
Rooted maximum-likelihood phylogenetic tree of 92 accessions of *Camellia*, including sect. *Eriandria*, sect. *Theopsis*, and five outgroup accessions from other sections. Branch support values are indicated at nodes; the tree was rooted using the five outgroup accessions.

To further quantify the genetic relationships suggested by the rooted phylogenetic tree, pairwise phylogenetic distances (patristic distances) among all 92 accessions were extracted from the tree and examined (**Figure 6**; **Supplementary Table S5**). S4 and S6 showed a relatively close relationship (0.0372), indicating that they may represent the same species with few phenotypic differences. The distances from S104 to S4 (0.0530) and S6 (0.0527) were nearly identical and slightly above the 0.05 threshold, suggesting that S104 was equally related to both accessions but more divergent than the S4–S6 pair. The four accessions of *C. fraterna* (S1, S21, S48, S73), all collected from Zhejiang Province, exhibited pairwise distances below 0.025 (0.0153–0.0231), forming the most compact intraspecific cluster detected. The genetic distance between *C. cuspidata* var. *grandiflora* (S101) and *C. septempetala* (S102) was extremely low (0.0159), providing genomic support for the treatment of *C. septempetala* as *C. cuspidata* var. *grandiflora* (Ming, 1999, 2000). Other pairs with distances below 0.05 included S58–S59 (0.0461), S49–S50 (0.0252), S19–S20 (0.0496), and S44–S22 (0.0368), indicating that these accessions are more consistent with conspecificity or intraspecific variation than with species-level distinctness.

**Figure 6:**
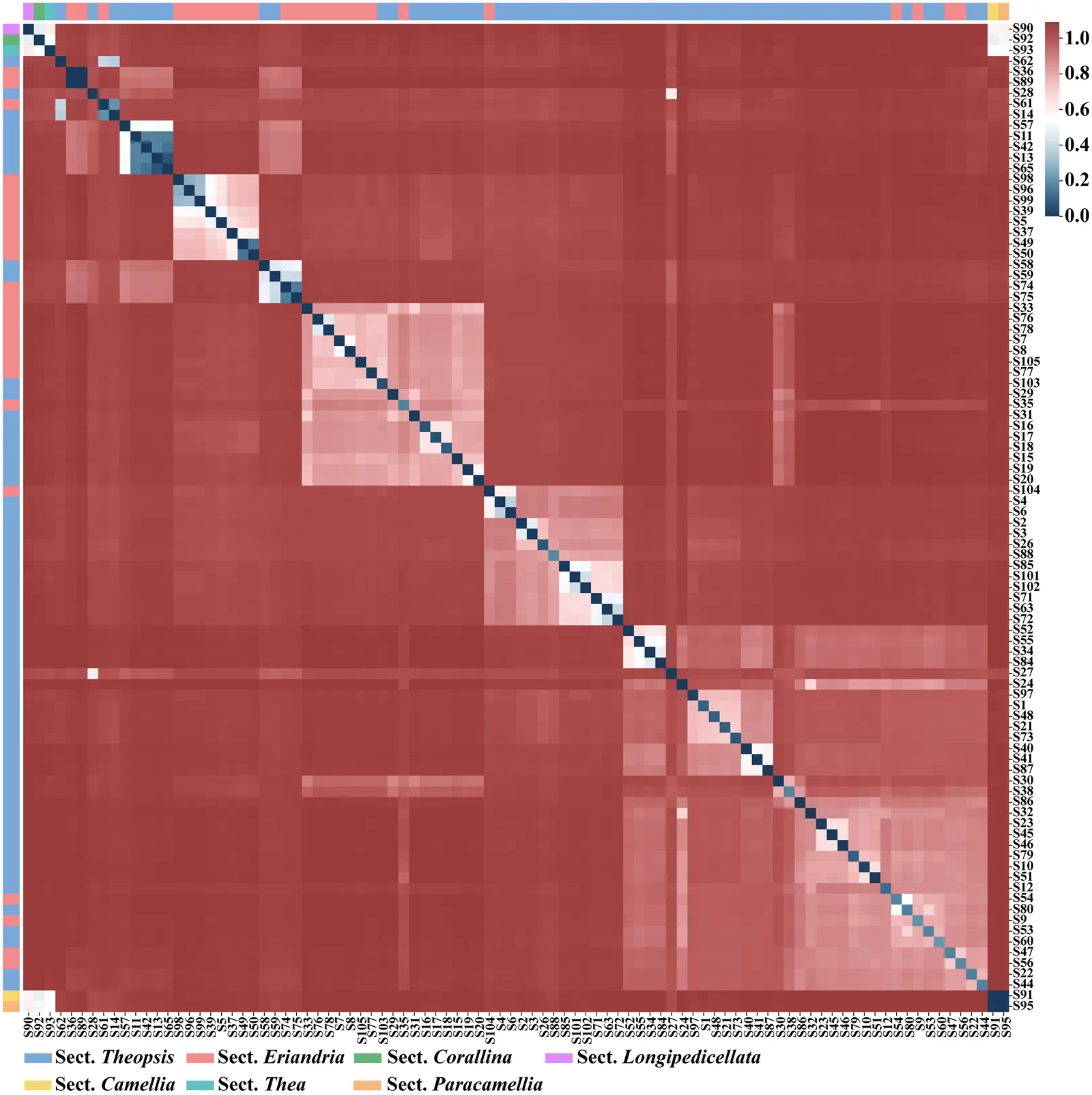
Heatmap of pairwise genetic distances (patristic distances) among 92 accessions of *Camellia*, including sect. *Eriandria*, sect. *Theopsis*, and five outgroup accessions from other sections.

Notably, *C. cordifolia* accession S104, collected from Hunan Province and the only sect. *Eriandria* accession in Cluster IV, did not group with the other *C. cordifolia* accessions (S7 and S8) from Guangdong Province, suggesting possible germplasm variation, geographic distance effects, or taxonomic uncertainty in this accession. Similarly, S76 (*C. salicifolia*) was separated from the other three accessions of *C. salicifolia* (S96, S98, S99), and some accessions with markedly different phenotypes were placed in close genetic proximity.

Based on the 58 focal accessions with native collection coordinates, Mantel tests revealed a significant positive correlation between genetic distance and geographic distance (Mantel r = 0.2211, P = 0.0010; **Figure 7A**), indicating a clear pattern of isolation by distance (IBD). A significant but weaker correlation was also detected between genetic distance and elevational difference (Mantel r = 0.056, P = 0.019; **Figure 7B**), suggesting that elevation may also contribute to genetic differentiation, although its effect is smaller than that of geographic distance.

**Figure 7:**
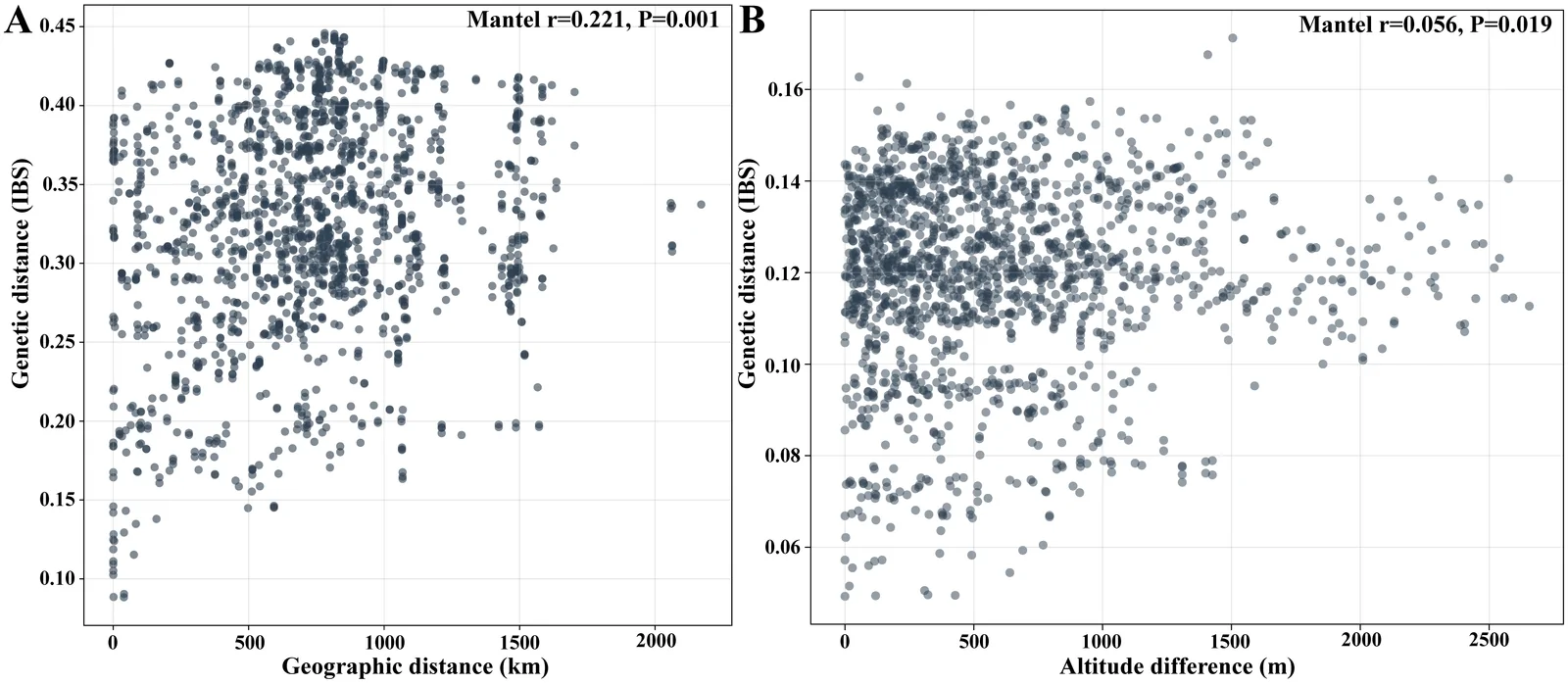
Relationship between genetic distance and geographic or elevational distance among the 58 focal accessions of *Camellia* sect. *Eriandria* and sect. *Theopsis*. **(A)** Scatter plot of genetic distance (IBS) versus geographic distance based on latitude and longitude. **(B)** Scatter plot of genetic distance (IBS) versus elevational difference.

### 3.5 Genome-wide selection signatures and candidate gene identification associated with OP

Grouping samples by OP phenotype (pubescent, n = 28; glabrous, n = 64), we performed genome-wide windowed FST scans (1 Mb windows, 100 kb steps, ≥5 SNPs per window). The top 1% threshold corresponded to an FST value of 0.0668, identifying 123 candidate windows and 137 candidate genes (**Figure 8A**). These windows showed a non-random distribution across the genome, with some chromosomal regions containing a disproportionate number of candidate windows. XP-CLR scans were conducted using the same window parameters, yielding 100 candidate windows and 113 candidate genes under the top 1% threshold (**Figure 8B**). The overlap between FST and XP-CLR candidate genes was 45 genes, highlighting genomic regions that may be subject to selection; however, these two scans are not statistically independent, and this overlap should be interpreted as a descriptive pattern rather than formal cross-validation.

**Figure 8:**
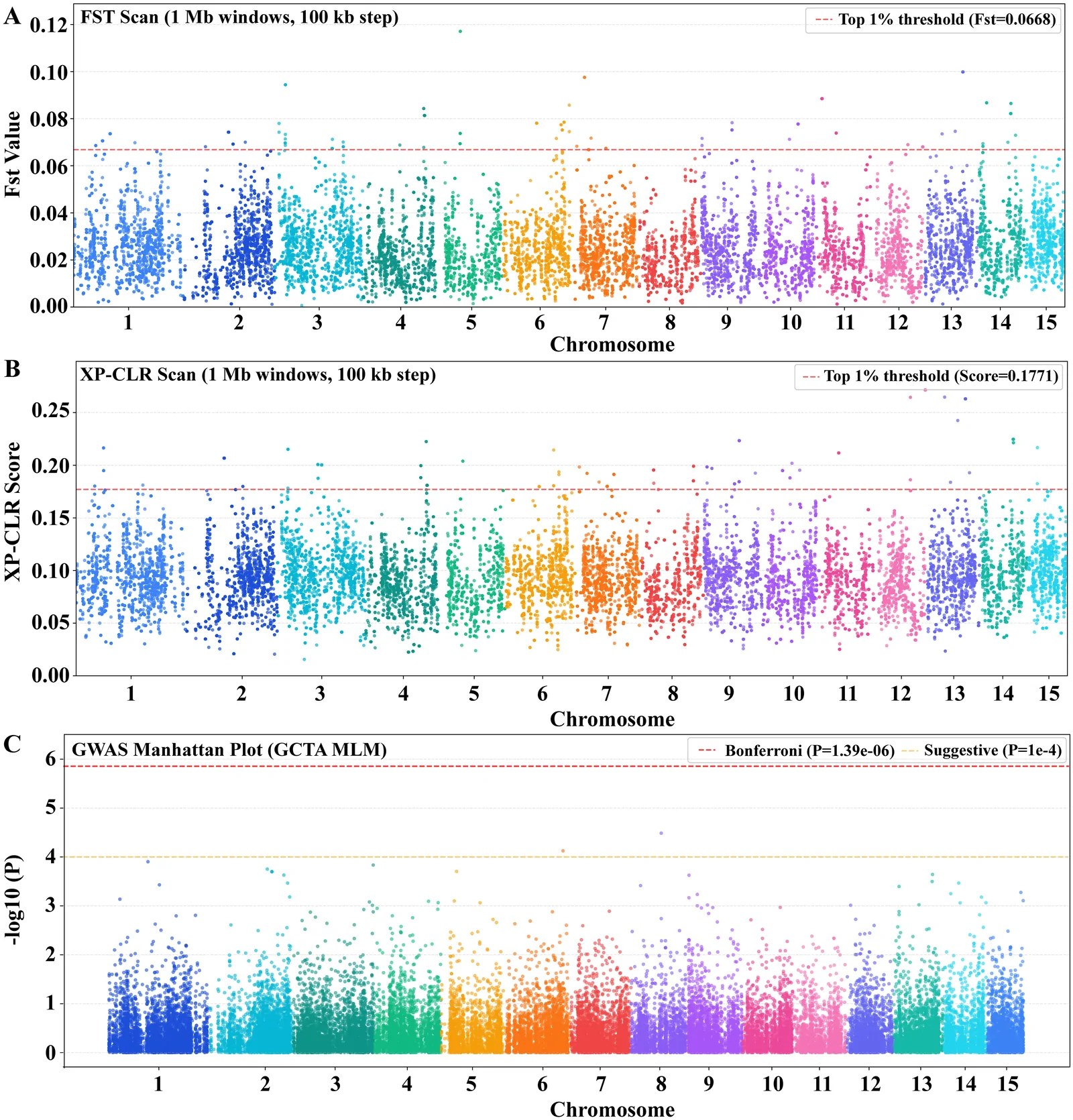
Genome-wide scans for loci associated with OP based on 92 accessions of *Camellia*, including sect. *Eriandria*, sect. *Theopsis*, and five outgroup accessions. **(A)** Manhattan plot of genome-wide FST values between accessions with pubescent and glabrous ovaries. **(B)** Manhattan plot of XP-CLR scores between accessions with pubescent and glabrous ovaries. **(C)** Manhattan plot of GWAS results for OP.

GCTA-based mixed-linear-model GWAS showed a genomic inflation factor λ = 0.808 (**Figure 8C**; **Supplementary Figure S3**), which is deflated relative to the expected value of 1 and may reflect model conservatism or overcorrection, potentially contributing to the absence of genome-wide significant signals. After Bonferroni correction (P <1.39 × 10^−6^), no genome-wide significant SNP was detected, likely due to limited statistical power from the modest sample size (n = 92). At the suggestive threshold (P <1 × 10^−4^), two suggestive SNPs were identified, corresponding to three candidate genes (**Figure 8C**), providing preliminary clues to the genetic basis of OP.

Integrating candidate genes from the three approaches yielded 208 unique candidate genes. Functional annotation of these genes using eggNOG-mapper revealed that, at the cellular component level, they were predominantly localized to the nucleus (n = 96), cytoplasm (n = 53), chloroplast (n = 39), and mitochondrion (n = 34) (**Figure 9A**), suggesting that candidate genes function across multiple subcellular compartments. In terms of biological process, the most frequently represented terms included embryo development (n = 9), response to cold (n = 9), response to abscisic acid (n = 9), protein phosphorylation (n = 8), defense response to bacterium (n = 8), and root development (n = 7) (**Figure 9B**), processes closely associated with plant growth, development, and abiotic/biotic stress responses. At the molecular function level, the predominant terms were protein binding (n = 84), mRNA binding (n = 18), RNA binding (n = 12), DNA binding (n = 12), and DNA-binding transcription factor activity (n = 10) (**Figure 9C**), indicating that the candidate genes are primarily involved in transcriptional and post-transcriptional regulation. KEGG pathway analysis revealed that the candidate genes were mainly distributed in carbohydrate metabolism (n = 18), metabolism of other amino acids (n = 13), lipid metabolism (n = 12), metabolism of cofactors and vitamins (n = 7), biosynthesis of other secondary metabolites (n = 6), and amino acid metabolism (n = 5). In environmental information processing, signal transduction pathways (n = 27) were the most frequently represented category (**Figure 10**).

**Figure 9:**
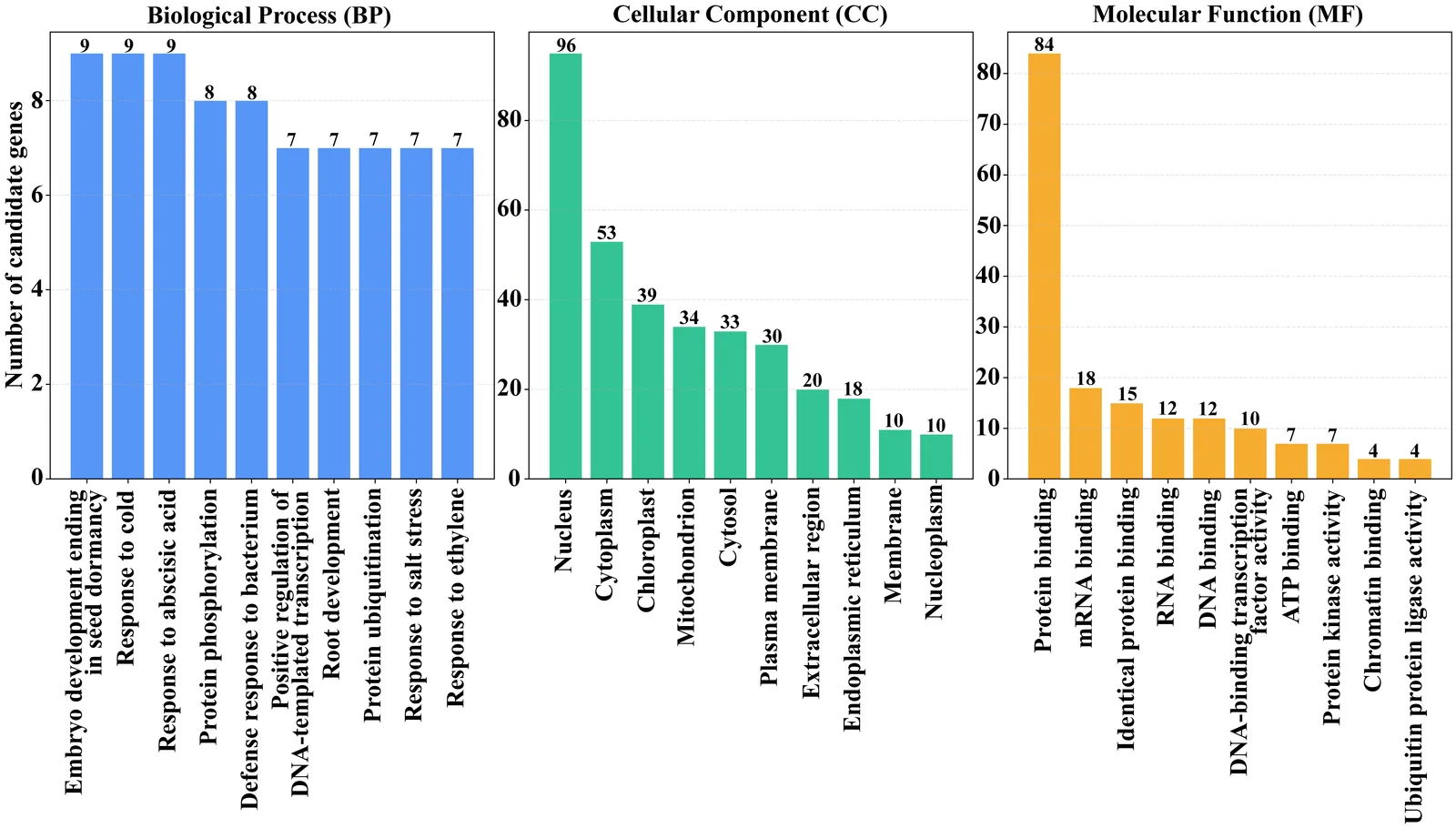
Gene Ontology (GO) annotation of candidate genes identified from genome-wide FST, XP-CLR, and GWAS scans for OP based on 92 accessions of *Camellia*, including sect. *Eriandria*, sect. *Theopsis*, and five outgroup accessions.

**Figure 10:**
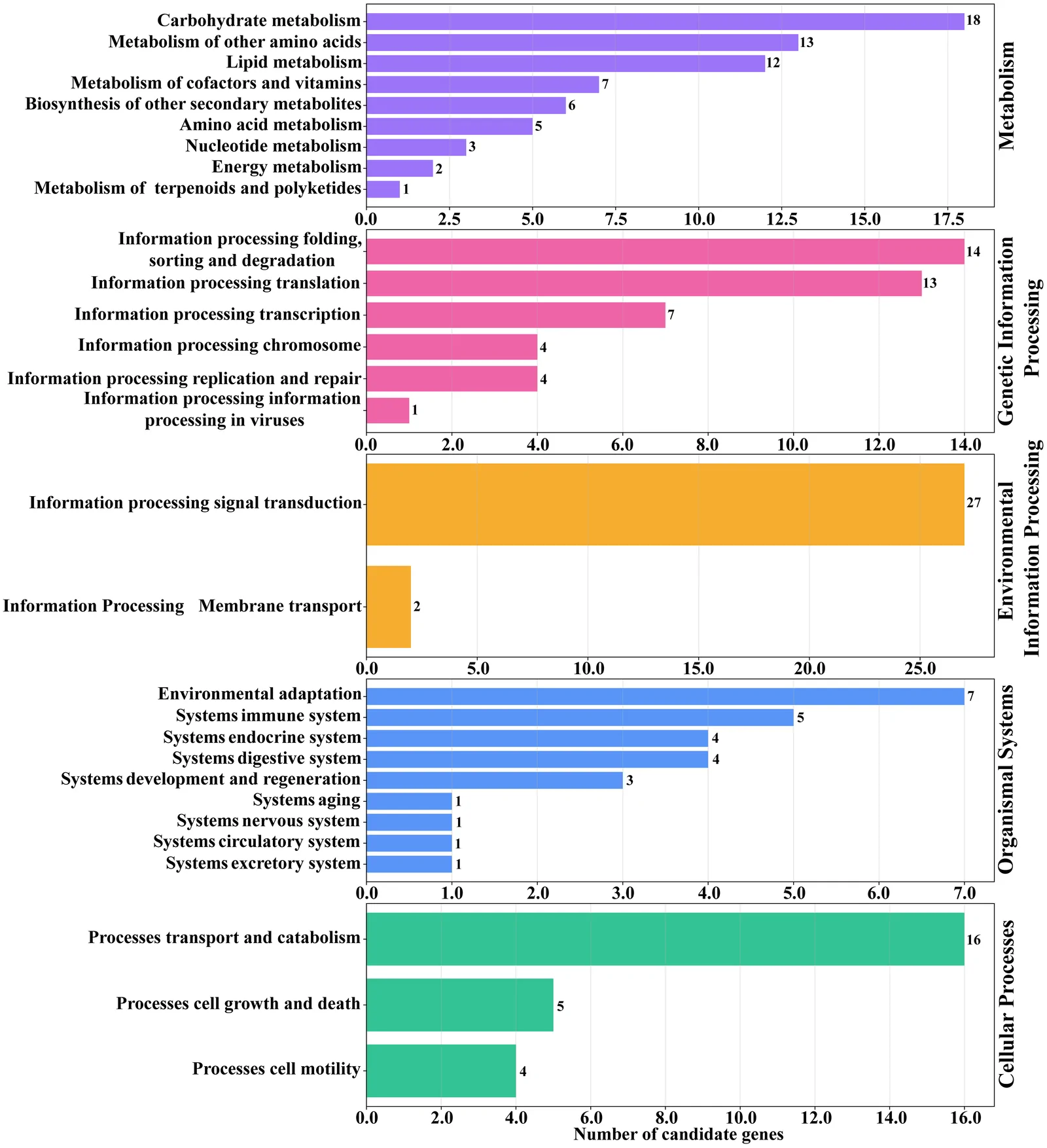
Kyoto Encyclopedia of Genes and Genomes (KEGG) pathway annotation of candidate genes identified from genome-wide FST, XP-CLR, and GWAS scans for OP based on 92 accessions of *Camellia*, including sect. *Eriandria*, sect. *Theopsis*, and five outgroup accessions.

Collectively, these functional features suggest that the genetic architecture of OP may involve coordinated actions of multiple biological processes, including hormone-related responses such as abscisic acid signaling, abiotic and biotic stress responses, and developmental regulation, rather than a single pathway. However, given the limited marker density (approximately 84 kb per marker), these candidate genes should be regarded as exploratory and require further validation through independent population studies and functional assays.

## 4 Discussion

### 4.1 Genetic structure and taxonomic re-evaluation

In the present study, population genetic analyses based on 35,885 high-quality SNPs revealed shared and complex ancestral components between the two sections (ADMIXTURE), extensive intermingling in PCA space, and interdigitated clustering patterns in the phylogenetic tree. Collectively, these results indicate the absence of well-delimited independent genetic groups and instead point to a continuous genetic lineage. Genetic distance analyses further demonstrated that conspecific individuals generally exhibited smaller genetic distances, whereas cross-section genetic distances overlapped with both intraspecific and interspecific distances, revealing no consistent pattern of intersectional differentiation. This pattern is consistent with previous studies based on chloroplast and nuclear ribosomal ITS sequences (Xiao and Parks, 2002; Vijayan et al., 2009) and also aligns with the proposal to merge the two sections based on morphological continuity (Ye, 1993).

The genome-wide mean FST between the two sections was 0.013 (95% CI: 0.0125–0.0129). As an empirical reference, Wright (1978) suggested that FST values below 0.05 indicate little genetic differentiation, and Hartl and Clark (1997) similarly regarded FST <0.05 as reflecting negligible differentiation. The FST value obtained in this study is well below this empirical benchmark, consistent with low genetic differentiation between the two sections. Within-population expected heterozygosity (HS = 0.230) was nearly equal to total expected heterozygosity (HT = 0.234), and the standardized differentiation indices G′ST = 0.016 and Jost’s D = 0.005 further confirmed the absence of a significant genetic barrier between the two sections. This FST value is substantially lower than the known interspecific differentiation levels within *Camellia* (Zan et al., 2023; Zhang et al., 2023), suggesting that sect. *Eriandria* and sect. *Theopsis* likely have not experienced prolonged geographical isolation or strong genetic barriers leading to complete reproductive isolation. They may instead constitute a continuous genetic group distributed across subtropical China, with populations maintaining genetic connectivity through pollen or seed dispersal.

The phylogenetic and pairwise distance patterns observed here have direct implications for the taxonomic treatment of sect. *Eriandria* and sect. *Theopsis*. The rooted tree, in which the five outgroup accessions formed a basal cluster and the focal accessions were divided into four clades without reciprocal monophyly, indicates that the two sections do not represent independent evolutionary lineages. This topology is consistent with the low genome-wide FST and admixed ancestry inferred from PCA and ADMIXTURE and collectively suggests that the sections should be recognized as a single, cohesive genetic group rather than separate taxa. The pairwise phylogenetic distances reinforce this interpretation. The extremely low distance between *C. cuspidata* var. *grandiflora* and *C. septempetala* (0.0159) falls within the range typically observed for conspecific individuals, supporting their synonymization. Likewise, the tight clustering of *C. fraterna* accessions from the same province illustrates that geographic proximity strongly influences genetic similarity, a pattern that must be considered when delimiting species within these groups. However, the exceptions observed—such as the separation of S104 from other *C. cordifolia* accessions and the separation of S76 from other *C. salicifolia* accessions—indicate that neither morphology nor geography alone fully explains the genetic structure. These discrepancies may reflect historical gene flow, incomplete lineage sorting, potential local adaptation, or even unrecognized cryptic diversity. From a taxonomic perspective, these findings argue against reliance on a single morphological character, particularly OP, and support an integrative approach that combines genomic, morphological, and geographic evidence for future revision of these sections.

In summary, this study supports the recognition of sect. *Eriandria* and sect. *Theopsis* as a continuous genetic group rather than two independent taxa. This finding challenges the traditional classification system that relies on OP as the diagnostic trait at the sectional level, suggesting that the observed difference in this trait may represent localized phenotypic divergence within a continuous genetic background. This conclusion is consistent with recent genome-wide phylogenomic studies of *Camellia* (Zan et al., 2023; Zhang et al., 2023), further highlighting the necessity of integrating genomic data to re-examine and revise traditional morphology-based taxonomic frameworks.

### 4.2 Localized selection signatures and ecological correlates

Despite the overall high genetic homogeneity between sect. *Eriandria* and sect. *Theopsis*, FST and XP-CLR analyses identified genomic regions showing signals of differentiation or positive selection. The top 1% FST scan identified 123 candidate windows (137 genes), and the top 1% XP-CLR scan identified 100 candidate windows (113 genes), with 45 genes overlapping between the two methods. This overlap highlights candidate genomic regions that may be subject to selection, although formal permutation testing is needed to determine whether the overlap exceeds random expectation.

Functional annotation revealed that the most frequently represented biological process terms included response to abscisic acid, response to cold, protein phosphorylation, defense response to bacterium, and embryo development. At the molecular function level, candidate genes were frequently associated with protein binding, mRNA binding, RNA binding, DNA binding, and DNA-binding transcription factor activity. These features suggest that the candidate genes are largely involved in hormone signaling, abiotic and biotic stress responses, and transcriptional or post-transcriptional regulation (Morris and Walker, 2003; Lehti-Shiu and Shiu, 2012; Cutler et al., 2010; Thomashow, 1999; Jones and Dangl, 2006). Collectively, these observations suggest that, against a background of pervasive gene flow, natural selection may act on specific loci involved in environmental perception and stress adaptation, potentially contributing to localized genomic differentiation (Lowry and Willis, 2010; Fournier-Level et al., 2011).

Geographic distribution patterns provide a macro-scale context consistent with the potential ecological relevance of OP. Sect. *Theopsis* exhibits a broader distribution range and a wider elevational span (700–2,000 m), whereas sect. *Eriandria* is predominantly distributed at mid-to-low elevations (260–850 m), suggesting differences in ecological niche breadth between the two sections. The significant IBD pattern (Mantel r = 0.2211, P = 0.0010) indicates that geographic distance is a primary driver of genetic differentiation. A weaker but still significant correlation between genetic distance and elevation (Mantel r = 0.056, P = 0.019) suggests that elevation may also contribute to genetic differentiation, although its effect is smaller than that of geographic distance. The OP trait itself may be of potential ecological relevance, with possible functions in seed protection, humidity regulation, or pest resistance, a hypothesis warranting further investigation.

### 4.3 Exploratory identification and functional inference of candidate genes

To connect selective signals with morphological divergence, we integrated results from GWAS, FST, and XP-CLR scans. The GWAS analysis (λ = 0.808) identified two suggestive SNPs (*P <*1 × 10^−4^) corresponding to three candidate genes, but no SNP reached genome-wide significance after Bonferroni correction (*P <*1.39 × 10^−6^). This result may reflect the polygenic genetic architecture of the OP trait, with multiple small-effect loci collectively contributing to phenotypic variation, and may also be constrained by the limited sample size (n = 92), which reduces statistical power to detect minor-effect loci.

Functional annotation of the 208 candidate genes indicated that functions related to stress responses, hormone signaling, and transcriptional or post-transcriptional regulation were predominant. These features suggest that the genetic basis of OP may involve coordinated actions of phytohormone signaling, abiotic and biotic stress responses, and transcriptional regulation (Morris and Walker, 2003; Lehti-Shiu and Shiu, 2012; Cutler et al., 2010; Thomashow, 1999; Jones and Dangl, 2006).

Among these candidate genes, the presence of DNA-binding transcription factor activity-related genes (n = 10) is consistent with the idea that transcriptional regulation may contribute to OP development. The detection of lipid metabolism pathways (n = 12) is also noteworthy, given that a recent comparative transcriptome study in tea plants (*C. sinensis*) identified a lipid transfer protein gene (*CsLTP*) as a candidate regulator of ovary trichome development (Wang et al., 2023). These parallels, although preliminary, highlight potential directions for future functional studies. However, given the limited marker density (approximately 84 kb per marker), the absence of genome-wide significant GWAS signals, and the fact that ploidy levels were not independently determined in this study, the causal relationships between these candidate genes and the OP phenotype should be interpreted cautiously and require further validation through independent population studies and functional assays. The contribution of this study lies in providing candidate targets for subsequent fine-mapping and functional studies, as well as genomic evidence for taxonomic revision within *Camellia*.

Sensitivity analysis further supports the robustness of the overall conclusions. PCA, ADMIXTURE, and FST analyses repeated on the 51 taxonomically confirmed accessions yielded results consistent with the full dataset in terms of overall pattern, indicating that the inclusion of taxonomically uncertain accessions did not substantially affect the main conclusions.

## 5 Conclusions

In this study, we generated genome-wide SNP data for 87 wild accessions of *Camellia* sect. *Eriandria* and sect. *Theopsis*, together with five outgroup accessions, via super-GBS sequencing, establishing a foundational genomic resource. Our findings reveal extensive admixture and a continuous gene pool between the two sections. The extremely low level of genetic differentiation (FST ≈ 0.013, 95% CI: 0.0125–0.0129; HS = 0.230, HT = 0.234; G′ST = 0.016; Jost’s D = 0.005) supports their recognition as a cohesive genetic group rather than two independent taxonomic units. These results indicate that the traditional reliance on OP as the sole diagnostic trait is insufficient to reflect their true phylogenetic relationships, highlighting the necessity of integrating genomic data to complement traditional morphology-based classification.

Despite pervasive gene flow, we detected localized genomic regions showing signals of selection. Functional annotation indicated that the candidate genes were most frequently associated with responses to abscisic acid, cold, and biotic stress, as well as protein phosphorylation and transcriptional regulation, suggesting that natural selection may have acted on loci involved in environmental perception and stress adaptation. Integration of FST, XP-CLR, and GWAS yielded 208 candidate genes from OP-stratified genome-wide scans. However, given the limited marker density (approximately 84 kb per marker), the absence of genome-wide significant GWAS signals, and the fact that ploidy levels were not independently determined, these candidate genes should be regarded as exploratory and require further validation through independent population studies and functional assays.

In conclusion, this study provides a valuable genomic resource for future research on these germplasm, offers robust genomic evidence supporting the taxonomic merger of sect. *Eriandria* and sect. *Theopsis*, and lays a foundation for the conservation and breeding of these materials.

## Data Availability

The datasets presented in this study can be found in online repositories. The names of the repository/repositories and accession number(s) can be found below: https://bigd.big.ac.cn/gvm/getProjectDetail?Project=GVM001549, GVM001549.
