# Supplementary material for: Genomic evidence supports the merging of Camellia sect. Eriandria and sect. Theopsis: ovary pubescence as a trait of potential ecological relevance
Source: Front Plant Sci. 2026 Sep 24;17:1932891. doi: 10.3389/fpls.2026.1932891 (PMC13646152; doi:10.3389/fpls.2026.1932891)
Supplement: Supplementary file 1 [file DataSheet1.docx]

Supplementary Material

# 1 Supplementary Figures

**FIGURE S1** Data quality assessment and linkage disequilibrium decay. (A) Scatter plot of per-sample divergence index versus missing-data rate. (B) Scatter plot of per-sample divergence index versus observed heterozygosity. (C) Genome-wide linkage disequilibrium (LD) decay estimated from the 87 focal accessions. Mean r² values were calculated for SNP pairs within 500 kb and averaged over 1 kb bins. Spearman correlation coefficients (ρ) and P-values are shown in (A) and (B).

**FIGURE S2** Sensitivity analysis of population genetic structure and genome-wide differentiation based on 51 taxonomically confirmed accessions of *Camellia* sect. *Eriandria* and sect. *Theopsis*. (A) Three-dimensional principal component analysis (PCA) of the 51 confirmed accessions; ellipses denote 95% confidence regions. (B) Cross-validation error as a function of K, estimated from the 51 confirmed accessions, used to determine the optimal number of ancestral populations. (C) Model-based clustering of the 51 confirmed accessions at the optimal K, inferred using ADMIXTURE. (D) Genome-wide FST between sect. *Eriandria* and sect. *Theopsis*, calculated from the 51 confirmed accessions.

**FIGURE S3** Q-Q plot of observed vs. expected -log₁₀(*p*) values from the GWAS. The genomic inflation factor (λ) was 0.808.

# 2 Supplementary Tables

Table S1 List of 92 accessions of *Camellia* used for population genomic analyses, including 87 focal accessions (sect. *Eriandria* and sect. *Theopsis*) and five outgroup accessions from other sections, with locality information and ovary pubescence (OP) phenotype.

| **Group** | **Number** | **Sample ID** | **Accessions** | **Origin** | | **Collected from native habitat** | **Longitude (°E)** | **Latitude (°N)** | **Elevation (m)** | **OP** |
| --- | --- | --- | --- | --- | --- | --- | --- | --- | --- | --- |
| **Sect. *Eriandria*** | |  |  | |  |  |  |  |  |  |
|  | 1 | S5 | *C. melliana* | Huizhou, Guangdong Province | | Yes | 113.49 | 23.39 | 723 | 1 |
|  | 2 | S7 | *C. cordifolia* | Shaoguan, Guangdong Province | | Yes | 113.28 | 24.78 | 845 | 1 |
|  | 3 | S8 | *C. cordifolia* | Shaoguan, Guangdong Province | | Yes | 113.28 | 24.78 | 937 | 1 |
|  | 4 | S9 | Sect. *Eriandria* sp-1 | Enshi, Hubei Province | | Yes | 108.94 | 30.30 | 818 | 1 |
|  | 5 | S33 | Sect. *Eriandria* sp-2 | Wenshan, Yunnan Province | | Yes | 104.41 | 23.21 | 1687 | 1 |
|  | 6 | S35 | Sect. *Eriandria* sp-3 | Jinhua, Zhejiang Province | | No | 119.60 | 29.13 | - | 1 |
|  | 7 | S36 | *C. punctata* | Guangzhou, Guangdong Province | | No | 113.33 | 23.16 | - | 1 |
|  | 8 | S37 | *C. caudata* | Jinhua, Zhejiang Province | | No | 119.60 | 29.13 | - | 1 |
|  | 9 | S39 | *C. salicifolia* | Guangzhou, Guangdong Province | | No | 113.33 | 23.16 | - | 1 |
|  | 10 | S47 | *C. lawii* | Jinhua, Zhejiang Province | | No | 119.60 | 29.13 | - | 1 |
|  | 11 | S49 | Sect. *Eriandria* sp-4 | Nanning, Guangxi Province | | Yes | 108.22 | 23.31 | 968 | 1 |
|  | 12 | S50 | Sect. *Eriandria* sp-5 | Nanning, Guangxi Province | | Yes | 108.19 | 23.32 | 199 | 1 |
|  | 13 | S54 | Sect. *Eriandria* sp-6 | Changyang, Hubei Province | | Yes | 110.44 | 30.38 | 1190 | 1 |
|  | 14 | S56 | *C. lawii* | Beibei, Chongqing | | Yes | 106.38 | 29.83 | 708 | 1 |
|  | 15 | S61 | Sect. *Eriandria* sp-7 | Jinhua, Zhejiang Province | | No | 119.60 | 29.13 | - | 1 |
|  | 16 | S74 | *C. villicarpa* | Chengdu, Sichuan Province | | Yes | 103.70 | 30.16 | 983 | 1 |
|  | 17 | S75 | *C. villicarpa* | Chengdu, Sichuan Province | | Yes | 103.70 | 30.16 | 1000 | 1 |
|  | 18 | S76 | *C. salicifolia* | Nanping, Fujian Province | | Yes | 118.07 | 26.41 | 450 | 1 |
|  | 19 | S78 | *C. wenshanensis* | Jinhua, Zhejiang Province | | No | 119.60 | 29.13 | - | 1 |
|  | 20 | S89 | *C. punctata* | Emeishan, Sichuan Province | | Yes | 103.46 | 29.32 | 335 | 1 |
|  | 21 | S96 | *C. salicifolia* | Nanping, Fujian Province | | Yes | 118.12 | 26.69 | 263 | 1 |
|  | 22 | S98 | *C. salicifolia* | Nanping, Fujian Province | | Yes | 118.12 | 26.69 | 292 | 1 |
|  | 23 | S99 | *C. salicifolia* | Nanping, Fujian Province | | Yes | 118.12 | 26.69 | 407 | 1 |
|  | 24 | S104 | *C. cordifolia* | Yongzhou, Hunan Province | | Yes | 111.48 | 25.59 | 466 | 1 |
|  | 25 | S105 | Sect. *Eriandria* sp-8 | Guilin, Guangxi Province | | Yes | 110.12 | 25.55 | 724 | 1 |
|  | 26 | S77 | Sect. *Eriandria* sp-9 | Nanping, Fujian Province | | Yes | 118.07 | 26.41 | 450 | 1 |
| **Sect. *Theopsis*** | |  |  | |  |  |  |  |  |  |
|  | 1 | S1 | *C. fraterna* | Hangzhou, Zhejiang Province | | No | 119.73 | 30.24 | - | 0 |
|  | 2 | S2 | *C. cuspidata* | Hangzhou, Zhejiang Province | | No | 119.73 | 30.24 | - | 0 |
|  | 3 | S3 | *C. cuspidata* | Hangzhou, Zhejiang Province | | No | 119.73 | 30.24 | - | 0 |
|  | 4 | S4 | Sect. *Theopsis* sp-1 | Shaoguan, Guangdong Province | | Yes | 112.56 | 24.41 | 845 | 0 |
|  | 5 | S6 | Sect. *Theopsis* sp-2 | Shaoguan, Guangdong Province | | Yes | 112.56 | 24.41 | 845 | 0 |
|  | 6 | S10 | *C. costei* | Guiyang, Guizhou Province | | Yes | 106.29 | 26.40 | 1592 | 0 |
|  | 7 | S11 | Sect. *Theopsis* sp-3 | Emeishan, Sichuan Province | | Yes | 103.23 | 29.34 | 770 | 0 |
|  | 8 | S12 | *C. lipoensis* | Duyun, Guizhou Province | | Yes | 108.40 | 25.17 | 462 | 0 |
|  | 9 | S13 | *C. parviovata* | Emeishan, Sichuan Province | | Yes | 103.49 | 29.61 | 889 | 0 |
|  | 10 | S14 | *C. trichoclada* | Wenzhou, Zhejiang Province | | No | 120.71 | 28.00 | - | 0 |
|  | 11 | S15 | *C. tsingpienensis* | Liuzhou, Guangxi Province | | Yes | 109.80 | 25.23 | 965 | 0 |
|  | 12 | S16 | *C. tsingpienensis* var. *pubisepala* | Nanning, Guangxi Province | | Yes | 108.22 | 23.31 | 1027 | 0 |
|  | 13 | S17 | *C. tsingpienensis* var. *pubisepala* | Nanning, Guangxi Province | | Yes | 108.22 | 23.31 | 1043 | 0 |
|  | 14 | S18 | *C. tsingpienensis* var. *pubisepala* | Nanning, Guangxi Province | | Yes | 108.22 | 23.31 | 1048 | 0 |
|  | 15 | S19 | Sect. *Theopsis sp*-4 | Baise, Guangxi Province | | Yes | 106.23 | 24.29 | 1759 | 0 |
|  | 16 | S20 | Sect. *Theopsis sp*-5 | Baise, Guangxi Province | | Yes | 106.23 | 24.29 | 1759 | 0 |
|  | 17 | S21 | *C. fraterna* | Jinhua, Zhejiang Province | | No | 119.60 | 29.13 | - | 0 |
|  | 18 | S22 | Sect. *Theopsis sp*-6 | Emeishan, Sichuan Province | | Yes | 103.26 | 29.33 | 619 | 0 |
|  | 19 | S23 | *C. tsofui* | Beibei, Chongqing | | No | 106.38 | 29.83 | - | 0 |
|  | 20 | S24 | Sect. *Theopsis* sp-7 | Jinhua, Zhejiang Province | | No | 119.60 | 29.13 | - | 0 |
|  | 21 | S26 | *C. cuspidata* var. *chekiangensis* | Wenzhou, Zhejiang Province | | No | 120.71 | 28.00 | - | 0 |
|  | 22 | S27 | *C. forrestii* | Wenshan, Yunnan Province | | Yes | 103.55 | 23.22 | 2739 | 0 |
|  | 23 | S28 | *C. forrestii* var *acutisepala* | Wenshan, Yunnan Province | | Yes | 103.54 | 23.22 | 2855 | 0 |
|  | 24 | S29 | *C. tsingpienensis* | Mengzi, Yunnan Province | | Yes | 103.13 | 22.51 | 1850 | 0 |
|  | 25 | S30 | Sect. *Theopsis* sp-8 | Wenshan, Yunnan Province | | Yes | 104.01 | 22.51 | 1840 | 0 |
|  | 26 | S31 | Sect. *Theopsis* sp-9 | Wenshan, Yunnan Province | | Yes | 104.01 | 22.51 | 1876 | 0 |
|  | 27 | S32 | Sect. *Theopsis* sp-10 | Wenshan, Yunnan Province | | Yes | 104.41 | 23.21 | 1655 | 0 |
|  | 28 | S34 | *C. handelii* | Changsha, Hunan Province | | No | 112.94 | 28.19 | - | 0 |
|  | 29 | S38 | Sect. *Theopsis* sp-11 | Mengzi, Yunnan Province | | Yes | 103.13 | 22.51 | 1743 | 0 |
|  | 30 | S40 | *C. euryoides* | Jinhua, Zhejiang Province | | No | 119.60 | 29.13 | - | 0 |
|  | 31 | S41 | *C. euryoides* | Jinhua, Zhejiang Province | | No | 119.60 | 29.13 | - | 0 |
|  | 32 | S42 | Sect. *Theopsis* sp-12 | Emeishan, Sichuan Province | | Yes | 103.22 | 29.34 | 1198 | 0 |
|  | 33 | S44 | Sect. *Theopsis* sp-13 | Emeishan, Sichuan Province | | Yes | 103.24 | 29.34 | 776 | 0 |
|  | 34 | S45 | *C. tsofui* | Beibei, Chongqing | | Yes | 106.38 | 29.83 | 811 | 0 |
|  | 35 | S46 | *C. tsofui* | Beibei, Chongqing | | Yes | 106.38 | 29.83 | 814 | 0 |
|  | 36 | S48 | *C. fraterna* | Jinhua, Zhejiang Province | | No | 119.60 | 29.13 | - | 0 |
|  | 37 | S51 | Sect. *Theopsis* sp-14 | Enshi, Hubei Province | | Yes | 109.25 | 30.19 | 1100 | 0 |
|  | 38 | S52 | *C. handelii* | Qingyuan, Guangdong Province | | Yes | 113.05 | 24.28 | 553 | 0 |
|  | 39 | S53 | Sect. *Theopsis* sp-15 | Changyang, Hubei Province | | Yes | 110.44 | 30.38 | 1190 | 0 |
|  | 40 | S55 | *C. handelii* | Changsha, Hunan Province | | No | 112.94 | 28.19 | - | 0 |
|  | 41 | S57 | *C. elongata* | Emeishan, Sichuan Province | | Yes | 103.22 | 29.34 | 1265 | 0 |
|  | 42 | S58 | Sect. *Theopsis* sp-16 | Yichang, Hubei Province | | No | 111.29 | 30.70 | - | 0 |
|  | 43 | S59 | Sect. *Theopsis* sp-17 | Yichang, Hubei Province | | No | 111.29 | 30.70 | - | 0 |
|  | 44 | S60 | Sect. *Theopsis* sp-18 | Emeishan, Sichuan Province | | Yes | 103.20 | 29.34 | 1545 | 0 |
|  | 45 | S62 | *C. transnokoensis* | Jinhua, Zhejiang Province | | No | 119.65 | 29.08 | - | 0 |
|  | 46 | S63 | *C. cuspidata* var. *grandiflora* | Enshi, Hubei Province | | No | 108.94 | 30.30 | - | 0 |
|  | 47 | S65 | Sect. *Theopsis* sp-19 | Emeishan, Sichuan Province | | No | 103.39 | 29.60 | - | 0 |
|  | 48 | S71 | *C. cuspidata* var. *grandiflora* | Changyang, Hubei Province | | Yes | 110.44 | 30.38 | 1190 | 0 |
|  | 49 | S72 | *C. cuspidata* var. *grandiflora* | Enshi, Hubei Province | | No | 108.94 | 30.30 | - | 0 |
|  | 50 | S73 | *C. fraterna* | Lishui, Zhejiang Province | | Yes | 119.19 | 27.29 | 575 | 0 |
|  | 51 | S79 | Sect. *Theopsis* sp-20 | Zunyi, Guizhou Province | | Yes | 107.36 | 29.07 | 1220 | 0 |
|  | 52 | S80 | Sect. *Theopsis* sp-21 | Changyang, Hubei Province | | Yes | 110.84 | 30.61 | 550 | 0 |
|  | 53 | S84 | *C. handelii* | Wuhan, Hubei Province | | No | 114.43 | 30.55 | - | 0 |
|  | 54 | S85 | Sect. *Theopsis* sp-22 | Yongzhou, Hunan Province | | Yes | 111.92 | 26.10 | 1367 | 0 |
|  | 55 | S86 | Sect. *Theopsis* sp-23 | Yingjiang, Yunnan Province | | Yes | 97.56 | 25.03 | 925 | 0 |
|  | 56 | S87 | Sect. *Theopsis* sp-24 | Zhuzhou, Hunan Province | | No | 113.93 | 25.99 | - | 0 |
|  | 57 | S88 | Sect. *Theopsis* sp-25 | Zhuzhou, Hunan Province | | No | 113.83 | 25.85 | - | 0 |
|  | 58 | S97 | Sect. *Theopsis* sp-26 | Nanping, Fujian Province | | Yes | 118.12 | 26.69 | 280 | 0 |
|  | 59 | S101 | *C. cuspidata* var. *grandiflora* | Shaoyang, Hunan Province | | Yes | 110.34 | 26.32 | 677 | 0 |
|  | 60 | S102 | *C.* *septempetala* | Shaoyang, Hunan Province | | Yes | 111.10 | 26.25 | 999 | 0 |
|  | 61 | S103 | Sect. *Theopsis* sp-27 | Yongzhou, Hunan Province | | Yes | 112.14 | 25.15 | 776 | 0 |
| Sect. *Longipedicellata* | | S90 | *C. amplexicaulis* | Jinhua, Zhejiang Province | | No | - | - | - | 0 |
| Sect. *Camellia* | | S91 | *C. azalea* | Jinhua, Zhejiang Province | | No | - | - | - | 0 |
| Sect. *Corallina* | | S92 | *C. pilosperma* | Hezhou, Guangzhou Province | | Yes | - | - | - | 1 |
| Sect. *Thea* | | S93 | *C. sinensis* | Xinyang, Henan Province | | Yes | - | - | - | 0 |
| Sect. *Paracamellia* | | S95 | *C. grijsii* | Kunming, Yunan Province | | No | - | - | - | 1 |

Note: The accessions followed Chang’s classification system (Chang, 1981).

Table S2 Summary of sequencing and mapping statistics for *Camellia* sect. *Eriandria*, sect. *Theopsis*, and outgroup accessions.

| **Accessions** | **Number of accessions** | **Mean mapping rate** |
| --- | --- | --- |
| Sect. *Eriandria* | 26 | 93.25% |
| Sect. *Theopsis* | 61 | 96.07% |
| Outgroup accessions | 5 | 96.96% |

Table S3 Summary of genome-wide SNP statistics, substitution patterns, and genomic feature distribution.

| **Transversion** | **Number** | **Transition** | **Numbers** | **Genomic Features** | **Number** |
| --- | --- | --- | --- | --- | --- |
| A→C | 1339 | A→G | 6010 | Upstream | 1430 |
| A→T | 1563 | C→T | 6767 | Splicing | 29 |
| C→A | 1240 | G→A | 6889 | Exonic | 12293 |
| C→G | 973 | T→C | 6003 | Intronic | 12217 |
| G→C | 936 |  |  | Downstream | 2944 |
| G→T | 1220 |  |  | Intergenic | 6972 |
| T→A | 1545 |  |  |  |  |
| T→G | 1400 |  |  |  |  |

Table S4 Pairwise identity-by-state (IBS) distance matrix among 92 accessions of *Camellia*, including 87 focal accessions (sect. *Eriandria* and sect. *Theopsis*) and five outgroup accessions from other sections.

| Sample ID | S1 | S10 | S101 | S102 | S103 | S104 | S105 | S11 | S12 | S13 | S14 | S15 | S16 | S17 | S18 | S19 | S2 | S20 | S21 |
| --- | --- | --- | --- | --- | --- | --- | --- | --- | --- | --- | --- | --- | --- | --- | --- | --- | --- | --- | --- |
| S1 | 0.0000 | 0.3521 | 0.3763 | 0.3767 | 0.4215 | 0.3595 | 0.4152 | 0.3944 | 0.3306 | 0.3964 | 0.3441 | 0.4187 | 0.4247 | 0.4230 | 0.4239 | 0.4206 | 0.3589 | 0.4208 | 0.2145 |
| S10 | 0.3521 | 0.0000 | 0.3575 | 0.3525 | 0.3954 | 0.3405 | 0.3791 | 0.3427 | 0.2767 | 0.3427 | 0.3227 | 0.3777 | 0.4158 | 0.4126 | 0.4154 | 0.3746 | 0.3322 | 0.3790 | 0.3490 |
| S101 | 0.3763 | 0.3575 | 0.0000 | 0.1152 | 0.3331 | 0.2077 | 0.3189 | 0.3185 | 0.3988 | 0.3189 | 0.2752 | 0.3341 | 0.3627 | 0.3661 | 0.3661 | 0.3334 | 0.2020 | 0.3382 | 0.3773 |
| S102 | 0.3767 | 0.3525 | 0.1152 | 0.0000 | 0.3278 | 0.2051 | 0.3113 | 0.3110 | 0.4000 | 0.3115 | 0.2721 | 0.3274 | 0.3610 | 0.3621 | 0.3636 | 0.3267 | 0.1982 | 0.3324 | 0.3759 |
| S103 | 0.4215 | 0.3954 | 0.3331 | 0.3278 | 0.0000 | 0.3089 | 0.1685 | 0.3328 | 0.4243 | 0.3326 | 0.2889 | 0.1928 | 0.2386 | 0.2377 | 0.2342 | 0.2078 | 0.3206 | 0.2084 | 0.4220 |
| S104 | 0.3595 | 0.3405 | 0.2077 | 0.2051 | 0.3089 | 0.0000 | 0.2973 | 0.2967 | 0.3818 | 0.2988 | 0.2556 | 0.3087 | 0.3390 | 0.3410 | 0.3425 | 0.3098 | 0.2115 | 0.3150 | 0.3601 |
| S105 | 0.4152 | 0.3791 | 0.3189 | 0.3113 | 0.1685 | 0.2973 | 0.0000 | 0.3165 | 0.4133 | 0.3147 | 0.2763 | 0.1801 | 0.2365 | 0.2340 | 0.2345 | 0.1920 | 0.2977 | 0.1973 | 0.4135 |
| S11 | 0.3944 | 0.3427 | 0.3185 | 0.3110 | 0.3328 | 0.2967 | 0.3165 | 0.0000 | 0.3943 | 0.0884 | 0.2567 | 0.3203 | 0.3600 | 0.3604 | 0.3592 | 0.3143 | 0.2892 | 0.3196 | 0.3922 |
| S12 | 0.3306 | 0.2767 | 0.3988 | 0.4000 | 0.4243 | 0.3818 | 0.4133 | 0.3943 | 0.0000 | 0.3940 | 0.3643 | 0.4132 | 0.4271 | 0.4265 | 0.4269 | 0.4135 | 0.3795 | 0.4154 | 0.3303 |
| S13 | 0.3964 | 0.3427 | 0.3189 | 0.3115 | 0.3326 | 0.2988 | 0.3147 | 0.0884 | 0.3940 | 0.0000 | 0.2557 | 0.3222 | 0.3575 | 0.3596 | 0.3592 | 0.3163 | 0.2890 | 0.3207 | 0.3940 |
| S14 | 0.3441 | 0.3227 | 0.2752 | 0.2721 | 0.2889 | 0.2556 | 0.2763 | 0.2567 | 0.3643 | 0.2557 | 0.0000 | 0.2795 | 0.3110 | 0.3097 | 0.3137 | 0.2772 | 0.2496 | 0.2806 | 0.3424 |
| S15 | 0.4187 | 0.3777 | 0.3341 | 0.3274 | 0.1928 | 0.3087 | 0.1801 | 0.3203 | 0.4132 | 0.3222 | 0.2795 | 0.0000 | 0.2250 | 0.2210 | 0.2192 | 0.1659 | 0.3011 | 0.1663 | 0.4178 |
| S16 | 0.4247 | 0.4158 | 0.3627 | 0.3610 | 0.2386 | 0.3390 | 0.2365 | 0.3600 | 0.4271 | 0.3575 | 0.3110 | 0.2250 | 0.0000 | 0.1841 | 0.1863 | 0.2244 | 0.3406 | 0.2287 | 0.4236 |
| S17 | 0.4230 | 0.4126 | 0.3661 | 0.3621 | 0.2377 | 0.3410 | 0.2340 | 0.3604 | 0.4265 | 0.3596 | 0.3097 | 0.2210 | 0.1841 | 0.0000 | 0.1838 | 0.2224 | 0.3400 | 0.2273 | 0.4248 |
| S18 | 0.4239 | 0.4154 | 0.3661 | 0.3636 | 0.2342 | 0.3425 | 0.2345 | 0.3592 | 0.4269 | 0.3592 | 0.3137 | 0.2192 | 0.1863 | 0.1838 | 0.0000 | 0.2259 | 0.3420 | 0.2253 | 0.4233 |
| S19 | 0.4206 | 0.3746 | 0.3334 | 0.3267 | 0.2078 | 0.3098 | 0.1920 | 0.3143 | 0.4135 | 0.3163 | 0.2772 | 0.1659 | 0.2244 | 0.2224 | 0.2259 | 0.0000 | 0.3027 | 0.1459 | 0.4182 |
| S2 | 0.3589 | 0.3322 | 0.2020 | 0.1982 | 0.3206 | 0.2115 | 0.2977 | 0.2892 | 0.3795 | 0.2890 | 0.2496 | 0.3011 | 0.3406 | 0.3400 | 0.3420 | 0.3027 | 0.0000 | 0.3072 | 0.3583 |
| S20 | 0.4208 | 0.3790 | 0.3382 | 0.3324 | 0.2084 | 0.3150 | 0.1973 | 0.3196 | 0.4154 | 0.3207 | 0.2806 | 0.1663 | 0.2287 | 0.2273 | 0.2253 | 0.1459 | 0.3072 | 0.0000 | 0.4212 |
| S21 | 0.2145 | 0.3490 | 0.3773 | 0.3759 | 0.4220 | 0.3601 | 0.4135 | 0.3922 | 0.3303 | 0.3940 | 0.3424 | 0.4178 | 0.4236 | 0.4248 | 0.4233 | 0.4182 | 0.3583 | 0.4212 | 0.0000 |
| S22 | 0.3325 | 0.2642 | 0.3901 | 0.3892 | 0.4230 | 0.3688 | 0.4110 | 0.3706 | 0.2613 | 0.3694 | 0.3529 | 0.4109 | 0.4278 | 0.4273 | 0.4304 | 0.4088 | 0.3674 | 0.4117 | 0.3326 |
| S23 | 0.3441 | 0.1904 | 0.3534 | 0.3490 | 0.3893 | 0.3368 | 0.3735 | 0.3387 | 0.2815 | 0.3378 | 0.3157 | 0.3747 | 0.4089 | 0.4121 | 0.4111 | 0.3719 | 0.3259 | 0.3768 | 0.3409 |
| S24 | 0.3459 | 0.1935 | 0.3480 | 0.3440 | 0.3853 | 0.3286 | 0.3682 | 0.3324 | 0.2704 | 0.3323 | 0.3115 | 0.3671 | 0.4093 | 0.4076 | 0.4092 | 0.3677 | 0.3199 | 0.3719 | 0.3422 |
| S26 | 0.3430 | 0.3606 | 0.2287 | 0.2293 | 0.3441 | 0.2332 | 0.3330 | 0.3235 | 0.3843 | 0.3269 | 0.2741 | 0.3332 | 0.3534 | 0.3503 | 0.3506 | 0.3352 | 0.1987 | 0.3384 | 0.3459 |
| S27 | 0.3516 | 0.3362 | 0.3286 | 0.3213 | 0.3406 | 0.3066 | 0.3286 | 0.2835 | 0.3481 | 0.2862 | 0.2710 | 0.3296 | 0.3470 | 0.3453 | 0.3465 | 0.3289 | 0.3031 | 0.3300 | 0.3535 |
| S28 | 0.3550 | 0.3103 | 0.2659 | 0.2607 | 0.2827 | 0.2438 | 0.2605 | 0.2079 | 0.3586 | 0.2091 | 0.2090 | 0.2663 | 0.3060 | 0.3046 | 0.3075 | 0.2638 | 0.2368 | 0.2669 | 0.3548 |
| S29 | 0.4131 | 0.3734 | 0.3246 | 0.3201 | 0.2099 | 0.3034 | 0.1959 | 0.3087 | 0.4071 | 0.3110 | 0.2723 | 0.1869 | 0.2340 | 0.2350 | 0.2353 | 0.1773 | 0.2968 | 0.1857 | 0.4131 |
| S3 | 0.3573 | 0.3416 | 0.2048 | 0.2013 | 0.3253 | 0.2128 | 0.3096 | 0.3014 | 0.3838 | 0.3012 | 0.2547 | 0.3107 | 0.3438 | 0.3448 | 0.3438 | 0.3124 | 0.1323 | 0.3183 | 0.3575 |
| S30 | 0.3927 | 0.4005 | 0.4224 | 0.4215 | 0.3529 | 0.3964 | 0.3537 | 0.4195 | 0.3679 | 0.4203 | 0.3734 | 0.3524 | 0.3308 | 0.3321 | 0.3286 | 0.3502 | 0.4011 | 0.3471 | 0.3917 |
| S31 | 0.4157 | 0.3758 | 0.3318 | 0.3273 | 0.2093 | 0.3096 | 0.1957 | 0.3159 | 0.4108 | 0.3140 | 0.2746 | 0.1828 | 0.2299 | 0.2296 | 0.2270 | 0.1773 | 0.3018 | 0.1806 | 0.4145 |
| S32 | 0.3467 | 0.1857 | 0.3452 | 0.3401 | 0.3823 | 0.3291 | 0.3644 | 0.3295 | 0.2672 | 0.3254 | 0.3055 | 0.3627 | 0.4047 | 0.4034 | 0.4026 | 0.3620 | 0.3168 | 0.3657 | 0.3433 |
| S33 | 0.4179 | 0.3790 | 0.3343 | 0.3302 | 0.2080 | 0.3151 | 0.1932 | 0.3178 | 0.4152 | 0.3156 | 0.2790 | 0.1774 | 0.2306 | 0.2268 | 0.2303 | 0.1726 | 0.3029 | 0.1746 | 0.4183 |
| S34 | 0.3199 | 0.2455 | 0.3301 | 0.3278 | 0.3716 | 0.3184 | 0.3502 | 0.3205 | 0.3024 | 0.3223 | 0.2973 | 0.3537 | 0.3939 | 0.3945 | 0.3934 | 0.3571 | 0.3064 | 0.3601 | 0.3159 |
| S35 | 0.4178 | 0.3693 | 0.3913 | 0.3884 | 0.2793 | 0.3652 | 0.2783 | 0.3805 | 0.3926 | 0.3790 | 0.3375 | 0.2645 | 0.2874 | 0.2864 | 0.2824 | 0.2582 | 0.3611 | 0.2579 | 0.4190 |
| S36 | 0.3697 | 0.3138 | 0.2952 | 0.2917 | 0.3159 | 0.2731 | 0.2955 | 0.1921 | 0.3695 | 0.1918 | 0.2351 | 0.2968 | 0.3414 | 0.3405 | 0.3412 | 0.2944 | 0.2657 | 0.2966 | 0.3701 |
| S37 | 0.3830 | 0.3408 | 0.2905 | 0.2854 | 0.3082 | 0.2709 | 0.2873 | 0.2870 | 0.3853 | 0.2875 | 0.2501 | 0.2886 | 0.3158 | 0.3189 | 0.3174 | 0.2867 | 0.2654 | 0.2915 | 0.3814 |
| S38 | 0.3733 | 0.3669 | 0.4254 | 0.4211 | 0.3858 | 0.4015 | 0.3902 | 0.4234 | 0.3387 | 0.4260 | 0.3820 | 0.3844 | 0.3740 | 0.3737 | 0.3723 | 0.3831 | 0.4039 | 0.3821 | 0.3776 |
| S39 | 0.3870 | 0.3470 | 0.2950 | 0.2887 | 0.3090 | 0.2753 | 0.2904 | 0.2950 | 0.3921 | 0.2914 | 0.2524 | 0.2923 | 0.3189 | 0.3236 | 0.3230 | 0.2904 | 0.2698 | 0.2971 | 0.3856 |
| S4 | 0.3580 | 0.3215 | 0.2187 | 0.2137 | 0.3102 | 0.1607 | 0.2914 | 0.2736 | 0.3756 | 0.2769 | 0.2383 | 0.2909 | 0.3301 | 0.3301 | 0.3321 | 0.2903 | 0.1966 | 0.2959 | 0.3580 |
| S40 | 0.2887 | 0.2601 | 0.3238 | 0.3213 | 0.3620 | 0.3087 | 0.3431 | 0.3134 | 0.3176 | 0.3124 | 0.2863 | 0.3441 | 0.3823 | 0.3797 | 0.3832 | 0.3444 | 0.2940 | 0.3479 | 0.2860 |
| S41 | 0.2905 | 0.2587 | 0.3245 | 0.3199 | 0.3656 | 0.3085 | 0.3448 | 0.3137 | 0.3181 | 0.3130 | 0.2857 | 0.3478 | 0.3833 | 0.3844 | 0.3847 | 0.3449 | 0.2965 | 0.3515 | 0.2809 |
| S42 | 0.3939 | 0.3443 | 0.3200 | 0.3122 | 0.3353 | 0.2998 | 0.3186 | 0.0885 | 0.3949 | 0.0904 | 0.2556 | 0.3221 | 0.3578 | 0.3590 | 0.3594 | 0.3154 | 0.2910 | 0.3212 | 0.3923 |
| S44 | 0.3320 | 0.2678 | 0.3971 | 0.3959 | 0.4276 | 0.3773 | 0.4152 | 0.3764 | 0.2617 | 0.3763 | 0.3579 | 0.4160 | 0.4342 | 0.4333 | 0.4348 | 0.4173 | 0.3763 | 0.4187 | 0.3340 |
| S45 | 0.3418 | 0.1891 | 0.3582 | 0.3515 | 0.3955 | 0.3358 | 0.3782 | 0.3399 | 0.2765 | 0.3382 | 0.3184 | 0.3759 | 0.4113 | 0.4116 | 0.4136 | 0.3742 | 0.3286 | 0.3758 | 0.3388 |
| S46 | 0.3442 | 0.1872 | 0.3503 | 0.3449 | 0.3896 | 0.3335 | 0.3716 | 0.3352 | 0.2851 | 0.3386 | 0.3144 | 0.3713 | 0.4089 | 0.4087 | 0.4105 | 0.3713 | 0.3260 | 0.3739 | 0.3404 |
| S47 | 0.3330 | 0.2629 | 0.4040 | 0.4021 | 0.4355 | 0.3869 | 0.4251 | 0.3893 | 0.2539 | 0.3900 | 0.3643 | 0.4252 | 0.4418 | 0.4434 | 0.4421 | 0.4247 | 0.3826 | 0.4272 | 0.3344 |
| S48 | 0.2098 | 0.3512 | 0.3793 | 0.3771 | 0.4230 | 0.3610 | 0.4162 | 0.3941 | 0.3256 | 0.3950 | 0.3421 | 0.4150 | 0.4258 | 0.4231 | 0.4234 | 0.4164 | 0.3609 | 0.4200 | 0.2058 |
| S49 | 0.3838 | 0.3597 | 0.3106 | 0.3073 | 0.3199 | 0.2888 | 0.3044 | 0.3130 | 0.3933 | 0.3092 | 0.2663 | 0.3065 | 0.3203 | 0.3201 | 0.3221 | 0.3060 | 0.2861 | 0.3077 | 0.3846 |
| S5 | 0.3899 | 0.3501 | 0.2962 | 0.2939 | 0.3134 | 0.2767 | 0.2938 | 0.2960 | 0.3909 | 0.2943 | 0.2544 | 0.2915 | 0.3204 | 0.3245 | 0.3229 | 0.2936 | 0.2686 | 0.2987 | 0.3857 |
| S50 | 0.3833 | 0.3397 | 0.2951 | 0.2909 | 0.3077 | 0.2765 | 0.2896 | 0.2904 | 0.3851 | 0.2867 | 0.2521 | 0.2892 | 0.3167 | 0.3160 | 0.3172 | 0.2882 | 0.2665 | 0.2911 | 0.3825 |
| S51 | 0.3537 | 0.1553 | 0.3559 | 0.3501 | 0.3933 | 0.3400 | 0.3747 | 0.3360 | 0.2780 | 0.3369 | 0.3159 | 0.3705 | 0.4102 | 0.4100 | 0.4107 | 0.3696 | 0.3260 | 0.3726 | 0.3490 |
| S52 | 0.3229 | 0.2398 | 0.3227 | 0.3192 | 0.3638 | 0.3078 | 0.3450 | 0.3140 | 0.3071 | 0.3162 | 0.2904 | 0.3466 | 0.3901 | 0.3891 | 0.3898 | 0.3478 | 0.2986 | 0.3537 | 0.3187 |
| S53 | 0.3321 | 0.2562 | 0.3944 | 0.3971 | 0.4362 | 0.3823 | 0.4233 | 0.3973 | 0.2570 | 0.3956 | 0.3644 | 0.4239 | 0.4421 | 0.4411 | 0.4411 | 0.4252 | 0.3785 | 0.4265 | 0.3330 |
| S54 | 0.3368 | 0.2477 | 0.3988 | 0.3997 | 0.4369 | 0.3816 | 0.4240 | 0.3971 | 0.2543 | 0.3979 | 0.3663 | 0.4250 | 0.4438 | 0.4434 | 0.4454 | 0.4286 | 0.3803 | 0.4291 | 0.3378 |
| S55 | 0.3252 | 0.2355 | 0.3331 | 0.3273 | 0.3731 | 0.3198 | 0.3517 | 0.3217 | 0.3017 | 0.3249 | 0.3035 | 0.3558 | 0.3960 | 0.3954 | 0.3955 | 0.3560 | 0.3046 | 0.3613 | 0.3231 |
| S56 | 0.3348 | 0.2639 | 0.4016 | 0.4013 | 0.4341 | 0.3863 | 0.4221 | 0.3878 | 0.2547 | 0.3899 | 0.3637 | 0.4219 | 0.4393 | 0.4395 | 0.4406 | 0.4229 | 0.3829 | 0.4260 | 0.3309 |
| S57 | 0.3863 | 0.3356 | 0.3107 | 0.3027 | 0.3264 | 0.2895 | 0.3065 | 0.1283 | 0.3870 | 0.1294 | 0.2500 | 0.3093 | 0.3488 | 0.3512 | 0.3498 | 0.3060 | 0.2782 | 0.3069 | 0.3836 |
| S58 | 0.3645 | 0.3211 | 0.2869 | 0.2798 | 0.3171 | 0.2699 | 0.2984 | 0.2063 | 0.3685 | 0.2116 | 0.2385 | 0.3009 | 0.3397 | 0.3389 | 0.3399 | 0.3006 | 0.2627 | 0.3024 | 0.3623 |
| S59 | 0.3657 | 0.3320 | 0.3058 | 0.3039 | 0.3274 | 0.2852 | 0.3109 | 0.2090 | 0.3709 | 0.2122 | 0.2477 | 0.3137 | 0.3465 | 0.3459 | 0.3450 | 0.3100 | 0.2816 | 0.3120 | 0.3672 |
| S6 | 0.3558 | 0.3201 | 0.2203 | 0.2146 | 0.3110 | 0.1642 | 0.2912 | 0.2732 | 0.3720 | 0.2758 | 0.2375 | 0.2898 | 0.3309 | 0.3287 | 0.3292 | 0.2890 | 0.1998 | 0.2970 | 0.3559 |
| S60 | 0.3310 | 0.2580 | 0.3936 | 0.3916 | 0.4282 | 0.3776 | 0.4212 | 0.3923 | 0.2551 | 0.3920 | 0.3600 | 0.4188 | 0.4409 | 0.4378 | 0.4384 | 0.4217 | 0.3748 | 0.4224 | 0.3296 |
| S61 | 0.3482 | 0.3126 | 0.2709 | 0.2647 | 0.2841 | 0.2504 | 0.2661 | 0.2402 | 0.3609 | 0.2416 | 0.1312 | 0.2700 | 0.3093 | 0.3092 | 0.3075 | 0.2644 | 0.2427 | 0.2724 | 0.3457 |
| S62 | 0.3501 | 0.3097 | 0.2698 | 0.2619 | 0.2809 | 0.2488 | 0.2608 | 0.2378 | 0.3582 | 0.2377 | 0.1419 | 0.2644 | 0.3082 | 0.3079 | 0.3096 | 0.2636 | 0.2374 | 0.2680 | 0.3504 |
| S63 | 0.3766 | 0.3381 | 0.1633 | 0.1597 | 0.3333 | 0.2142 | 0.3165 | 0.3027 | 0.3911 | 0.3033 | 0.2661 | 0.3174 | 0.3558 | 0.3533 | 0.3545 | 0.3187 | 0.1908 | 0.3221 | 0.3735 |
| S65 | 0.3928 | 0.3425 | 0.3235 | 0.3150 | 0.3362 | 0.3006 | 0.3192 | 0.0891 | 0.3930 | 0.0815 | 0.2605 | 0.3240 | 0.3594 | 0.3611 | 0.3608 | 0.3152 | 0.2914 | 0.3234 | 0.3932 |
| S7 | 0.4156 | 0.3804 | 0.3321 | 0.3256 | 0.1822 | 0.3090 | 0.1772 | 0.3158 | 0.4149 | 0.3158 | 0.2778 | 0.1860 | 0.2377 | 0.2368 | 0.2362 | 0.1976 | 0.3032 | 0.2040 | 0.4134 |
| S71 | 0.3740 | 0.3366 | 0.1622 | 0.1584 | 0.3320 | 0.2158 | 0.3135 | 0.3030 | 0.3916 | 0.3026 | 0.2641 | 0.3174 | 0.3574 | 0.3576 | 0.3555 | 0.3171 | 0.1878 | 0.3229 | 0.3742 |
| S72 | 0.3755 | 0.3372 | 0.1647 | 0.1608 | 0.3362 | 0.2171 | 0.3183 | 0.3009 | 0.3907 | 0.3014 | 0.2664 | 0.3190 | 0.3580 | 0.3595 | 0.3582 | 0.3196 | 0.1927 | 0.3244 | 0.3743 |
| S73 | 0.2190 | 0.3494 | 0.3743 | 0.3763 | 0.4205 | 0.3592 | 0.4120 | 0.3914 | 0.3305 | 0.3900 | 0.3408 | 0.4147 | 0.4223 | 0.4231 | 0.4199 | 0.4159 | 0.3579 | 0.4168 | 0.2103 |
| S74 | 0.3673 | 0.3303 | 0.3056 | 0.2998 | 0.3232 | 0.2821 | 0.3063 | 0.2059 | 0.3711 | 0.2084 | 0.2460 | 0.3094 | 0.3446 | 0.3430 | 0.3441 | 0.3081 | 0.2778 | 0.3133 | 0.3684 |
| S75 | 0.3704 | 0.3195 | 0.2957 | 0.2905 | 0.3169 | 0.2741 | 0.2954 | 0.1959 | 0.3669 | 0.1974 | 0.2365 | 0.2990 | 0.3399 | 0.3388 | 0.3384 | 0.2971 | 0.2693 | 0.2990 | 0.3686 |
| S76 | 0.4143 | 0.3723 | 0.3299 | 0.3251 | 0.1812 | 0.3086 | 0.1784 | 0.3118 | 0.4130 | 0.3130 | 0.2727 | 0.1839 | 0.2428 | 0.2404 | 0.2429 | 0.1960 | 0.2979 | 0.1955 | 0.4108 |
| S77 | 0.4151 | 0.3772 | 0.3285 | 0.3222 | 0.1665 | 0.3080 | 0.1704 | 0.3162 | 0.4120 | 0.3151 | 0.2768 | 0.1802 | 0.2384 | 0.2392 | 0.2367 | 0.1923 | 0.2970 | 0.1976 | 0.4140 |
| S78 | 0.4133 | 0.3748 | 0.3291 | 0.3239 | 0.1789 | 0.3083 | 0.1735 | 0.3163 | 0.4106 | 0.3153 | 0.2786 | 0.1842 | 0.2414 | 0.2368 | 0.2378 | 0.1932 | 0.2974 | 0.1974 | 0.4123 |
| S79 | 0.3530 | 0.1692 | 0.3519 | 0.3486 | 0.3905 | 0.3391 | 0.3747 | 0.3372 | 0.2755 | 0.3368 | 0.3172 | 0.3728 | 0.4148 | 0.4111 | 0.4152 | 0.3744 | 0.3281 | 0.3794 | 0.3488 |
| S8 | 0.4140 | 0.3744 | 0.3323 | 0.3246 | 0.1823 | 0.3104 | 0.1798 | 0.3181 | 0.4127 | 0.3175 | 0.2795 | 0.1880 | 0.2391 | 0.2364 | 0.2376 | 0.1958 | 0.3022 | 0.2007 | 0.4124 |
| S80 | 0.3370 | 0.2523 | 0.3984 | 0.4001 | 0.4348 | 0.3816 | 0.4231 | 0.3950 | 0.2578 | 0.3961 | 0.3647 | 0.4255 | 0.4432 | 0.4405 | 0.4433 | 0.4248 | 0.3793 | 0.4254 | 0.3349 |
| S84 | 0.3226 | 0.2375 | 0.3307 | 0.3243 | 0.3687 | 0.3169 | 0.3496 | 0.3194 | 0.3026 | 0.3190 | 0.2943 | 0.3517 | 0.3939 | 0.3927 | 0.3924 | 0.3535 | 0.3037 | 0.3574 | 0.3197 |
| S85 | 0.3734 | 0.3375 | 0.1379 | 0.1347 | 0.3295 | 0.2096 | 0.3091 | 0.2978 | 0.3878 | 0.2986 | 0.2581 | 0.3127 | 0.3547 | 0.3506 | 0.3540 | 0.3127 | 0.1821 | 0.3189 | 0.3685 |
| S86 | 0.3430 | 0.2016 | 0.3269 | 0.3208 | 0.3618 | 0.3102 | 0.3394 | 0.3057 | 0.2910 | 0.3068 | 0.2881 | 0.3416 | 0.3818 | 0.3839 | 0.3854 | 0.3412 | 0.3010 | 0.3442 | 0.3393 |
| S87 | 0.2821 | 0.2641 | 0.3291 | 0.3225 | 0.3655 | 0.3090 | 0.3478 | 0.3195 | 0.3185 | 0.3203 | 0.2864 | 0.3496 | 0.3838 | 0.3821 | 0.3840 | 0.3482 | 0.2993 | 0.3530 | 0.2743 |
| S88 | 0.3516 | 0.3921 | 0.2369 | 0.2376 | 0.3685 | 0.2428 | 0.3610 | 0.3563 | 0.3960 | 0.3550 | 0.3025 | 0.3626 | 0.3685 | 0.3678 | 0.3674 | 0.3624 | 0.2428 | 0.3666 | 0.3565 |
| S89 | 0.3690 | 0.3147 | 0.2946 | 0.2879 | 0.3139 | 0.2703 | 0.2945 | 0.1936 | 0.3691 | 0.1902 | 0.2351 | 0.2957 | 0.3411 | 0.3412 | 0.3427 | 0.2936 | 0.2657 | 0.2979 | 0.3687 |
| S9 | 0.3389 | 0.2482 | 0.3987 | 0.4000 | 0.4405 | 0.3847 | 0.4260 | 0.3948 | 0.2545 | 0.3979 | 0.3698 | 0.4271 | 0.4444 | 0.4416 | 0.4458 | 0.4258 | 0.3814 | 0.4289 | 0.3399 |
| S90 | 0.3785 | 0.3201 | 0.2870 | 0.2761 | 0.3091 | 0.2657 | 0.2874 | 0.2438 | 0.3769 | 0.2419 | 0.2229 | 0.2888 | 0.3334 | 0.3348 | 0.3359 | 0.2889 | 0.2584 | 0.2900 | 0.3733 |
| S91 | 0.3769 | 0.3246 | 0.2878 | 0.2777 | 0.3130 | 0.2657 | 0.2879 | 0.2493 | 0.3764 | 0.2500 | 0.2297 | 0.2937 | 0.3360 | 0.3306 | 0.3388 | 0.2915 | 0.2625 | 0.2942 | 0.3724 |
| S92 | 0.3752 | 0.3228 | 0.2895 | 0.2828 | 0.3111 | 0.2687 | 0.2874 | 0.2508 | 0.3734 | 0.2502 | 0.2275 | 0.2893 | 0.3374 | 0.3332 | 0.3376 | 0.2892 | 0.2617 | 0.2920 | 0.3717 |
| S93 | 0.3769 | 0.3228 | 0.2852 | 0.2751 | 0.3044 | 0.2641 | 0.2856 | 0.2485 | 0.3735 | 0.2503 | 0.2271 | 0.2897 | 0.3350 | 0.3333 | 0.3366 | 0.2875 | 0.2606 | 0.2898 | 0.3698 |
| S95 | 0.3776 | 0.3283 | 0.2898 | 0.2821 | 0.3164 | 0.2718 | 0.2958 | 0.2563 | 0.3776 | 0.2560 | 0.2323 | 0.3002 | 0.3395 | 0.3391 | 0.3445 | 0.2968 | 0.2671 | 0.3017 | 0.3727 |
| S96 | 0.3894 | 0.3501 | 0.2873 | 0.2812 | 0.3056 | 0.2672 | 0.2859 | 0.2955 | 0.3922 | 0.2940 | 0.2542 | 0.2981 | 0.3252 | 0.3288 | 0.3282 | 0.2952 | 0.2713 | 0.3027 | 0.3858 |
| S97 | 0.2270 | 0.3505 | 0.3537 | 0.3527 | 0.4062 | 0.3398 | 0.3961 | 0.3872 | 0.3352 | 0.3869 | 0.3408 | 0.4098 | 0.4212 | 0.4196 | 0.4200 | 0.4104 | 0.3484 | 0.4135 | 0.2259 |
| S98 | 0.3917 | 0.3484 | 0.2863 | 0.2826 | 0.3008 | 0.2658 | 0.2828 | 0.2953 | 0.3891 | 0.2927 | 0.2540 | 0.2927 | 0.3246 | 0.3261 | 0.3270 | 0.2933 | 0.2688 | 0.2969 | 0.3866 |
| S99 | 0.3876 | 0.3501 | 0.2857 | 0.2814 | 0.3042 | 0.2649 | 0.2862 | 0.2970 | 0.3951 | 0.2965 | 0.2531 | 0.2972 | 0.3242 | 0.3288 | 0.3306 | 0.2972 | 0.2701 | 0.3032 | 0.3863 |

Table S4 (Continued)

| Sample ID | S22 | S23 | S24 | S26 | S27 | S28 | S29 | S3 | S30 | S31 | S32 | S33 | S34 | S35 | S36 | S37 | S38 | S39 | S4 |
| --- | --- | --- | --- | --- | --- | --- | --- | --- | --- | --- | --- | --- | --- | --- | --- | --- | --- | --- | --- |
| S1 | 0.3325 | 0.3441 | 0.3459 | 0.3430 | 0.3516 | 0.3550 | 0.4131 | 0.3573 | 0.3927 | 0.4157 | 0.3467 | 0.4179 | 0.3199 | 0.4178 | 0.3697 | 0.3830 | 0.3733 | 0.3870 | 0.3580 |
| S10 | 0.2642 | 0.1904 | 0.1935 | 0.3606 | 0.3362 | 0.3103 | 0.3734 | 0.3416 | 0.4005 | 0.3758 | 0.1857 | 0.3790 | 0.2455 | 0.3693 | 0.3138 | 0.3408 | 0.3669 | 0.3470 | 0.3215 |
| S101 | 0.3901 | 0.3534 | 0.3480 | 0.2287 | 0.3286 | 0.2659 | 0.3246 | 0.2048 | 0.4224 | 0.3318 | 0.3452 | 0.3343 | 0.3301 | 0.3913 | 0.2952 | 0.2905 | 0.4254 | 0.2950 | 0.2187 |
| S102 | 0.3892 | 0.3490 | 0.3440 | 0.2293 | 0.3213 | 0.2607 | 0.3201 | 0.2013 | 0.4215 | 0.3273 | 0.3401 | 0.3302 | 0.3278 | 0.3884 | 0.2917 | 0.2854 | 0.4211 | 0.2887 | 0.2137 |
| S103 | 0.4230 | 0.3893 | 0.3853 | 0.3441 | 0.3406 | 0.2827 | 0.2099 | 0.3253 | 0.3529 | 0.2093 | 0.3823 | 0.2080 | 0.3716 | 0.2793 | 0.3159 | 0.3082 | 0.3858 | 0.3090 | 0.3102 |
| S104 | 0.3688 | 0.3368 | 0.3286 | 0.2332 | 0.3066 | 0.2438 | 0.3034 | 0.2128 | 0.3964 | 0.3096 | 0.3291 | 0.3151 | 0.3184 | 0.3652 | 0.2731 | 0.2709 | 0.4015 | 0.2753 | 0.1607 |
| S105 | 0.4110 | 0.3735 | 0.3682 | 0.3330 | 0.3286 | 0.2605 | 0.1959 | 0.3096 | 0.3537 | 0.1957 | 0.3644 | 0.1932 | 0.3502 | 0.2783 | 0.2955 | 0.2873 | 0.3902 | 0.2904 | 0.2914 |
| S11 | 0.3706 | 0.3387 | 0.3324 | 0.3235 | 0.2835 | 0.2079 | 0.3087 | 0.3014 | 0.4195 | 0.3159 | 0.3295 | 0.3178 | 0.3205 | 0.3805 | 0.1921 | 0.2870 | 0.4234 | 0.2950 | 0.2736 |
| S12 | 0.2613 | 0.2815 | 0.2704 | 0.3843 | 0.3481 | 0.3586 | 0.4071 | 0.3838 | 0.3679 | 0.4108 | 0.2672 | 0.4152 | 0.3024 | 0.3926 | 0.3695 | 0.3853 | 0.3387 | 0.3921 | 0.3756 |
| S13 | 0.3694 | 0.3378 | 0.3323 | 0.3269 | 0.2862 | 0.2091 | 0.3110 | 0.3012 | 0.4203 | 0.3140 | 0.3254 | 0.3156 | 0.3223 | 0.3790 | 0.1918 | 0.2875 | 0.4260 | 0.2914 | 0.2769 |
| S14 | 0.3529 | 0.3157 | 0.3115 | 0.2741 | 0.2710 | 0.2090 | 0.2723 | 0.2547 | 0.3734 | 0.2746 | 0.3055 | 0.2790 | 0.2973 | 0.3375 | 0.2351 | 0.2501 | 0.3820 | 0.2524 | 0.2383 |
| S15 | 0.4109 | 0.3747 | 0.3671 | 0.3332 | 0.3296 | 0.2663 | 0.1869 | 0.3107 | 0.3524 | 0.1828 | 0.3627 | 0.1774 | 0.3537 | 0.2645 | 0.2968 | 0.2886 | 0.3844 | 0.2923 | 0.2909 |
| S16 | 0.4278 | 0.4089 | 0.4093 | 0.3534 | 0.3470 | 0.3060 | 0.2340 | 0.3438 | 0.3308 | 0.2299 | 0.4047 | 0.2306 | 0.3939 | 0.2874 | 0.3414 | 0.3158 | 0.3740 | 0.3189 | 0.3301 |
| S17 | 0.4273 | 0.4121 | 0.4076 | 0.3503 | 0.3453 | 0.3046 | 0.2350 | 0.3448 | 0.3321 | 0.2296 | 0.4034 | 0.2268 | 0.3945 | 0.2864 | 0.3405 | 0.3189 | 0.3737 | 0.3236 | 0.3301 |
| S18 | 0.4304 | 0.4111 | 0.4092 | 0.3506 | 0.3465 | 0.3075 | 0.2353 | 0.3438 | 0.3286 | 0.2270 | 0.4026 | 0.2303 | 0.3934 | 0.2824 | 0.3412 | 0.3174 | 0.3723 | 0.3230 | 0.3321 |
| S19 | 0.4088 | 0.3719 | 0.3677 | 0.3352 | 0.3289 | 0.2638 | 0.1773 | 0.3124 | 0.3502 | 0.1773 | 0.3620 | 0.1726 | 0.3571 | 0.2582 | 0.2944 | 0.2867 | 0.3831 | 0.2904 | 0.2903 |
| S2 | 0.3674 | 0.3259 | 0.3199 | 0.1987 | 0.3031 | 0.2368 | 0.2968 | 0.1323 | 0.4011 | 0.3018 | 0.3168 | 0.3029 | 0.3064 | 0.3611 | 0.2657 | 0.2654 | 0.4039 | 0.2698 | 0.1966 |
| S20 | 0.4117 | 0.3768 | 0.3719 | 0.3384 | 0.3300 | 0.2669 | 0.1857 | 0.3183 | 0.3471 | 0.1806 | 0.3657 | 0.1746 | 0.3601 | 0.2579 | 0.2966 | 0.2915 | 0.3821 | 0.2971 | 0.2959 |
| S21 | 0.3326 | 0.3409 | 0.3422 | 0.3459 | 0.3535 | 0.3548 | 0.4131 | 0.3575 | 0.3917 | 0.4145 | 0.3433 | 0.4183 | 0.3159 | 0.4190 | 0.3701 | 0.3814 | 0.3776 | 0.3856 | 0.3580 |
| S22 | 0.0000 | 0.2626 | 0.2727 | 0.3753 | 0.3340 | 0.3406 | 0.4044 | 0.3725 | 0.3714 | 0.4079 | 0.2701 | 0.4111 | 0.3013 | 0.3939 | 0.3445 | 0.3778 | 0.3359 | 0.3850 | 0.3570 |
| S23 | 0.2626 | 0.0000 | 0.2074 | 0.3558 | 0.3330 | 0.3046 | 0.3687 | 0.3368 | 0.3963 | 0.3710 | 0.2007 | 0.3743 | 0.2440 | 0.3721 | 0.3067 | 0.3368 | 0.3668 | 0.3448 | 0.3165 |
| S24 | 0.2727 | 0.2074 | 0.0000 | 0.3508 | 0.3334 | 0.2982 | 0.3639 | 0.3330 | 0.4012 | 0.3687 | 0.1611 | 0.3698 | 0.2263 | 0.3733 | 0.3043 | 0.3299 | 0.3744 | 0.3378 | 0.3075 |
| S26 | 0.3753 | 0.3558 | 0.3508 | 0.0000 | 0.3173 | 0.2776 | 0.3279 | 0.1943 | 0.3970 | 0.3347 | 0.3500 | 0.3368 | 0.3405 | 0.3790 | 0.3003 | 0.2982 | 0.3989 | 0.3012 | 0.2321 |
| S27 | 0.3340 | 0.3330 | 0.3334 | 0.3173 | 0.0000 | 0.2092 | 0.3225 | 0.3111 | 0.3502 | 0.3277 | 0.3309 | 0.3320 | 0.3315 | 0.3562 | 0.2716 | 0.3074 | 0.3463 | 0.3155 | 0.2946 |
| S28 | 0.3406 | 0.3046 | 0.2982 | 0.2776 | 0.2092 | 0.0000 | 0.2540 | 0.2512 | 0.3706 | 0.2583 | 0.2938 | 0.2612 | 0.2892 | 0.3331 | 0.1953 | 0.2379 | 0.3803 | 0.2440 | 0.2238 |
| S29 | 0.4044 | 0.3687 | 0.3639 | 0.3279 | 0.3225 | 0.2540 | 0.0000 | 0.3071 | 0.3372 | 0.1677 | 0.3580 | 0.1707 | 0.3520 | 0.2653 | 0.2888 | 0.2806 | 0.3750 | 0.2845 | 0.2838 |
| S3 | 0.3725 | 0.3368 | 0.3330 | 0.1943 | 0.3111 | 0.2512 | 0.3071 | 0.0000 | 0.4015 | 0.3107 | 0.3282 | 0.3162 | 0.3186 | 0.3682 | 0.2782 | 0.2728 | 0.4056 | 0.2768 | 0.2059 |
| S30 | 0.3714 | 0.3963 | 0.4012 | 0.3970 | 0.3502 | 0.3706 | 0.3372 | 0.4015 | 0.0000 | 0.3396 | 0.3991 | 0.3449 | 0.4026 | 0.3279 | 0.3992 | 0.3944 | 0.2735 | 0.3972 | 0.3932 |
| S31 | 0.4079 | 0.3710 | 0.3687 | 0.3347 | 0.3277 | 0.2583 | 0.1677 | 0.3107 | 0.3396 | 0.0000 | 0.3638 | 0.1681 | 0.3547 | 0.2589 | 0.2898 | 0.2853 | 0.3749 | 0.2892 | 0.2912 |
| S32 | 0.2701 | 0.2007 | 0.1611 | 0.3500 | 0.3309 | 0.2938 | 0.3580 | 0.3282 | 0.3991 | 0.3638 | 0.0000 | 0.3650 | 0.2273 | 0.3674 | 0.2988 | 0.3251 | 0.3715 | 0.3326 | 0.3053 |
| S33 | 0.4111 | 0.3743 | 0.3698 | 0.3368 | 0.3320 | 0.2612 | 0.1707 | 0.3162 | 0.3449 | 0.1681 | 0.3650 | 0.0000 | 0.3581 | 0.2611 | 0.2940 | 0.2878 | 0.3811 | 0.2910 | 0.2924 |
| S34 | 0.3013 | 0.2440 | 0.2263 | 0.3405 | 0.3315 | 0.2892 | 0.3520 | 0.3186 | 0.4026 | 0.3547 | 0.2273 | 0.3581 | 0.0000 | 0.3813 | 0.2930 | 0.3177 | 0.3800 | 0.3217 | 0.3014 |
| S35 | 0.3939 | 0.3721 | 0.3733 | 0.3790 | 0.3562 | 0.3331 | 0.2653 | 0.3682 | 0.3279 | 0.2589 | 0.3674 | 0.2611 | 0.3813 | 0.0000 | 0.3604 | 0.3564 | 0.3454 | 0.3635 | 0.3550 |
| S36 | 0.3445 | 0.3067 | 0.3043 | 0.3003 | 0.2716 | 0.1953 | 0.2888 | 0.2782 | 0.3992 | 0.2898 | 0.2988 | 0.2940 | 0.2930 | 0.3604 | 0.0000 | 0.2621 | 0.4031 | 0.2699 | 0.2529 |
| S37 | 0.3778 | 0.3368 | 0.3299 | 0.2982 | 0.3074 | 0.2379 | 0.2806 | 0.2728 | 0.3944 | 0.2853 | 0.3251 | 0.2878 | 0.3177 | 0.3564 | 0.2621 | 0.0000 | 0.4022 | 0.1535 | 0.2524 |
| S38 | 0.3359 | 0.3668 | 0.3744 | 0.3989 | 0.3463 | 0.3803 | 0.3750 | 0.4056 | 0.2735 | 0.3749 | 0.3715 | 0.3811 | 0.3800 | 0.3454 | 0.4031 | 0.4022 | 0.0000 | 0.4079 | 0.3985 |
| S39 | 0.3850 | 0.3448 | 0.3378 | 0.3012 | 0.3155 | 0.2440 | 0.2845 | 0.2768 | 0.3972 | 0.2892 | 0.3326 | 0.2910 | 0.3217 | 0.3635 | 0.2699 | 0.1535 | 0.4079 | 0.0000 | 0.2567 |
| S4 | 0.3570 | 0.3165 | 0.3075 | 0.2321 | 0.2946 | 0.2238 | 0.2838 | 0.2059 | 0.3932 | 0.2912 | 0.3053 | 0.2924 | 0.3014 | 0.3550 | 0.2529 | 0.2524 | 0.3985 | 0.2567 | 0.0000 |
| S40 | 0.3097 | 0.2547 | 0.2477 | 0.3291 | 0.3250 | 0.2787 | 0.3402 | 0.3069 | 0.3984 | 0.3444 | 0.2471 | 0.3458 | 0.2141 | 0.3772 | 0.2873 | 0.3085 | 0.3831 | 0.3129 | 0.2910 |
| S41 | 0.3063 | 0.2546 | 0.2473 | 0.3283 | 0.3209 | 0.2790 | 0.3425 | 0.3104 | 0.4031 | 0.3477 | 0.2429 | 0.3467 | 0.2127 | 0.3777 | 0.2870 | 0.3064 | 0.3831 | 0.3126 | 0.2901 |
| S42 | 0.3712 | 0.3374 | 0.3307 | 0.3260 | 0.2867 | 0.2093 | 0.3108 | 0.3021 | 0.4196 | 0.3135 | 0.3282 | 0.3169 | 0.3220 | 0.3805 | 0.1892 | 0.2875 | 0.4246 | 0.2917 | 0.2756 |
| S44 | 0.2203 | 0.2670 | 0.2739 | 0.3824 | 0.3413 | 0.3496 | 0.4120 | 0.3797 | 0.3735 | 0.4145 | 0.2769 | 0.4173 | 0.3052 | 0.3950 | 0.3526 | 0.3863 | 0.3366 | 0.3919 | 0.3689 |
| S45 | 0.2591 | 0.1642 | 0.2098 | 0.3584 | 0.3329 | 0.3046 | 0.3699 | 0.3392 | 0.3992 | 0.3740 | 0.2010 | 0.3763 | 0.2457 | 0.3719 | 0.3087 | 0.3366 | 0.3656 | 0.3458 | 0.3170 |
| S46 | 0.2649 | 0.1641 | 0.2064 | 0.3547 | 0.3323 | 0.3034 | 0.3666 | 0.3381 | 0.3982 | 0.3695 | 0.2005 | 0.3733 | 0.2462 | 0.3727 | 0.3048 | 0.3374 | 0.3686 | 0.3425 | 0.3149 |
| S47 | 0.2362 | 0.2613 | 0.2816 | 0.3861 | 0.3500 | 0.3610 | 0.4163 | 0.3874 | 0.3807 | 0.4225 | 0.2788 | 0.4286 | 0.3050 | 0.3953 | 0.3662 | 0.3921 | 0.3395 | 0.4002 | 0.3763 |
| S48 | 0.3302 | 0.3453 | 0.3432 | 0.3428 | 0.3478 | 0.3538 | 0.4116 | 0.3576 | 0.3914 | 0.4155 | 0.3468 | 0.4189 | 0.3166 | 0.4166 | 0.3716 | 0.3808 | 0.3766 | 0.3843 | 0.3572 |
| S49 | 0.3870 | 0.3556 | 0.3527 | 0.3089 | 0.3190 | 0.2637 | 0.2986 | 0.2906 | 0.3900 | 0.3015 | 0.3468 | 0.3056 | 0.3370 | 0.3632 | 0.2895 | 0.1637 | 0.4009 | 0.1744 | 0.2748 |
| S5 | 0.3850 | 0.3464 | 0.3416 | 0.2991 | 0.3175 | 0.2425 | 0.2868 | 0.2766 | 0.4001 | 0.2874 | 0.3363 | 0.2938 | 0.3257 | 0.3637 | 0.2698 | 0.1510 | 0.4079 | 0.1331 | 0.2591 |
| S50 | 0.3793 | 0.3382 | 0.3319 | 0.2990 | 0.3090 | 0.2409 | 0.2793 | 0.2730 | 0.3948 | 0.2862 | 0.3271 | 0.2895 | 0.3180 | 0.3576 | 0.2646 | 0.1458 | 0.4034 | 0.1580 | 0.2561 |
| S51 | 0.2595 | 0.1880 | 0.1933 | 0.3547 | 0.3341 | 0.3024 | 0.3660 | 0.3372 | 0.3968 | 0.3671 | 0.1863 | 0.3740 | 0.2382 | 0.3641 | 0.3070 | 0.3350 | 0.3671 | 0.3434 | 0.3188 |
| S52 | 0.2977 | 0.2410 | 0.2264 | 0.3300 | 0.3244 | 0.2799 | 0.3428 | 0.3089 | 0.4001 | 0.3468 | 0.2259 | 0.3496 | 0.1602 | 0.3772 | 0.2831 | 0.3056 | 0.3829 | 0.3110 | 0.2893 |
| S53 | 0.2601 | 0.2595 | 0.2499 | 0.3847 | 0.3543 | 0.3623 | 0.4199 | 0.3860 | 0.3908 | 0.4216 | 0.2554 | 0.4264 | 0.2892 | 0.4019 | 0.3673 | 0.3910 | 0.3560 | 0.3984 | 0.3718 |
| S54 | 0.2515 | 0.2539 | 0.2535 | 0.3910 | 0.3523 | 0.3601 | 0.4202 | 0.3890 | 0.3902 | 0.4256 | 0.2584 | 0.4285 | 0.2970 | 0.4049 | 0.3693 | 0.3932 | 0.3543 | 0.4000 | 0.3730 |
| S55 | 0.3001 | 0.2419 | 0.2187 | 0.3405 | 0.3284 | 0.2896 | 0.3548 | 0.3179 | 0.4072 | 0.3551 | 0.2218 | 0.3596 | 0.1481 | 0.3814 | 0.2967 | 0.3159 | 0.3856 | 0.3234 | 0.2992 |
| S56 | 0.2352 | 0.2614 | 0.2752 | 0.3852 | 0.3462 | 0.3585 | 0.4169 | 0.3876 | 0.3840 | 0.4212 | 0.2755 | 0.4227 | 0.3059 | 0.3957 | 0.3652 | 0.3894 | 0.3432 | 0.4003 | 0.3778 |
| S57 | 0.3642 | 0.3291 | 0.3246 | 0.3116 | 0.2745 | 0.1981 | 0.2983 | 0.2901 | 0.4139 | 0.3049 | 0.3180 | 0.3033 | 0.3138 | 0.3710 | 0.1898 | 0.2787 | 0.4181 | 0.2836 | 0.2646 |
| S58 | 0.3493 | 0.3130 | 0.3094 | 0.2984 | 0.2661 | 0.1958 | 0.2939 | 0.2749 | 0.3964 | 0.2970 | 0.3064 | 0.3009 | 0.2964 | 0.3604 | 0.1898 | 0.2713 | 0.3997 | 0.2795 | 0.2526 |
| S59 | 0.3506 | 0.3223 | 0.3225 | 0.3093 | 0.2644 | 0.2097 | 0.3038 | 0.2908 | 0.3950 | 0.3086 | 0.3181 | 0.3154 | 0.3109 | 0.3657 | 0.1966 | 0.2830 | 0.3984 | 0.2929 | 0.2679 |
| S6 | 0.3587 | 0.3175 | 0.3082 | 0.2320 | 0.2942 | 0.2242 | 0.2844 | 0.2054 | 0.3916 | 0.2910 | 0.3063 | 0.2920 | 0.2970 | 0.3526 | 0.2520 | 0.2545 | 0.3969 | 0.2575 | 0.1187 |
| S60 | 0.2596 | 0.2631 | 0.2537 | 0.3806 | 0.3506 | 0.3552 | 0.4146 | 0.3805 | 0.3895 | 0.4180 | 0.2583 | 0.4219 | 0.2891 | 0.3964 | 0.3671 | 0.3869 | 0.3555 | 0.3929 | 0.3684 |
| S61 | 0.3476 | 0.3041 | 0.2995 | 0.2731 | 0.2688 | 0.1973 | 0.2609 | 0.2515 | 0.3756 | 0.2642 | 0.2953 | 0.2688 | 0.2885 | 0.3327 | 0.2224 | 0.2380 | 0.3818 | 0.2417 | 0.2306 |
| S62 | 0.3482 | 0.3034 | 0.2960 | 0.2733 | 0.2693 | 0.1931 | 0.2581 | 0.2497 | 0.3772 | 0.2629 | 0.2903 | 0.2638 | 0.2832 | 0.3317 | 0.2151 | 0.2325 | 0.3855 | 0.2359 | 0.2245 |
| S63 | 0.3801 | 0.3355 | 0.3251 | 0.2308 | 0.3211 | 0.2534 | 0.3099 | 0.1932 | 0.4175 | 0.3161 | 0.3231 | 0.3180 | 0.3124 | 0.3785 | 0.2768 | 0.2737 | 0.4222 | 0.2810 | 0.2028 |
| S65 | 0.3688 | 0.3377 | 0.3333 | 0.3286 | 0.2859 | 0.2110 | 0.3121 | 0.3042 | 0.4167 | 0.3175 | 0.3309 | 0.3183 | 0.3229 | 0.3800 | 0.1953 | 0.2918 | 0.4230 | 0.2964 | 0.2765 |
| S7 | 0.4105 | 0.3724 | 0.3690 | 0.3357 | 0.3293 | 0.2664 | 0.2014 | 0.3115 | 0.3583 | 0.2000 | 0.3615 | 0.1985 | 0.3537 | 0.2836 | 0.2944 | 0.2869 | 0.3896 | 0.2888 | 0.2912 |
| S71 | 0.3795 | 0.3381 | 0.3250 | 0.2270 | 0.3216 | 0.2523 | 0.3104 | 0.1972 | 0.4163 | 0.3154 | 0.3253 | 0.3197 | 0.3136 | 0.3768 | 0.2745 | 0.2738 | 0.4198 | 0.2802 | 0.2034 |
| S72 | 0.3779 | 0.3338 | 0.3268 | 0.2309 | 0.3201 | 0.2550 | 0.3128 | 0.1971 | 0.4185 | 0.3173 | 0.3239 | 0.3203 | 0.3106 | 0.3809 | 0.2772 | 0.2735 | 0.4199 | 0.2782 | 0.2030 |
| S73 | 0.3316 | 0.3403 | 0.3416 | 0.3422 | 0.3476 | 0.3517 | 0.4086 | 0.3536 | 0.3901 | 0.4129 | 0.3422 | 0.4162 | 0.3140 | 0.4128 | 0.3656 | 0.3795 | 0.3746 | 0.3830 | 0.3555 |
| S74 | 0.3536 | 0.3220 | 0.3184 | 0.3082 | 0.2658 | 0.2042 | 0.3021 | 0.2893 | 0.3969 | 0.3036 | 0.3156 | 0.3104 | 0.3094 | 0.3636 | 0.1974 | 0.2795 | 0.4019 | 0.2861 | 0.2654 |
| S75 | 0.3498 | 0.3137 | 0.3106 | 0.3017 | 0.2646 | 0.1927 | 0.2902 | 0.2813 | 0.4000 | 0.2957 | 0.3023 | 0.3009 | 0.2989 | 0.3620 | 0.1863 | 0.2693 | 0.4032 | 0.2769 | 0.2524 |
| S76 | 0.4051 | 0.3684 | 0.3641 | 0.3303 | 0.3311 | 0.2628 | 0.1976 | 0.3083 | 0.3596 | 0.1987 | 0.3564 | 0.1976 | 0.3488 | 0.2819 | 0.2884 | 0.2837 | 0.3892 | 0.2867 | 0.2872 |
| S77 | 0.4114 | 0.3713 | 0.3657 | 0.3321 | 0.3296 | 0.2631 | 0.1962 | 0.3111 | 0.3547 | 0.1959 | 0.3627 | 0.1960 | 0.3522 | 0.2825 | 0.2951 | 0.2859 | 0.3902 | 0.2881 | 0.2889 |
| S78 | 0.4066 | 0.3694 | 0.3650 | 0.3321 | 0.3307 | 0.2631 | 0.1952 | 0.3099 | 0.3532 | 0.1984 | 0.3584 | 0.1952 | 0.3495 | 0.2820 | 0.2922 | 0.2841 | 0.3879 | 0.2885 | 0.2903 |
| S79 | 0.2647 | 0.1908 | 0.1905 | 0.3599 | 0.3345 | 0.3032 | 0.3684 | 0.3408 | 0.4016 | 0.3738 | 0.1917 | 0.3747 | 0.2415 | 0.3698 | 0.3071 | 0.3358 | 0.3709 | 0.3451 | 0.3172 |
| S8 | 0.4097 | 0.3729 | 0.3671 | 0.3381 | 0.3317 | 0.2660 | 0.1997 | 0.3116 | 0.3562 | 0.1989 | 0.3616 | 0.1967 | 0.3558 | 0.2837 | 0.2986 | 0.2866 | 0.3921 | 0.2920 | 0.2897 |
| S80 | 0.2584 | 0.2577 | 0.2520 | 0.3884 | 0.3504 | 0.3581 | 0.4190 | 0.3874 | 0.3911 | 0.4230 | 0.2581 | 0.4239 | 0.2935 | 0.4022 | 0.3661 | 0.3905 | 0.3561 | 0.3976 | 0.3729 |
| S84 | 0.2986 | 0.2405 | 0.2185 | 0.3389 | 0.3271 | 0.2845 | 0.3508 | 0.3169 | 0.4064 | 0.3518 | 0.2219 | 0.3566 | 0.1371 | 0.3785 | 0.2927 | 0.3127 | 0.3846 | 0.3210 | 0.2970 |
| S85 | 0.3769 | 0.3343 | 0.3258 | 0.2232 | 0.3149 | 0.2448 | 0.3068 | 0.1906 | 0.4156 | 0.3137 | 0.3232 | 0.3126 | 0.3123 | 0.3785 | 0.2718 | 0.2666 | 0.4173 | 0.2724 | 0.1999 |
| S86 | 0.2662 | 0.2091 | 0.2069 | 0.3357 | 0.3110 | 0.2686 | 0.3342 | 0.3129 | 0.3738 | 0.3387 | 0.1984 | 0.3395 | 0.2317 | 0.3632 | 0.2773 | 0.3015 | 0.3429 | 0.3086 | 0.2907 |
| S87 | 0.3115 | 0.2598 | 0.2527 | 0.3279 | 0.3208 | 0.2811 | 0.3437 | 0.3114 | 0.4002 | 0.3481 | 0.2539 | 0.3502 | 0.2235 | 0.3793 | 0.2917 | 0.3123 | 0.3826 | 0.3147 | 0.2936 |
| S88 | 0.3920 | 0.3866 | 0.3816 | 0.2272 | 0.3290 | 0.3022 | 0.3579 | 0.2384 | 0.4015 | 0.3612 | 0.3829 | 0.3628 | 0.3673 | 0.3937 | 0.3345 | 0.3234 | 0.4084 | 0.3263 | 0.2545 |
| S89 | 0.3450 | 0.3071 | 0.3035 | 0.3009 | 0.2727 | 0.1947 | 0.2871 | 0.2784 | 0.4007 | 0.2902 | 0.2979 | 0.2957 | 0.2937 | 0.3593 | 0.0560 | 0.2632 | 0.4044 | 0.2675 | 0.2500 |
| S9 | 0.2555 | 0.2530 | 0.2557 | 0.3862 | 0.3562 | 0.3657 | 0.4207 | 0.3884 | 0.3937 | 0.4243 | 0.2580 | 0.4290 | 0.2962 | 0.4025 | 0.3683 | 0.3931 | 0.3546 | 0.4001 | 0.3746 |
| S90 | 0.3622 | 0.3146 | 0.3052 | 0.2932 | 0.2829 | 0.1964 | 0.2823 | 0.2703 | 0.4068 | 0.2865 | 0.3033 | 0.2907 | 0.2937 | 0.3630 | 0.2186 | 0.2498 | 0.4128 | 0.2568 | 0.2449 |
| S91 | 0.3592 | 0.3234 | 0.3130 | 0.2985 | 0.2848 | 0.1987 | 0.2848 | 0.2762 | 0.4049 | 0.2897 | 0.3085 | 0.2926 | 0.3015 | 0.3660 | 0.2288 | 0.2548 | 0.4105 | 0.2635 | 0.2463 |
| S92 | 0.3601 | 0.3214 | 0.3085 | 0.2964 | 0.2859 | 0.2003 | 0.2845 | 0.2754 | 0.4039 | 0.2875 | 0.3054 | 0.2903 | 0.2997 | 0.3630 | 0.2259 | 0.2567 | 0.4062 | 0.2652 | 0.2475 |
| S93 | 0.3591 | 0.3192 | 0.3114 | 0.2951 | 0.2821 | 0.2002 | 0.2803 | 0.2721 | 0.4009 | 0.2864 | 0.3070 | 0.2910 | 0.3002 | 0.3606 | 0.2241 | 0.2523 | 0.4066 | 0.2555 | 0.2447 |
| S95 | 0.3632 | 0.3231 | 0.3147 | 0.3016 | 0.2897 | 0.2050 | 0.2885 | 0.2786 | 0.4050 | 0.2938 | 0.3128 | 0.2979 | 0.3041 | 0.3695 | 0.2304 | 0.2597 | 0.4115 | 0.2672 | 0.2510 |
| S96 | 0.3856 | 0.3490 | 0.3400 | 0.3032 | 0.3156 | 0.2423 | 0.2903 | 0.2794 | 0.4038 | 0.2973 | 0.3367 | 0.2966 | 0.3273 | 0.3686 | 0.2735 | 0.1599 | 0.4103 | 0.1364 | 0.2607 |
| S97 | 0.3342 | 0.3427 | 0.3448 | 0.3347 | 0.3528 | 0.3454 | 0.4054 | 0.3461 | 0.3951 | 0.4099 | 0.3436 | 0.4136 | 0.3171 | 0.4147 | 0.3636 | 0.3752 | 0.3773 | 0.3765 | 0.3479 |
| S98 | 0.3847 | 0.3445 | 0.3382 | 0.3012 | 0.3149 | 0.2416 | 0.2848 | 0.2779 | 0.4030 | 0.2892 | 0.3345 | 0.2918 | 0.3247 | 0.3631 | 0.2716 | 0.1609 | 0.4071 | 0.1397 | 0.2591 |
| S99 | 0.3860 | 0.3484 | 0.3430 | 0.3021 | 0.3177 | 0.2425 | 0.2900 | 0.2793 | 0.4055 | 0.2941 | 0.3377 | 0.2980 | 0.3256 | 0.3695 | 0.2752 | 0.1623 | 0.4115 | 0.1393 | 0.2612 |

Table S4 (Continued)

| Sample ID | S40 | S41 | S42 | S44 | S45 | S46 | S47 | S48 | S49 | S5 | S50 | S51 | S52 | S53 | S54 | S55 | S56 | S57 | S58 |
| --- | --- | --- | --- | --- | --- | --- | --- | --- | --- | --- | --- | --- | --- | --- | --- | --- | --- | --- | --- |
| S1 | 0.2887 | 0.2905 | 0.3939 | 0.3320 | 0.3418 | 0.3442 | 0.3330 | 0.2098 | 0.3838 | 0.3899 | 0.3833 | 0.3537 | 0.3229 | 0.3321 | 0.3368 | 0.3252 | 0.3348 | 0.3863 | 0.3645 |
| S10 | 0.2601 | 0.2587 | 0.3443 | 0.2678 | 0.1891 | 0.1872 | 0.2629 | 0.3512 | 0.3597 | 0.3501 | 0.3397 | 0.1553 | 0.2398 | 0.2562 | 0.2477 | 0.2355 | 0.2639 | 0.3356 | 0.3211 |
| S101 | 0.3238 | 0.3245 | 0.3200 | 0.3971 | 0.3582 | 0.3503 | 0.4040 | 0.3793 | 0.3106 | 0.2962 | 0.2951 | 0.3559 | 0.3227 | 0.3944 | 0.3988 | 0.3331 | 0.4016 | 0.3107 | 0.2869 |
| S102 | 0.3213 | 0.3199 | 0.3122 | 0.3959 | 0.3515 | 0.3449 | 0.4021 | 0.3771 | 0.3073 | 0.2939 | 0.2909 | 0.3501 | 0.3192 | 0.3971 | 0.3997 | 0.3273 | 0.4013 | 0.3027 | 0.2798 |
| S103 | 0.3620 | 0.3656 | 0.3353 | 0.4276 | 0.3955 | 0.3896 | 0.4355 | 0.4230 | 0.3199 | 0.3134 | 0.3077 | 0.3933 | 0.3638 | 0.4362 | 0.4369 | 0.3731 | 0.4341 | 0.3264 | 0.3171 |
| S104 | 0.3087 | 0.3085 | 0.2998 | 0.3773 | 0.3358 | 0.3335 | 0.3869 | 0.3610 | 0.2888 | 0.2767 | 0.2765 | 0.3400 | 0.3078 | 0.3823 | 0.3816 | 0.3198 | 0.3863 | 0.2895 | 0.2699 |
| S105 | 0.3431 | 0.3448 | 0.3186 | 0.4152 | 0.3782 | 0.3716 | 0.4251 | 0.4162 | 0.3044 | 0.2938 | 0.2896 | 0.3747 | 0.3450 | 0.4233 | 0.4240 | 0.3517 | 0.4221 | 0.3065 | 0.2984 |
| S11 | 0.3134 | 0.3137 | 0.0885 | 0.3764 | 0.3399 | 0.3352 | 0.3893 | 0.3941 | 0.3130 | 0.2960 | 0.2904 | 0.3360 | 0.3140 | 0.3973 | 0.3971 | 0.3217 | 0.3878 | 0.1283 | 0.2063 |
| S12 | 0.3176 | 0.3181 | 0.3949 | 0.2617 | 0.2765 | 0.2851 | 0.2539 | 0.3256 | 0.3933 | 0.3909 | 0.3851 | 0.2780 | 0.3071 | 0.2570 | 0.2543 | 0.3017 | 0.2547 | 0.3870 | 0.3685 |
| S13 | 0.3124 | 0.3130 | 0.0904 | 0.3763 | 0.3382 | 0.3386 | 0.3900 | 0.3950 | 0.3092 | 0.2943 | 0.2867 | 0.3369 | 0.3162 | 0.3956 | 0.3979 | 0.3249 | 0.3899 | 0.1294 | 0.2116 |
| S14 | 0.2863 | 0.2857 | 0.2556 | 0.3579 | 0.3184 | 0.3144 | 0.3643 | 0.3421 | 0.2663 | 0.2544 | 0.2521 | 0.3159 | 0.2904 | 0.3644 | 0.3663 | 0.3035 | 0.3637 | 0.2500 | 0.2385 |
| S15 | 0.3441 | 0.3478 | 0.3221 | 0.4160 | 0.3759 | 0.3713 | 0.4252 | 0.4150 | 0.3065 | 0.2915 | 0.2892 | 0.3705 | 0.3466 | 0.4239 | 0.4250 | 0.3558 | 0.4219 | 0.3093 | 0.3009 |
| S16 | 0.3823 | 0.3833 | 0.3578 | 0.4342 | 0.4113 | 0.4089 | 0.4418 | 0.4258 | 0.3203 | 0.3204 | 0.3167 | 0.4102 | 0.3901 | 0.4421 | 0.4438 | 0.3960 | 0.4393 | 0.3488 | 0.3397 |
| S17 | 0.3797 | 0.3844 | 0.3590 | 0.4333 | 0.4116 | 0.4087 | 0.4434 | 0.4231 | 0.3201 | 0.3245 | 0.3160 | 0.4100 | 0.3891 | 0.4411 | 0.4434 | 0.3954 | 0.4395 | 0.3512 | 0.3389 |
| S18 | 0.3832 | 0.3847 | 0.3594 | 0.4348 | 0.4136 | 0.4105 | 0.4421 | 0.4234 | 0.3221 | 0.3229 | 0.3172 | 0.4107 | 0.3898 | 0.4411 | 0.4454 | 0.3955 | 0.4406 | 0.3498 | 0.3399 |
| S19 | 0.3444 | 0.3449 | 0.3154 | 0.4173 | 0.3742 | 0.3713 | 0.4247 | 0.4164 | 0.3060 | 0.2936 | 0.2882 | 0.3696 | 0.3478 | 0.4252 | 0.4286 | 0.3560 | 0.4229 | 0.3060 | 0.3006 |
| S2 | 0.2940 | 0.2965 | 0.2910 | 0.3763 | 0.3286 | 0.3260 | 0.3826 | 0.3609 | 0.2861 | 0.2686 | 0.2665 | 0.3260 | 0.2986 | 0.3785 | 0.3803 | 0.3046 | 0.3829 | 0.2782 | 0.2627 |
| S20 | 0.3479 | 0.3515 | 0.3212 | 0.4187 | 0.3758 | 0.3739 | 0.4272 | 0.4200 | 0.3077 | 0.2987 | 0.2911 | 0.3726 | 0.3537 | 0.4265 | 0.4291 | 0.3613 | 0.4260 | 0.3069 | 0.3024 |
| S21 | 0.2860 | 0.2809 | 0.3923 | 0.3340 | 0.3388 | 0.3404 | 0.3344 | 0.2058 | 0.3846 | 0.3857 | 0.3825 | 0.3490 | 0.3187 | 0.3330 | 0.3378 | 0.3231 | 0.3309 | 0.3836 | 0.3623 |
| S22 | 0.3097 | 0.3063 | 0.3712 | 0.2203 | 0.2591 | 0.2649 | 0.2362 | 0.3302 | 0.3870 | 0.3850 | 0.3793 | 0.2595 | 0.2977 | 0.2601 | 0.2515 | 0.3001 | 0.2352 | 0.3642 | 0.3493 |
| S23 | 0.2547 | 0.2546 | 0.3374 | 0.2670 | 0.1642 | 0.1641 | 0.2613 | 0.3453 | 0.3556 | 0.3464 | 0.3382 | 0.1880 | 0.2410 | 0.2595 | 0.2539 | 0.2419 | 0.2614 | 0.3291 | 0.3130 |
| S24 | 0.2477 | 0.2473 | 0.3307 | 0.2739 | 0.2098 | 0.2064 | 0.2816 | 0.3432 | 0.3527 | 0.3416 | 0.3319 | 0.1933 | 0.2264 | 0.2499 | 0.2535 | 0.2187 | 0.2752 | 0.3246 | 0.3094 |
| S26 | 0.3291 | 0.3283 | 0.3260 | 0.3824 | 0.3584 | 0.3547 | 0.3861 | 0.3428 | 0.3089 | 0.2991 | 0.2990 | 0.3547 | 0.3300 | 0.3847 | 0.3910 | 0.3405 | 0.3852 | 0.3116 | 0.2984 |
| S27 | 0.3250 | 0.3209 | 0.2867 | 0.3413 | 0.3329 | 0.3323 | 0.3500 | 0.3478 | 0.3190 | 0.3175 | 0.3090 | 0.3341 | 0.3244 | 0.3543 | 0.3523 | 0.3284 | 0.3462 | 0.2745 | 0.2661 |
| S28 | 0.2787 | 0.2790 | 0.2093 | 0.3496 | 0.3046 | 0.3034 | 0.3610 | 0.3538 | 0.2637 | 0.2425 | 0.2409 | 0.3024 | 0.2799 | 0.3623 | 0.3601 | 0.2896 | 0.3585 | 0.1981 | 0.1958 |
| S29 | 0.3402 | 0.3425 | 0.3108 | 0.4120 | 0.3699 | 0.3666 | 0.4163 | 0.4116 | 0.2986 | 0.2868 | 0.2793 | 0.3660 | 0.3428 | 0.4199 | 0.4202 | 0.3548 | 0.4169 | 0.2983 | 0.2939 |
| S3 | 0.3069 | 0.3104 | 0.3021 | 0.3797 | 0.3392 | 0.3381 | 0.3874 | 0.3576 | 0.2906 | 0.2766 | 0.2730 | 0.3372 | 0.3089 | 0.3860 | 0.3890 | 0.3179 | 0.3876 | 0.2901 | 0.2749 |
| S30 | 0.3984 | 0.4031 | 0.4196 | 0.3735 | 0.3992 | 0.3982 | 0.3807 | 0.3914 | 0.3900 | 0.4001 | 0.3948 | 0.3968 | 0.4001 | 0.3908 | 0.3902 | 0.4072 | 0.3840 | 0.4139 | 0.3964 |
| S31 | 0.3444 | 0.3477 | 0.3135 | 0.4145 | 0.3740 | 0.3695 | 0.4225 | 0.4155 | 0.3015 | 0.2874 | 0.2862 | 0.3671 | 0.3468 | 0.4216 | 0.4256 | 0.3551 | 0.4212 | 0.3049 | 0.2970 |
| S32 | 0.2471 | 0.2429 | 0.3282 | 0.2769 | 0.2010 | 0.2005 | 0.2788 | 0.3468 | 0.3468 | 0.3363 | 0.3271 | 0.1863 | 0.2259 | 0.2554 | 0.2584 | 0.2218 | 0.2755 | 0.3180 | 0.3064 |
| S33 | 0.3458 | 0.3467 | 0.3169 | 0.4173 | 0.3763 | 0.3733 | 0.4286 | 0.4189 | 0.3056 | 0.2938 | 0.2895 | 0.3740 | 0.3496 | 0.4264 | 0.4285 | 0.3596 | 0.4227 | 0.3033 | 0.3009 |
| S34 | 0.2141 | 0.2127 | 0.3220 | 0.3052 | 0.2457 | 0.2462 | 0.3050 | 0.3166 | 0.3370 | 0.3257 | 0.3180 | 0.2382 | 0.1602 | 0.2892 | 0.2970 | 0.1481 | 0.3059 | 0.3138 | 0.2964 |
| S35 | 0.3772 | 0.3777 | 0.3805 | 0.3950 | 0.3719 | 0.3727 | 0.3953 | 0.4166 | 0.3632 | 0.3637 | 0.3576 | 0.3641 | 0.3772 | 0.4019 | 0.4049 | 0.3814 | 0.3957 | 0.3710 | 0.3604 |
| S36 | 0.2873 | 0.2870 | 0.1892 | 0.3526 | 0.3087 | 0.3048 | 0.3662 | 0.3716 | 0.2895 | 0.2698 | 0.2646 | 0.3070 | 0.2831 | 0.3673 | 0.3693 | 0.2967 | 0.3652 | 0.1898 | 0.1898 |
| S37 | 0.3085 | 0.3064 | 0.2875 | 0.3863 | 0.3366 | 0.3374 | 0.3921 | 0.3808 | 0.1637 | 0.1510 | 0.1458 | 0.3350 | 0.3056 | 0.3910 | 0.3932 | 0.3159 | 0.3894 | 0.2787 | 0.2713 |
| S38 | 0.3831 | 0.3831 | 0.4246 | 0.3366 | 0.3656 | 0.3686 | 0.3395 | 0.3766 | 0.4009 | 0.4079 | 0.4034 | 0.3671 | 0.3829 | 0.3560 | 0.3543 | 0.3856 | 0.3432 | 0.4181 | 0.3997 |
| S39 | 0.3129 | 0.3126 | 0.2917 | 0.3919 | 0.3458 | 0.3425 | 0.4002 | 0.3843 | 0.1744 | 0.1331 | 0.1580 | 0.3434 | 0.3110 | 0.3984 | 0.4000 | 0.3234 | 0.4003 | 0.2836 | 0.2795 |
| S4 | 0.2910 | 0.2901 | 0.2756 | 0.3689 | 0.3170 | 0.3149 | 0.3763 | 0.3572 | 0.2748 | 0.2591 | 0.2561 | 0.3188 | 0.2893 | 0.3718 | 0.3730 | 0.2992 | 0.3778 | 0.2646 | 0.2526 |
| S40 | 0.0000 | 0.1510 | 0.3161 | 0.3168 | 0.2589 | 0.2557 | 0.3199 | 0.2853 | 0.3255 | 0.3142 | 0.3082 | 0.2554 | 0.2128 | 0.3115 | 0.3139 | 0.2189 | 0.3187 | 0.3046 | 0.2889 |
| S41 | 0.1510 | 0.0000 | 0.3140 | 0.3125 | 0.2529 | 0.2510 | 0.3149 | 0.2837 | 0.3302 | 0.3135 | 0.3058 | 0.2524 | 0.2142 | 0.3101 | 0.3143 | 0.2190 | 0.3134 | 0.3024 | 0.2875 |
| S42 | 0.3161 | 0.3140 | 0.0000 | 0.3779 | 0.3361 | 0.3354 | 0.3878 | 0.3942 | 0.3126 | 0.2969 | 0.2888 | 0.3357 | 0.3179 | 0.3940 | 0.3966 | 0.3262 | 0.3884 | 0.1250 | 0.2069 |
| S44 | 0.3168 | 0.3125 | 0.3779 | 0.0000 | 0.2638 | 0.2659 | 0.2362 | 0.3290 | 0.3956 | 0.3928 | 0.3874 | 0.2644 | 0.3072 | 0.2596 | 0.2477 | 0.3058 | 0.2379 | 0.3712 | 0.3571 |
| S45 | 0.2589 | 0.2529 | 0.3361 | 0.2638 | 0.0000 | 0.1643 | 0.2638 | 0.3428 | 0.3589 | 0.3470 | 0.3386 | 0.1924 | 0.2429 | 0.2612 | 0.2526 | 0.2423 | 0.2625 | 0.3296 | 0.3157 |
| S46 | 0.2557 | 0.2510 | 0.3354 | 0.2659 | 0.1643 | 0.0000 | 0.2655 | 0.3453 | 0.3577 | 0.3439 | 0.3365 | 0.1911 | 0.2407 | 0.2662 | 0.2528 | 0.2402 | 0.2662 | 0.3273 | 0.3101 |
| S47 | 0.3199 | 0.3149 | 0.3878 | 0.2362 | 0.2638 | 0.2655 | 0.0000 | 0.3306 | 0.4019 | 0.3990 | 0.3928 | 0.2615 | 0.3101 | 0.2526 | 0.2342 | 0.3071 | 0.1990 | 0.3818 | 0.3706 |
| S48 | 0.2853 | 0.2837 | 0.3942 | 0.3290 | 0.3428 | 0.3453 | 0.3306 | 0.0000 | 0.3803 | 0.3889 | 0.3821 | 0.3516 | 0.3189 | 0.3314 | 0.3363 | 0.3215 | 0.3322 | 0.3870 | 0.3635 |
| S49 | 0.3255 | 0.3302 | 0.3126 | 0.3956 | 0.3589 | 0.3577 | 0.4019 | 0.3803 | 0.0000 | 0.1720 | 0.1239 | 0.3568 | 0.3305 | 0.4007 | 0.4034 | 0.3384 | 0.4013 | 0.3040 | 0.2926 |
| S5 | 0.3142 | 0.3135 | 0.2969 | 0.3928 | 0.3470 | 0.3439 | 0.3990 | 0.3889 | 0.1720 | 0.0000 | 0.1587 | 0.3425 | 0.3179 | 0.3963 | 0.3989 | 0.3270 | 0.3996 | 0.2861 | 0.2804 |
| S50 | 0.3082 | 0.3058 | 0.2888 | 0.3874 | 0.3386 | 0.3365 | 0.3928 | 0.3821 | 0.1239 | 0.1587 | 0.0000 | 0.3358 | 0.3107 | 0.3904 | 0.3911 | 0.3173 | 0.3938 | 0.2795 | 0.2708 |
| S51 | 0.2554 | 0.2524 | 0.3357 | 0.2644 | 0.1924 | 0.1911 | 0.2615 | 0.3516 | 0.3568 | 0.3425 | 0.3358 | 0.0000 | 0.2374 | 0.2585 | 0.2543 | 0.2365 | 0.2635 | 0.3255 | 0.3152 |
| S52 | 0.2128 | 0.2142 | 0.3179 | 0.3072 | 0.2429 | 0.2407 | 0.3101 | 0.3189 | 0.3305 | 0.3179 | 0.3107 | 0.2374 | 0.0000 | 0.2937 | 0.3007 | 0.1635 | 0.3092 | 0.3045 | 0.2881 |
| S53 | 0.3115 | 0.3101 | 0.3940 | 0.2596 | 0.2612 | 0.2662 | 0.2526 | 0.3314 | 0.4007 | 0.3963 | 0.3904 | 0.2585 | 0.2937 | 0.0000 | 0.2192 | 0.2873 | 0.2485 | 0.3871 | 0.3718 |
| S54 | 0.3139 | 0.3143 | 0.3966 | 0.2477 | 0.2526 | 0.2528 | 0.2342 | 0.3363 | 0.4034 | 0.3989 | 0.3911 | 0.2543 | 0.3007 | 0.2192 | 0.0000 | 0.2966 | 0.2368 | 0.3903 | 0.3698 |
| S55 | 0.2189 | 0.2190 | 0.3262 | 0.3058 | 0.2423 | 0.2402 | 0.3071 | 0.3215 | 0.3384 | 0.3270 | 0.3173 | 0.2365 | 0.1635 | 0.2873 | 0.2966 | 0.0000 | 0.3054 | 0.3151 | 0.3002 |
| S56 | 0.3187 | 0.3134 | 0.3884 | 0.2379 | 0.2625 | 0.2662 | 0.1990 | 0.3322 | 0.4013 | 0.3996 | 0.3938 | 0.2635 | 0.3092 | 0.2485 | 0.2368 | 0.3054 | 0.0000 | 0.3810 | 0.3680 |
| S57 | 0.3046 | 0.3024 | 0.1250 | 0.3712 | 0.3296 | 0.3273 | 0.3818 | 0.3870 | 0.3040 | 0.2861 | 0.2795 | 0.3255 | 0.3045 | 0.3871 | 0.3903 | 0.3151 | 0.3810 | 0.0000 | 0.1999 |
| S58 | 0.2889 | 0.2875 | 0.2069 | 0.3571 | 0.3157 | 0.3101 | 0.3706 | 0.3635 | 0.2926 | 0.2804 | 0.2708 | 0.3152 | 0.2881 | 0.3718 | 0.3698 | 0.3002 | 0.3680 | 0.1999 | 0.0000 |
| S59 | 0.3017 | 0.3022 | 0.2107 | 0.3573 | 0.3276 | 0.3240 | 0.3699 | 0.3662 | 0.3013 | 0.2910 | 0.2862 | 0.3266 | 0.3050 | 0.3760 | 0.3750 | 0.3142 | 0.3695 | 0.2024 | 0.1426 |
| S6 | 0.2882 | 0.2886 | 0.2776 | 0.3665 | 0.3175 | 0.3108 | 0.3756 | 0.3556 | 0.2748 | 0.2592 | 0.2566 | 0.3161 | 0.2853 | 0.3705 | 0.3709 | 0.2961 | 0.3742 | 0.2660 | 0.2508 |
| S60 | 0.3106 | 0.3048 | 0.3917 | 0.2647 | 0.2592 | 0.2629 | 0.2498 | 0.3315 | 0.3959 | 0.3950 | 0.3872 | 0.2580 | 0.2918 | 0.2153 | 0.2179 | 0.2878 | 0.2480 | 0.3828 | 0.3642 |
| S61 | 0.2755 | 0.2741 | 0.2422 | 0.3530 | 0.3070 | 0.3060 | 0.3596 | 0.3464 | 0.2626 | 0.2452 | 0.2385 | 0.3054 | 0.2752 | 0.3612 | 0.3647 | 0.2910 | 0.3597 | 0.2363 | 0.2257 |
| S62 | 0.2730 | 0.2716 | 0.2405 | 0.3520 | 0.3046 | 0.3012 | 0.3570 | 0.3497 | 0.2618 | 0.2403 | 0.2333 | 0.2999 | 0.2743 | 0.3602 | 0.3609 | 0.2855 | 0.3572 | 0.2287 | 0.2205 |
| S63 | 0.3067 | 0.3078 | 0.3031 | 0.3859 | 0.3405 | 0.3330 | 0.3940 | 0.3762 | 0.2965 | 0.2806 | 0.2778 | 0.3340 | 0.3036 | 0.3864 | 0.3905 | 0.3139 | 0.3905 | 0.2949 | 0.2725 |
| S65 | 0.3165 | 0.3139 | 0.0872 | 0.3760 | 0.3400 | 0.3372 | 0.3887 | 0.3943 | 0.3129 | 0.2962 | 0.2904 | 0.3368 | 0.3173 | 0.3946 | 0.3972 | 0.3254 | 0.3891 | 0.1308 | 0.2097 |
| S7 | 0.3412 | 0.3440 | 0.3165 | 0.4162 | 0.3767 | 0.3698 | 0.4261 | 0.4134 | 0.3023 | 0.2943 | 0.2870 | 0.3717 | 0.3443 | 0.4233 | 0.4275 | 0.3546 | 0.4251 | 0.3080 | 0.3009 |
| S71 | 0.3067 | 0.3061 | 0.3023 | 0.3865 | 0.3385 | 0.3319 | 0.3956 | 0.3734 | 0.2965 | 0.2810 | 0.2806 | 0.3301 | 0.3052 | 0.3870 | 0.3890 | 0.3156 | 0.3919 | 0.2927 | 0.2708 |
| S72 | 0.3079 | 0.3069 | 0.3031 | 0.3845 | 0.3396 | 0.3333 | 0.3933 | 0.3771 | 0.2984 | 0.2795 | 0.2786 | 0.3341 | 0.3046 | 0.3847 | 0.3871 | 0.3137 | 0.3904 | 0.2936 | 0.2725 |
| S73 | 0.2845 | 0.2812 | 0.3892 | 0.3304 | 0.3405 | 0.3411 | 0.3327 | 0.2126 | 0.3829 | 0.3806 | 0.3800 | 0.3502 | 0.3168 | 0.3340 | 0.3380 | 0.3197 | 0.3336 | 0.3818 | 0.3597 |
| S74 | 0.2995 | 0.2980 | 0.2061 | 0.3583 | 0.3261 | 0.3227 | 0.3700 | 0.3687 | 0.2970 | 0.2861 | 0.2807 | 0.3237 | 0.3005 | 0.3768 | 0.3745 | 0.3105 | 0.3673 | 0.1992 | 0.1416 |
| S75 | 0.2893 | 0.2880 | 0.1952 | 0.3553 | 0.3155 | 0.3116 | 0.3701 | 0.3715 | 0.2910 | 0.2769 | 0.2691 | 0.3131 | 0.2874 | 0.3741 | 0.3724 | 0.2982 | 0.3661 | 0.1860 | 0.1341 |
| S76 | 0.3368 | 0.3384 | 0.3145 | 0.4111 | 0.3718 | 0.3653 | 0.4222 | 0.4126 | 0.3028 | 0.2905 | 0.2857 | 0.3684 | 0.3374 | 0.4204 | 0.4211 | 0.3498 | 0.4202 | 0.3010 | 0.2958 |
| S77 | 0.3416 | 0.3436 | 0.3172 | 0.4161 | 0.3769 | 0.3721 | 0.4230 | 0.4175 | 0.3013 | 0.2906 | 0.2855 | 0.3698 | 0.3443 | 0.4217 | 0.4245 | 0.3523 | 0.4233 | 0.3055 | 0.3004 |
| S78 | 0.3386 | 0.3410 | 0.3181 | 0.4128 | 0.3733 | 0.3689 | 0.4208 | 0.4116 | 0.3049 | 0.2930 | 0.2848 | 0.3699 | 0.3426 | 0.4213 | 0.4221 | 0.3515 | 0.4216 | 0.3043 | 0.2987 |
| S79 | 0.2570 | 0.2522 | 0.3364 | 0.2665 | 0.1953 | 0.1971 | 0.2651 | 0.3489 | 0.3562 | 0.3475 | 0.3363 | 0.1763 | 0.2397 | 0.2513 | 0.2375 | 0.2356 | 0.2609 | 0.3301 | 0.3139 |
| S8 | 0.3397 | 0.3427 | 0.3186 | 0.4148 | 0.3747 | 0.3708 | 0.4273 | 0.4151 | 0.3037 | 0.2931 | 0.2891 | 0.3705 | 0.3441 | 0.4257 | 0.4245 | 0.3532 | 0.4259 | 0.3074 | 0.3003 |
| S80 | 0.3116 | 0.3112 | 0.3950 | 0.2583 | 0.2593 | 0.2565 | 0.2460 | 0.3339 | 0.3985 | 0.3994 | 0.3883 | 0.2602 | 0.2975 | 0.1940 | 0.1431 | 0.2912 | 0.2429 | 0.3892 | 0.3677 |
| S84 | 0.2099 | 0.2101 | 0.3211 | 0.3039 | 0.2422 | 0.2407 | 0.3043 | 0.3196 | 0.3358 | 0.3242 | 0.3136 | 0.2334 | 0.1540 | 0.2860 | 0.2936 | 0.1496 | 0.3037 | 0.3098 | 0.2935 |
| S85 | 0.3034 | 0.3052 | 0.2995 | 0.3863 | 0.3355 | 0.3313 | 0.3934 | 0.3729 | 0.2911 | 0.2738 | 0.2734 | 0.3318 | 0.3016 | 0.3852 | 0.3875 | 0.3111 | 0.3915 | 0.2887 | 0.2672 |
| S86 | 0.2377 | 0.2379 | 0.3081 | 0.2734 | 0.2075 | 0.2072 | 0.2835 | 0.3403 | 0.3281 | 0.3097 | 0.3032 | 0.1913 | 0.2215 | 0.2856 | 0.2785 | 0.2311 | 0.2822 | 0.2961 | 0.2848 |
| S87 | 0.1532 | 0.1519 | 0.3211 | 0.3158 | 0.2607 | 0.2592 | 0.3212 | 0.2783 | 0.3269 | 0.3171 | 0.3098 | 0.2619 | 0.2240 | 0.3119 | 0.3144 | 0.2319 | 0.3214 | 0.3089 | 0.2948 |
| S88 | 0.3548 | 0.3569 | 0.3549 | 0.3955 | 0.3859 | 0.3848 | 0.4026 | 0.3534 | 0.3294 | 0.3276 | 0.3268 | 0.3873 | 0.3607 | 0.3998 | 0.4023 | 0.3685 | 0.4024 | 0.3455 | 0.3229 |
| S89 | 0.2868 | 0.2859 | 0.1918 | 0.3518 | 0.3102 | 0.3072 | 0.3652 | 0.3712 | 0.2904 | 0.2697 | 0.2616 | 0.3054 | 0.2835 | 0.3685 | 0.3676 | 0.2960 | 0.3646 | 0.1914 | 0.1921 |
| S9 | 0.3130 | 0.3100 | 0.3962 | 0.2552 | 0.2512 | 0.2573 | 0.2412 | 0.3392 | 0.4023 | 0.3997 | 0.3932 | 0.2551 | 0.3004 | 0.2143 | 0.2052 | 0.2940 | 0.2426 | 0.3879 | 0.3720 |
| S90 | 0.2852 | 0.2834 | 0.2450 | 0.3661 | 0.3164 | 0.3122 | 0.3787 | 0.3743 | 0.2813 | 0.2596 | 0.2528 | 0.3107 | 0.2835 | 0.3768 | 0.3799 | 0.2955 | 0.3758 | 0.2339 | 0.2331 |
| S91 | 0.2909 | 0.2920 | 0.2518 | 0.3684 | 0.3218 | 0.3199 | 0.3805 | 0.3738 | 0.2817 | 0.2645 | 0.2556 | 0.3159 | 0.2870 | 0.3778 | 0.3777 | 0.2993 | 0.3767 | 0.2387 | 0.2352 |
| S92 | 0.2891 | 0.2897 | 0.2526 | 0.3651 | 0.3206 | 0.3165 | 0.3756 | 0.3743 | 0.2822 | 0.2654 | 0.2566 | 0.3116 | 0.2865 | 0.3758 | 0.3783 | 0.2983 | 0.3762 | 0.2399 | 0.2400 |
| S93 | 0.2900 | 0.2885 | 0.2503 | 0.3635 | 0.3194 | 0.3163 | 0.3746 | 0.3741 | 0.2791 | 0.2596 | 0.2526 | 0.3120 | 0.2884 | 0.3767 | 0.3753 | 0.2986 | 0.3743 | 0.2401 | 0.2372 |
| S95 | 0.2952 | 0.2943 | 0.2566 | 0.3711 | 0.3258 | 0.3222 | 0.3791 | 0.3758 | 0.2879 | 0.2704 | 0.2583 | 0.3197 | 0.2898 | 0.3791 | 0.3787 | 0.3027 | 0.3801 | 0.2465 | 0.2425 |
| S96 | 0.3167 | 0.3155 | 0.2949 | 0.3924 | 0.3507 | 0.3470 | 0.4014 | 0.3876 | 0.1817 | 0.1463 | 0.1646 | 0.3441 | 0.3174 | 0.4003 | 0.4017 | 0.3283 | 0.3993 | 0.2822 | 0.2767 |
| S97 | 0.2866 | 0.2887 | 0.3893 | 0.3339 | 0.3419 | 0.3456 | 0.3383 | 0.2235 | 0.3799 | 0.3811 | 0.3756 | 0.3493 | 0.3181 | 0.3380 | 0.3404 | 0.3226 | 0.3379 | 0.3779 | 0.3563 |
| S98 | 0.3156 | 0.3156 | 0.2942 | 0.3928 | 0.3482 | 0.3466 | 0.4001 | 0.3882 | 0.1821 | 0.1455 | 0.1633 | 0.3432 | 0.3140 | 0.4010 | 0.3994 | 0.3262 | 0.3970 | 0.2814 | 0.2769 |
| S99 | 0.3187 | 0.3168 | 0.2978 | 0.3937 | 0.3520 | 0.3465 | 0.4020 | 0.3878 | 0.1831 | 0.1450 | 0.1675 | 0.3450 | 0.3164 | 0.4011 | 0.4019 | 0.3273 | 0.3991 | 0.2875 | 0.2791 |

Table S4 (Continued)

| Sample ID | S59 | S6 | S60 | S61 | S62 | S63 | S65 | S7 | S71 | S72 | S73 | S74 | S75 | S76 | S77 | S78 | S79 | S8 | S80 |
| --- | --- | --- | --- | --- | --- | --- | --- | --- | --- | --- | --- | --- | --- | --- | --- | --- | --- | --- | --- |
| S1 | 0.3657 | 0.3558 | 0.3310 | 0.3482 | 0.3501 | 0.3766 | 0.3928 | 0.4156 | 0.3740 | 0.3755 | 0.2190 | 0.3673 | 0.3704 | 0.4143 | 0.4151 | 0.4133 | 0.3530 | 0.4140 | 0.3370 |
| S10 | 0.3320 | 0.3201 | 0.2580 | 0.3126 | 0.3097 | 0.3381 | 0.3425 | 0.3804 | 0.3366 | 0.3372 | 0.3494 | 0.3303 | 0.3195 | 0.3723 | 0.3772 | 0.3748 | 0.1692 | 0.3744 | 0.2523 |
| S101 | 0.3058 | 0.2203 | 0.3936 | 0.2709 | 0.2698 | 0.1633 | 0.3235 | 0.3321 | 0.1622 | 0.1647 | 0.3743 | 0.3056 | 0.2957 | 0.3299 | 0.3285 | 0.3291 | 0.3519 | 0.3323 | 0.3984 |
| S102 | 0.3039 | 0.2146 | 0.3916 | 0.2647 | 0.2619 | 0.1597 | 0.3150 | 0.3256 | 0.1584 | 0.1608 | 0.3763 | 0.2998 | 0.2905 | 0.3251 | 0.3222 | 0.3239 | 0.3486 | 0.3246 | 0.4001 |
| S103 | 0.3274 | 0.3110 | 0.4282 | 0.2841 | 0.2809 | 0.3333 | 0.3362 | 0.1822 | 0.3320 | 0.3362 | 0.4205 | 0.3232 | 0.3169 | 0.1812 | 0.1665 | 0.1789 | 0.3905 | 0.1823 | 0.4348 |
| S104 | 0.2852 | 0.1642 | 0.3776 | 0.2504 | 0.2488 | 0.2142 | 0.3006 | 0.3090 | 0.2158 | 0.2171 | 0.3592 | 0.2821 | 0.2741 | 0.3086 | 0.3080 | 0.3083 | 0.3391 | 0.3104 | 0.3816 |
| S105 | 0.3109 | 0.2912 | 0.4212 | 0.2661 | 0.2608 | 0.3165 | 0.3192 | 0.1772 | 0.3135 | 0.3183 | 0.4120 | 0.3063 | 0.2954 | 0.1784 | 0.1704 | 0.1735 | 0.3747 | 0.1798 | 0.4231 |
| S11 | 0.2090 | 0.2732 | 0.3923 | 0.2402 | 0.2378 | 0.3027 | 0.0891 | 0.3158 | 0.3030 | 0.3009 | 0.3914 | 0.2059 | 0.1959 | 0.3118 | 0.3162 | 0.3163 | 0.3372 | 0.3181 | 0.3950 |
| S12 | 0.3709 | 0.3720 | 0.2551 | 0.3609 | 0.3582 | 0.3911 | 0.3930 | 0.4149 | 0.3916 | 0.3907 | 0.3305 | 0.3711 | 0.3669 | 0.4130 | 0.4120 | 0.4106 | 0.2755 | 0.4127 | 0.2578 |
| S13 | 0.2122 | 0.2758 | 0.3920 | 0.2416 | 0.2377 | 0.3033 | 0.0815 | 0.3158 | 0.3026 | 0.3014 | 0.3900 | 0.2084 | 0.1974 | 0.3130 | 0.3151 | 0.3153 | 0.3368 | 0.3175 | 0.3961 |
| S14 | 0.2477 | 0.2375 | 0.3600 | 0.1312 | 0.1419 | 0.2661 | 0.2605 | 0.2778 | 0.2641 | 0.2664 | 0.3408 | 0.2460 | 0.2365 | 0.2727 | 0.2768 | 0.2786 | 0.3172 | 0.2795 | 0.3647 |
| S15 | 0.3137 | 0.2898 | 0.4188 | 0.2700 | 0.2644 | 0.3174 | 0.3240 | 0.1860 | 0.3174 | 0.3190 | 0.4147 | 0.3094 | 0.2990 | 0.1839 | 0.1802 | 0.1842 | 0.3728 | 0.1880 | 0.4255 |
| S16 | 0.3465 | 0.3309 | 0.4409 | 0.3093 | 0.3082 | 0.3558 | 0.3594 | 0.2377 | 0.3574 | 0.3580 | 0.4223 | 0.3446 | 0.3399 | 0.2428 | 0.2384 | 0.2414 | 0.4148 | 0.2391 | 0.4432 |
| S17 | 0.3459 | 0.3287 | 0.4378 | 0.3092 | 0.3079 | 0.3533 | 0.3611 | 0.2368 | 0.3576 | 0.3595 | 0.4231 | 0.3430 | 0.3388 | 0.2404 | 0.2392 | 0.2368 | 0.4111 | 0.2364 | 0.4405 |
| S18 | 0.3450 | 0.3292 | 0.4384 | 0.3075 | 0.3096 | 0.3545 | 0.3608 | 0.2362 | 0.3555 | 0.3582 | 0.4199 | 0.3441 | 0.3384 | 0.2429 | 0.2367 | 0.2378 | 0.4152 | 0.2376 | 0.4433 |
| S19 | 0.3100 | 0.2890 | 0.4217 | 0.2644 | 0.2636 | 0.3187 | 0.3152 | 0.1976 | 0.3171 | 0.3196 | 0.4159 | 0.3081 | 0.2971 | 0.1960 | 0.1923 | 0.1932 | 0.3744 | 0.1958 | 0.4248 |
| S2 | 0.2816 | 0.1998 | 0.3748 | 0.2427 | 0.2374 | 0.1908 | 0.2914 | 0.3032 | 0.1878 | 0.1927 | 0.3579 | 0.2778 | 0.2693 | 0.2979 | 0.2970 | 0.2974 | 0.3281 | 0.3022 | 0.3793 |
| S20 | 0.3120 | 0.2970 | 0.4224 | 0.2724 | 0.2680 | 0.3221 | 0.3234 | 0.2040 | 0.3229 | 0.3244 | 0.4168 | 0.3133 | 0.2990 | 0.1955 | 0.1976 | 0.1974 | 0.3794 | 0.2007 | 0.4254 |
| S21 | 0.3672 | 0.3559 | 0.3296 | 0.3457 | 0.3504 | 0.3735 | 0.3932 | 0.4134 | 0.3742 | 0.3743 | 0.2103 | 0.3684 | 0.3686 | 0.4108 | 0.4140 | 0.4123 | 0.3488 | 0.4124 | 0.3349 |
| S22 | 0.3506 | 0.3587 | 0.2596 | 0.3476 | 0.3482 | 0.3801 | 0.3688 | 0.4105 | 0.3795 | 0.3779 | 0.3316 | 0.3536 | 0.3498 | 0.4051 | 0.4114 | 0.4066 | 0.2647 | 0.4097 | 0.2584 |
| S23 | 0.3223 | 0.3175 | 0.2631 | 0.3041 | 0.3034 | 0.3355 | 0.3377 | 0.3724 | 0.3381 | 0.3338 | 0.3403 | 0.3220 | 0.3137 | 0.3684 | 0.3713 | 0.3694 | 0.1908 | 0.3729 | 0.2577 |
| S24 | 0.3225 | 0.3082 | 0.2537 | 0.2995 | 0.2960 | 0.3251 | 0.3333 | 0.3690 | 0.3250 | 0.3268 | 0.3416 | 0.3184 | 0.3106 | 0.3641 | 0.3657 | 0.3650 | 0.1905 | 0.3671 | 0.2520 |
| S26 | 0.3093 | 0.2320 | 0.3806 | 0.2731 | 0.2733 | 0.2308 | 0.3286 | 0.3357 | 0.2270 | 0.2309 | 0.3422 | 0.3082 | 0.3017 | 0.3303 | 0.3321 | 0.3321 | 0.3599 | 0.3381 | 0.3884 |
| S27 | 0.2644 | 0.2942 | 0.3506 | 0.2688 | 0.2693 | 0.3211 | 0.2859 | 0.3293 | 0.3216 | 0.3201 | 0.3476 | 0.2658 | 0.2646 | 0.3311 | 0.3296 | 0.3307 | 0.3345 | 0.3317 | 0.3504 |
| S28 | 0.2097 | 0.2242 | 0.3552 | 0.1973 | 0.1931 | 0.2534 | 0.2110 | 0.2664 | 0.2523 | 0.2550 | 0.3517 | 0.2042 | 0.1927 | 0.2628 | 0.2631 | 0.2631 | 0.3032 | 0.2660 | 0.3581 |
| S29 | 0.3038 | 0.2844 | 0.4146 | 0.2609 | 0.2581 | 0.3099 | 0.3121 | 0.2014 | 0.3104 | 0.3128 | 0.4086 | 0.3021 | 0.2902 | 0.1976 | 0.1962 | 0.1952 | 0.3684 | 0.1997 | 0.4190 |
| S3 | 0.2908 | 0.2054 | 0.3805 | 0.2515 | 0.2497 | 0.1932 | 0.3042 | 0.3115 | 0.1972 | 0.1971 | 0.3536 | 0.2893 | 0.2813 | 0.3083 | 0.3111 | 0.3099 | 0.3408 | 0.3116 | 0.3874 |
| S30 | 0.3950 | 0.3916 | 0.3895 | 0.3756 | 0.3772 | 0.4175 | 0.4167 | 0.3583 | 0.4163 | 0.4185 | 0.3901 | 0.3969 | 0.4000 | 0.3596 | 0.3547 | 0.3532 | 0.4016 | 0.3562 | 0.3911 |
| S31 | 0.3086 | 0.2910 | 0.4180 | 0.2642 | 0.2629 | 0.3161 | 0.3175 | 0.2000 | 0.3154 | 0.3173 | 0.4129 | 0.3036 | 0.2957 | 0.1987 | 0.1959 | 0.1984 | 0.3738 | 0.1989 | 0.4230 |
| S32 | 0.3181 | 0.3063 | 0.2583 | 0.2953 | 0.2903 | 0.3231 | 0.3309 | 0.3615 | 0.3253 | 0.3239 | 0.3422 | 0.3156 | 0.3023 | 0.3564 | 0.3627 | 0.3584 | 0.1917 | 0.3616 | 0.2581 |
| S33 | 0.3154 | 0.2920 | 0.4219 | 0.2688 | 0.2638 | 0.3180 | 0.3183 | 0.1985 | 0.3197 | 0.3203 | 0.4162 | 0.3104 | 0.3009 | 0.1976 | 0.1960 | 0.1952 | 0.3747 | 0.1967 | 0.4239 |
| S34 | 0.3109 | 0.2970 | 0.2891 | 0.2885 | 0.2832 | 0.3124 | 0.3229 | 0.3537 | 0.3136 | 0.3106 | 0.3140 | 0.3094 | 0.2989 | 0.3488 | 0.3522 | 0.3495 | 0.2415 | 0.3558 | 0.2935 |
| S35 | 0.3657 | 0.3526 | 0.3964 | 0.3327 | 0.3317 | 0.3785 | 0.3800 | 0.2836 | 0.3768 | 0.3809 | 0.4128 | 0.3636 | 0.3620 | 0.2819 | 0.2825 | 0.2820 | 0.3698 | 0.2837 | 0.4022 |
| S36 | 0.1966 | 0.2520 | 0.3671 | 0.2224 | 0.2151 | 0.2768 | 0.1953 | 0.2944 | 0.2745 | 0.2772 | 0.3656 | 0.1974 | 0.1863 | 0.2884 | 0.2951 | 0.2922 | 0.3071 | 0.2986 | 0.3661 |
| S37 | 0.2830 | 0.2545 | 0.3869 | 0.2380 | 0.2325 | 0.2737 | 0.2918 | 0.2869 | 0.2738 | 0.2735 | 0.3795 | 0.2795 | 0.2693 | 0.2837 | 0.2859 | 0.2841 | 0.3358 | 0.2866 | 0.3905 |
| S38 | 0.3984 | 0.3969 | 0.3555 | 0.3818 | 0.3855 | 0.4222 | 0.4230 | 0.3896 | 0.4198 | 0.4199 | 0.3746 | 0.4019 | 0.4032 | 0.3892 | 0.3902 | 0.3879 | 0.3709 | 0.3921 | 0.3561 |
| S39 | 0.2929 | 0.2575 | 0.3929 | 0.2417 | 0.2359 | 0.2810 | 0.2964 | 0.2888 | 0.2802 | 0.2782 | 0.3830 | 0.2861 | 0.2769 | 0.2867 | 0.2881 | 0.2885 | 0.3451 | 0.2920 | 0.3976 |
| S4 | 0.2679 | 0.1187 | 0.3684 | 0.2306 | 0.2245 | 0.2028 | 0.2765 | 0.2912 | 0.2034 | 0.2030 | 0.3555 | 0.2654 | 0.2524 | 0.2872 | 0.2889 | 0.2903 | 0.3172 | 0.2897 | 0.3729 |
| S40 | 0.3017 | 0.2882 | 0.3106 | 0.2755 | 0.2730 | 0.3067 | 0.3165 | 0.3412 | 0.3067 | 0.3079 | 0.2845 | 0.2995 | 0.2893 | 0.3368 | 0.3416 | 0.3386 | 0.2570 | 0.3397 | 0.3116 |
| S41 | 0.3022 | 0.2886 | 0.3048 | 0.2741 | 0.2716 | 0.3078 | 0.3139 | 0.3440 | 0.3061 | 0.3069 | 0.2812 | 0.2980 | 0.2880 | 0.3384 | 0.3436 | 0.3410 | 0.2522 | 0.3427 | 0.3112 |
| S42 | 0.2107 | 0.2776 | 0.3917 | 0.2422 | 0.2405 | 0.3031 | 0.0872 | 0.3165 | 0.3023 | 0.3031 | 0.3892 | 0.2061 | 0.1952 | 0.3145 | 0.3172 | 0.3181 | 0.3364 | 0.3186 | 0.3950 |
| S44 | 0.3573 | 0.3665 | 0.2647 | 0.3530 | 0.3520 | 0.3859 | 0.3760 | 0.4162 | 0.3865 | 0.3845 | 0.3304 | 0.3583 | 0.3553 | 0.4111 | 0.4161 | 0.4128 | 0.2665 | 0.4148 | 0.2583 |
| S45 | 0.3276 | 0.3175 | 0.2592 | 0.3070 | 0.3046 | 0.3405 | 0.3400 | 0.3767 | 0.3385 | 0.3396 | 0.3405 | 0.3261 | 0.3155 | 0.3718 | 0.3769 | 0.3733 | 0.1953 | 0.3747 | 0.2593 |
| S46 | 0.3240 | 0.3108 | 0.2629 | 0.3060 | 0.3012 | 0.3330 | 0.3372 | 0.3698 | 0.3319 | 0.3333 | 0.3411 | 0.3227 | 0.3116 | 0.3653 | 0.3721 | 0.3689 | 0.1971 | 0.3708 | 0.2565 |
| S47 | 0.3699 | 0.3756 | 0.2498 | 0.3596 | 0.3570 | 0.3940 | 0.3887 | 0.4261 | 0.3956 | 0.3933 | 0.3327 | 0.3700 | 0.3701 | 0.4222 | 0.4230 | 0.4208 | 0.2651 | 0.4273 | 0.2460 |
| S48 | 0.3662 | 0.3556 | 0.3315 | 0.3464 | 0.3497 | 0.3762 | 0.3943 | 0.4134 | 0.3734 | 0.3771 | 0.2126 | 0.3687 | 0.3715 | 0.4126 | 0.4175 | 0.4116 | 0.3489 | 0.4151 | 0.3339 |
| S49 | 0.3013 | 0.2748 | 0.3959 | 0.2626 | 0.2618 | 0.2965 | 0.3129 | 0.3023 | 0.2965 | 0.2984 | 0.3829 | 0.2970 | 0.2910 | 0.3028 | 0.3013 | 0.3049 | 0.3562 | 0.3037 | 0.3985 |
| S5 | 0.2910 | 0.2592 | 0.3950 | 0.2452 | 0.2403 | 0.2806 | 0.2962 | 0.2943 | 0.2810 | 0.2795 | 0.3806 | 0.2861 | 0.2769 | 0.2905 | 0.2906 | 0.2930 | 0.3475 | 0.2931 | 0.3994 |
| S50 | 0.2862 | 0.2566 | 0.3872 | 0.2385 | 0.2333 | 0.2778 | 0.2904 | 0.2870 | 0.2806 | 0.2786 | 0.3800 | 0.2807 | 0.2691 | 0.2857 | 0.2855 | 0.2848 | 0.3363 | 0.2891 | 0.3883 |
| S51 | 0.3266 | 0.3161 | 0.2580 | 0.3054 | 0.2999 | 0.3340 | 0.3368 | 0.3717 | 0.3301 | 0.3341 | 0.3502 | 0.3237 | 0.3131 | 0.3684 | 0.3698 | 0.3699 | 0.1763 | 0.3705 | 0.2602 |
| S52 | 0.3050 | 0.2853 | 0.2918 | 0.2752 | 0.2743 | 0.3036 | 0.3173 | 0.3443 | 0.3052 | 0.3046 | 0.3168 | 0.3005 | 0.2874 | 0.3374 | 0.3443 | 0.3426 | 0.2397 | 0.3441 | 0.2975 |
| S53 | 0.3760 | 0.3705 | 0.2153 | 0.3612 | 0.3602 | 0.3864 | 0.3946 | 0.4233 | 0.3870 | 0.3847 | 0.3340 | 0.3768 | 0.3741 | 0.4204 | 0.4217 | 0.4213 | 0.2513 | 0.4257 | 0.1940 |
| S54 | 0.3750 | 0.3709 | 0.2179 | 0.3647 | 0.3609 | 0.3905 | 0.3972 | 0.4275 | 0.3890 | 0.3871 | 0.3380 | 0.3745 | 0.3724 | 0.4211 | 0.4245 | 0.4221 | 0.2375 | 0.4245 | 0.1431 |
| S55 | 0.3142 | 0.2961 | 0.2878 | 0.2910 | 0.2855 | 0.3139 | 0.3254 | 0.3546 | 0.3156 | 0.3137 | 0.3197 | 0.3105 | 0.2982 | 0.3498 | 0.3523 | 0.3515 | 0.2356 | 0.3532 | 0.2912 |
| S56 | 0.3695 | 0.3742 | 0.2480 | 0.3597 | 0.3572 | 0.3905 | 0.3891 | 0.4251 | 0.3919 | 0.3904 | 0.3336 | 0.3673 | 0.3661 | 0.4202 | 0.4233 | 0.4216 | 0.2609 | 0.4259 | 0.2429 |
| S57 | 0.2024 | 0.2660 | 0.3828 | 0.2363 | 0.2287 | 0.2949 | 0.1308 | 0.3080 | 0.2927 | 0.2936 | 0.3818 | 0.1992 | 0.1860 | 0.3010 | 0.3055 | 0.3043 | 0.3301 | 0.3074 | 0.3892 |
| S58 | 0.1426 | 0.2508 | 0.3642 | 0.2257 | 0.2205 | 0.2725 | 0.2097 | 0.3009 | 0.2708 | 0.2725 | 0.3597 | 0.1416 | 0.1341 | 0.2958 | 0.3004 | 0.2987 | 0.3139 | 0.3003 | 0.3677 |
| S59 | 0.0000 | 0.2685 | 0.3707 | 0.2391 | 0.2360 | 0.2928 | 0.2115 | 0.3128 | 0.2941 | 0.2921 | 0.3614 | 0.1312 | 0.1302 | 0.3076 | 0.3117 | 0.3100 | 0.3288 | 0.3132 | 0.3753 |
| S6 | 0.2685 | 0.0000 | 0.3669 | 0.2289 | 0.2241 | 0.2045 | 0.2794 | 0.2901 | 0.2053 | 0.2053 | 0.3539 | 0.2643 | 0.2533 | 0.2865 | 0.2902 | 0.2872 | 0.3181 | 0.2893 | 0.3707 |
| S60 | 0.3707 | 0.3669 | 0.0000 | 0.3564 | 0.3569 | 0.3833 | 0.3922 | 0.4216 | 0.3811 | 0.3823 | 0.3298 | 0.3698 | 0.3639 | 0.4180 | 0.4176 | 0.4168 | 0.2467 | 0.4204 | 0.2198 |
| S61 | 0.2391 | 0.2289 | 0.3564 | 0.0000 | 0.1287 | 0.2558 | 0.2471 | 0.2657 | 0.2548 | 0.2559 | 0.3435 | 0.2365 | 0.2233 | 0.2624 | 0.2666 | 0.2655 | 0.3073 | 0.2670 | 0.3613 |
| S62 | 0.2360 | 0.2241 | 0.3569 | 0.1287 | 0.0000 | 0.2505 | 0.2419 | 0.2645 | 0.2500 | 0.2499 | 0.3481 | 0.2317 | 0.2206 | 0.2595 | 0.2603 | 0.2594 | 0.3038 | 0.2636 | 0.3594 |
| S63 | 0.2928 | 0.2045 | 0.3833 | 0.2558 | 0.2505 | 0.0000 | 0.3044 | 0.3160 | 0.1248 | 0.1143 | 0.3740 | 0.2917 | 0.2806 | 0.3134 | 0.3110 | 0.3136 | 0.3347 | 0.3149 | 0.3888 |
| S65 | 0.2115 | 0.2794 | 0.3922 | 0.2471 | 0.2419 | 0.3044 | 0.0000 | 0.3192 | 0.3061 | 0.3064 | 0.3920 | 0.2063 | 0.1977 | 0.3160 | 0.3168 | 0.3183 | 0.3387 | 0.3211 | 0.3954 |
| S7 | 0.3128 | 0.2901 | 0.4216 | 0.2657 | 0.2645 | 0.3160 | 0.3192 | 0.0000 | 0.3167 | 0.3172 | 0.4123 | 0.3091 | 0.2972 | 0.1691 | 0.1690 | 0.1685 | 0.3741 | 0.1419 | 0.4240 |
| S71 | 0.2941 | 0.2053 | 0.3811 | 0.2548 | 0.2500 | 0.1248 | 0.3061 | 0.3167 | 0.0000 | 0.1278 | 0.3713 | 0.2895 | 0.2792 | 0.3123 | 0.3124 | 0.3122 | 0.3347 | 0.3157 | 0.3896 |
| S72 | 0.2921 | 0.2053 | 0.3823 | 0.2559 | 0.2499 | 0.1143 | 0.3064 | 0.3172 | 0.1278 | 0.0000 | 0.3755 | 0.2922 | 0.2801 | 0.3129 | 0.3120 | 0.3144 | 0.3336 | 0.3179 | 0.3879 |
| S73 | 0.3614 | 0.3539 | 0.3298 | 0.3435 | 0.3481 | 0.3740 | 0.3920 | 0.4123 | 0.3713 | 0.3755 | 0.0000 | 0.3651 | 0.3652 | 0.4102 | 0.4127 | 0.4087 | 0.3465 | 0.4123 | 0.3373 |
| S74 | 0.1312 | 0.2643 | 0.3698 | 0.2365 | 0.2317 | 0.2917 | 0.2063 | 0.3091 | 0.2895 | 0.2922 | 0.3651 | 0.0000 | 0.1024 | 0.3044 | 0.3094 | 0.3046 | 0.3251 | 0.3065 | 0.3744 |
| S75 | 0.1302 | 0.2533 | 0.3639 | 0.2233 | 0.2206 | 0.2806 | 0.1977 | 0.2972 | 0.2792 | 0.2801 | 0.3652 | 0.1024 | 0.0000 | 0.2931 | 0.2971 | 0.2953 | 0.3159 | 0.2977 | 0.3717 |
| S76 | 0.3076 | 0.2865 | 0.4180 | 0.2624 | 0.2595 | 0.3134 | 0.3160 | 0.1691 | 0.3123 | 0.3129 | 0.4102 | 0.3044 | 0.2931 | 0.0000 | 0.1675 | 0.1282 | 0.3706 | 0.1660 | 0.4200 |
| S77 | 0.3117 | 0.2902 | 0.4176 | 0.2666 | 0.2603 | 0.3110 | 0.3168 | 0.1690 | 0.3124 | 0.3120 | 0.4127 | 0.3094 | 0.2971 | 0.1675 | 0.0000 | 0.1633 | 0.3730 | 0.1698 | 0.4237 |
| S78 | 0.3100 | 0.2872 | 0.4168 | 0.2655 | 0.2594 | 0.3136 | 0.3183 | 0.1685 | 0.3122 | 0.3144 | 0.4087 | 0.3046 | 0.2953 | 0.1282 | 0.1633 | 0.0000 | 0.3709 | 0.1677 | 0.4212 |
| S79 | 0.3288 | 0.3181 | 0.2467 | 0.3073 | 0.3038 | 0.3347 | 0.3387 | 0.3741 | 0.3347 | 0.3336 | 0.3465 | 0.3251 | 0.3159 | 0.3706 | 0.3730 | 0.3709 | 0.0000 | 0.3735 | 0.2421 |
| S8 | 0.3132 | 0.2893 | 0.4204 | 0.2670 | 0.2636 | 0.3149 | 0.3211 | 0.1419 | 0.3157 | 0.3179 | 0.4123 | 0.3065 | 0.2977 | 0.1660 | 0.1698 | 0.1677 | 0.3735 | 0.0000 | 0.4228 |
| S80 | 0.3753 | 0.3707 | 0.2198 | 0.3613 | 0.3594 | 0.3888 | 0.3954 | 0.4240 | 0.3896 | 0.3879 | 0.3373 | 0.3744 | 0.3717 | 0.4200 | 0.4237 | 0.4212 | 0.2421 | 0.4228 | 0.0000 |
| S84 | 0.3076 | 0.2945 | 0.2856 | 0.2841 | 0.2794 | 0.3105 | 0.3228 | 0.3527 | 0.3140 | 0.3126 | 0.3136 | 0.3065 | 0.2945 | 0.3446 | 0.3527 | 0.3491 | 0.2342 | 0.3500 | 0.2897 |
| S85 | 0.2913 | 0.2018 | 0.3812 | 0.2487 | 0.2456 | 0.1436 | 0.3007 | 0.3108 | 0.1447 | 0.1442 | 0.3711 | 0.2879 | 0.2752 | 0.3056 | 0.3079 | 0.3078 | 0.3315 | 0.3106 | 0.3853 |
| S86 | 0.2982 | 0.2898 | 0.2814 | 0.2718 | 0.2661 | 0.3075 | 0.3087 | 0.3403 | 0.3085 | 0.3065 | 0.3373 | 0.2964 | 0.2852 | 0.3349 | 0.3383 | 0.3372 | 0.2071 | 0.3424 | 0.2839 |
| S87 | 0.3075 | 0.2922 | 0.3109 | 0.2778 | 0.2787 | 0.3130 | 0.3241 | 0.3442 | 0.3119 | 0.3159 | 0.2798 | 0.3067 | 0.2987 | 0.3423 | 0.3456 | 0.3451 | 0.2632 | 0.3448 | 0.3072 |
| S88 | 0.3304 | 0.2561 | 0.3963 | 0.3054 | 0.3049 | 0.2474 | 0.3560 | 0.3632 | 0.2435 | 0.2459 | 0.3564 | 0.3323 | 0.3317 | 0.3606 | 0.3597 | 0.3618 | 0.3882 | 0.3633 | 0.4039 |
| S89 | 0.1997 | 0.2511 | 0.3673 | 0.2212 | 0.2135 | 0.2746 | 0.1944 | 0.2946 | 0.2750 | 0.2744 | 0.3648 | 0.1975 | 0.1851 | 0.2906 | 0.2939 | 0.2928 | 0.3074 | 0.2993 | 0.3653 |
| S9 | 0.3732 | 0.3715 | 0.2173 | 0.3636 | 0.3635 | 0.3892 | 0.3979 | 0.4257 | 0.3875 | 0.3863 | 0.3378 | 0.3771 | 0.3731 | 0.4225 | 0.4251 | 0.4248 | 0.2419 | 0.4274 | 0.2146 |
| S90 | 0.2449 | 0.2407 | 0.3703 | 0.2076 | 0.1997 | 0.2655 | 0.2464 | 0.2896 | 0.2697 | 0.2660 | 0.3709 | 0.2391 | 0.2236 | 0.2794 | 0.2874 | 0.2865 | 0.3140 | 0.2918 | 0.3762 |
| S91 | 0.2521 | 0.2454 | 0.3738 | 0.2112 | 0.2013 | 0.2691 | 0.2532 | 0.2914 | 0.2729 | 0.2737 | 0.3728 | 0.2461 | 0.2328 | 0.2862 | 0.2888 | 0.2881 | 0.3169 | 0.2923 | 0.3759 |
| S92 | 0.2503 | 0.2448 | 0.3733 | 0.2111 | 0.2080 | 0.2731 | 0.2549 | 0.2916 | 0.2754 | 0.2742 | 0.3710 | 0.2475 | 0.2331 | 0.2849 | 0.2886 | 0.2883 | 0.3164 | 0.2916 | 0.3767 |
| S93 | 0.2512 | 0.2439 | 0.3705 | 0.2104 | 0.2019 | 0.2690 | 0.2528 | 0.2902 | 0.2708 | 0.2698 | 0.3677 | 0.2465 | 0.2304 | 0.2836 | 0.2843 | 0.2868 | 0.3158 | 0.2904 | 0.3742 |
| S95 | 0.2567 | 0.2499 | 0.3731 | 0.2165 | 0.2088 | 0.2723 | 0.2606 | 0.2967 | 0.2756 | 0.2742 | 0.3720 | 0.2523 | 0.2381 | 0.2913 | 0.2935 | 0.2946 | 0.3194 | 0.2979 | 0.3770 |
| S96 | 0.2898 | 0.2626 | 0.3953 | 0.2434 | 0.2411 | 0.2826 | 0.2970 | 0.2962 | 0.2826 | 0.2813 | 0.3834 | 0.2859 | 0.2746 | 0.2937 | 0.2933 | 0.2961 | 0.3461 | 0.2969 | 0.4003 |
| S97 | 0.3604 | 0.3458 | 0.3394 | 0.3415 | 0.3443 | 0.3646 | 0.3878 | 0.4089 | 0.3642 | 0.3660 | 0.2339 | 0.3620 | 0.3627 | 0.4062 | 0.4094 | 0.4054 | 0.3492 | 0.4085 | 0.3368 |
| S98 | 0.2913 | 0.2607 | 0.3943 | 0.2450 | 0.2385 | 0.2799 | 0.2970 | 0.2940 | 0.2809 | 0.2807 | 0.3850 | 0.2858 | 0.2745 | 0.2894 | 0.2908 | 0.2922 | 0.3463 | 0.2959 | 0.3972 |
| S99 | 0.2935 | 0.2614 | 0.3965 | 0.2458 | 0.2427 | 0.2803 | 0.2996 | 0.2946 | 0.2785 | 0.2802 | 0.3837 | 0.2879 | 0.2793 | 0.2935 | 0.2932 | 0.2941 | 0.3472 | 0.2974 | 0.3996 |

Table S4 (Continued)

| Sample ID | S84 | S85 | S86 | S87 | S88 | S89 | S9 | S90 | S91 | S92 | S93 | S95 | S96 | S97 | S98 | S99 |
| --- | --- | --- | --- | --- | --- | --- | --- | --- | --- | --- | --- | --- | --- | --- | --- | --- |
| S1 | 0.3226 | 0.3734 | 0.3430 | 0.2821 | 0.3516 | 0.3690 | 0.3389 | 0.3785 | 0.3769 | 0.3752 | 0.3769 | 0.3776 | 0.3894 | 0.2270 | 0.3917 | 0.3876 |
| S10 | 0.2375 | 0.3375 | 0.2016 | 0.2641 | 0.3921 | 0.3147 | 0.2482 | 0.3201 | 0.3246 | 0.3228 | 0.3228 | 0.3283 | 0.3501 | 0.3505 | 0.3484 | 0.3501 |
| S101 | 0.3307 | 0.1379 | 0.3269 | 0.3291 | 0.2369 | 0.2946 | 0.3987 | 0.2870 | 0.2878 | 0.2895 | 0.2852 | 0.2898 | 0.2873 | 0.3537 | 0.2863 | 0.2857 |
| S102 | 0.3243 | 0.1347 | 0.3208 | 0.3225 | 0.2376 | 0.2879 | 0.4000 | 0.2761 | 0.2777 | 0.2828 | 0.2751 | 0.2821 | 0.2812 | 0.3527 | 0.2826 | 0.2814 |
| S103 | 0.3687 | 0.3295 | 0.3618 | 0.3655 | 0.3685 | 0.3139 | 0.4405 | 0.3091 | 0.3130 | 0.3111 | 0.3044 | 0.3164 | 0.3056 | 0.4062 | 0.3008 | 0.3042 |
| S104 | 0.3169 | 0.2096 | 0.3102 | 0.3090 | 0.2428 | 0.2703 | 0.3847 | 0.2657 | 0.2657 | 0.2687 | 0.2641 | 0.2718 | 0.2672 | 0.3398 | 0.2658 | 0.2649 |
| S105 | 0.3496 | 0.3091 | 0.3394 | 0.3478 | 0.3610 | 0.2945 | 0.4260 | 0.2874 | 0.2879 | 0.2874 | 0.2856 | 0.2958 | 0.2859 | 0.3961 | 0.2828 | 0.2862 |
| S11 | 0.3194 | 0.2978 | 0.3057 | 0.3195 | 0.3563 | 0.1936 | 0.3948 | 0.2438 | 0.2493 | 0.2508 | 0.2485 | 0.2563 | 0.2955 | 0.3872 | 0.2953 | 0.2970 |
| S12 | 0.3026 | 0.3878 | 0.2910 | 0.3185 | 0.3960 | 0.3691 | 0.2545 | 0.3769 | 0.3764 | 0.3734 | 0.3735 | 0.3776 | 0.3922 | 0.3352 | 0.3891 | 0.3951 |
| S13 | 0.3190 | 0.2986 | 0.3068 | 0.3203 | 0.3550 | 0.1902 | 0.3979 | 0.2419 | 0.2500 | 0.2502 | 0.2503 | 0.2560 | 0.2940 | 0.3869 | 0.2927 | 0.2965 |
| S14 | 0.2943 | 0.2581 | 0.2881 | 0.2864 | 0.3025 | 0.2351 | 0.3698 | 0.2229 | 0.2297 | 0.2275 | 0.2271 | 0.2323 | 0.2542 | 0.3408 | 0.2540 | 0.2531 |
| S15 | 0.3517 | 0.3127 | 0.3416 | 0.3496 | 0.3626 | 0.2957 | 0.4271 | 0.2888 | 0.2937 | 0.2893 | 0.2897 | 0.3002 | 0.2981 | 0.4098 | 0.2927 | 0.2972 |
| S16 | 0.3939 | 0.3547 | 0.3818 | 0.3838 | 0.3685 | 0.3411 | 0.4444 | 0.3334 | 0.3360 | 0.3374 | 0.3350 | 0.3395 | 0.3252 | 0.4212 | 0.3246 | 0.3242 |
| S17 | 0.3927 | 0.3506 | 0.3839 | 0.3821 | 0.3678 | 0.3412 | 0.4416 | 0.3348 | 0.3306 | 0.3332 | 0.3333 | 0.3391 | 0.3288 | 0.4196 | 0.3261 | 0.3288 |
| S18 | 0.3924 | 0.3540 | 0.3854 | 0.3840 | 0.3674 | 0.3427 | 0.4458 | 0.3359 | 0.3388 | 0.3376 | 0.3366 | 0.3445 | 0.3282 | 0.4200 | 0.3270 | 0.3306 |
| S19 | 0.3535 | 0.3127 | 0.3412 | 0.3482 | 0.3624 | 0.2936 | 0.4258 | 0.2889 | 0.2915 | 0.2892 | 0.2875 | 0.2968 | 0.2952 | 0.4104 | 0.2933 | 0.2972 |
| S2 | 0.3037 | 0.1821 | 0.3010 | 0.2993 | 0.2428 | 0.2657 | 0.3814 | 0.2584 | 0.2625 | 0.2617 | 0.2606 | 0.2671 | 0.2713 | 0.3484 | 0.2688 | 0.2701 |
| S20 | 0.3574 | 0.3189 | 0.3442 | 0.3530 | 0.3666 | 0.2979 | 0.4289 | 0.2900 | 0.2942 | 0.2920 | 0.2898 | 0.3017 | 0.3027 | 0.4135 | 0.2969 | 0.3032 |
| S21 | 0.3197 | 0.3685 | 0.3393 | 0.2743 | 0.3565 | 0.3687 | 0.3399 | 0.3733 | 0.3724 | 0.3717 | 0.3698 | 0.3727 | 0.3858 | 0.2259 | 0.3866 | 0.3863 |
| S22 | 0.2986 | 0.3769 | 0.2662 | 0.3115 | 0.3920 | 0.3450 | 0.2555 | 0.3622 | 0.3592 | 0.3601 | 0.3591 | 0.3632 | 0.3856 | 0.3342 | 0.3847 | 0.3860 |
| S23 | 0.2405 | 0.3343 | 0.2091 | 0.2598 | 0.3866 | 0.3071 | 0.2530 | 0.3146 | 0.3234 | 0.3214 | 0.3192 | 0.3231 | 0.3490 | 0.3427 | 0.3445 | 0.3484 |
| S24 | 0.2185 | 0.3258 | 0.2069 | 0.2527 | 0.3816 | 0.3035 | 0.2557 | 0.3052 | 0.3130 | 0.3085 | 0.3114 | 0.3147 | 0.3400 | 0.3448 | 0.3382 | 0.3430 |
| S26 | 0.3389 | 0.2232 | 0.3357 | 0.3279 | 0.2272 | 0.3009 | 0.3862 | 0.2932 | 0.2985 | 0.2964 | 0.2951 | 0.3016 | 0.3032 | 0.3347 | 0.3012 | 0.3021 |
| S27 | 0.3271 | 0.3149 | 0.3110 | 0.3208 | 0.3290 | 0.2727 | 0.3562 | 0.2829 | 0.2848 | 0.2859 | 0.2821 | 0.2897 | 0.3156 | 0.3528 | 0.3149 | 0.3177 |
| S28 | 0.2845 | 0.2448 | 0.2686 | 0.2811 | 0.3022 | 0.1947 | 0.3657 | 0.1964 | 0.1987 | 0.2003 | 0.2002 | 0.2050 | 0.2423 | 0.3454 | 0.2416 | 0.2425 |
| S29 | 0.3508 | 0.3068 | 0.3342 | 0.3437 | 0.3579 | 0.2871 | 0.4207 | 0.2823 | 0.2848 | 0.2845 | 0.2803 | 0.2885 | 0.2903 | 0.4054 | 0.2848 | 0.2900 |
| S3 | 0.3169 | 0.1906 | 0.3129 | 0.3114 | 0.2384 | 0.2784 | 0.3884 | 0.2703 | 0.2762 | 0.2754 | 0.2721 | 0.2786 | 0.2794 | 0.3461 | 0.2779 | 0.2793 |
| S30 | 0.4064 | 0.4156 | 0.3738 | 0.4002 | 0.4015 | 0.4007 | 0.3937 | 0.4068 | 0.4049 | 0.4039 | 0.4009 | 0.4050 | 0.4038 | 0.3951 | 0.4030 | 0.4055 |
| S31 | 0.3518 | 0.3137 | 0.3387 | 0.3481 | 0.3612 | 0.2902 | 0.4243 | 0.2865 | 0.2897 | 0.2875 | 0.2864 | 0.2938 | 0.2973 | 0.4099 | 0.2892 | 0.2941 |
| S32 | 0.2219 | 0.3232 | 0.1984 | 0.2539 | 0.3829 | 0.2979 | 0.2580 | 0.3033 | 0.3085 | 0.3054 | 0.3070 | 0.3128 | 0.3367 | 0.3436 | 0.3345 | 0.3377 |
| S33 | 0.3566 | 0.3126 | 0.3395 | 0.3502 | 0.3628 | 0.2957 | 0.4290 | 0.2907 | 0.2926 | 0.2903 | 0.2910 | 0.2979 | 0.2966 | 0.4136 | 0.2918 | 0.2980 |
| S34 | 0.1371 | 0.3123 | 0.2317 | 0.2235 | 0.3673 | 0.2937 | 0.2962 | 0.2937 | 0.3015 | 0.2997 | 0.3002 | 0.3041 | 0.3273 | 0.3171 | 0.3247 | 0.3256 |
| S35 | 0.3785 | 0.3785 | 0.3632 | 0.3793 | 0.3937 | 0.3593 | 0.4025 | 0.3630 | 0.3660 | 0.3630 | 0.3606 | 0.3695 | 0.3686 | 0.4147 | 0.3631 | 0.3695 |
| S36 | 0.2927 | 0.2718 | 0.2773 | 0.2917 | 0.3345 | 0.0560 | 0.3683 | 0.2186 | 0.2288 | 0.2259 | 0.2241 | 0.2304 | 0.2735 | 0.3636 | 0.2716 | 0.2752 |
| S37 | 0.3127 | 0.2666 | 0.3015 | 0.3123 | 0.3234 | 0.2632 | 0.3931 | 0.2498 | 0.2548 | 0.2567 | 0.2523 | 0.2597 | 0.1599 | 0.3752 | 0.1609 | 0.1623 |
| S38 | 0.3846 | 0.4173 | 0.3429 | 0.3826 | 0.4084 | 0.4044 | 0.3546 | 0.4128 | 0.4105 | 0.4062 | 0.4066 | 0.4115 | 0.4103 | 0.3773 | 0.4071 | 0.4115 |
| S39 | 0.3210 | 0.2724 | 0.3086 | 0.3147 | 0.3263 | 0.2675 | 0.4001 | 0.2568 | 0.2635 | 0.2652 | 0.2555 | 0.2672 | 0.1364 | 0.3765 | 0.1397 | 0.1393 |
| S4 | 0.2970 | 0.1999 | 0.2907 | 0.2936 | 0.2545 | 0.2500 | 0.3746 | 0.2449 | 0.2463 | 0.2475 | 0.2447 | 0.2510 | 0.2607 | 0.3479 | 0.2591 | 0.2612 |
| S40 | 0.2099 | 0.3034 | 0.2377 | 0.1532 | 0.3548 | 0.2868 | 0.3130 | 0.2852 | 0.2909 | 0.2891 | 0.2900 | 0.2952 | 0.3167 | 0.2866 | 0.3156 | 0.3187 |
| S41 | 0.2101 | 0.3052 | 0.2379 | 0.1519 | 0.3569 | 0.2859 | 0.3100 | 0.2834 | 0.2920 | 0.2897 | 0.2885 | 0.2943 | 0.3155 | 0.2887 | 0.3156 | 0.3168 |
| S42 | 0.3211 | 0.2995 | 0.3081 | 0.3211 | 0.3549 | 0.1918 | 0.3962 | 0.2450 | 0.2518 | 0.2526 | 0.2503 | 0.2566 | 0.2949 | 0.3893 | 0.2942 | 0.2978 |
| S44 | 0.3039 | 0.3863 | 0.2734 | 0.3158 | 0.3955 | 0.3518 | 0.2552 | 0.3661 | 0.3684 | 0.3651 | 0.3635 | 0.3711 | 0.3924 | 0.3339 | 0.3928 | 0.3937 |
| S45 | 0.2422 | 0.3355 | 0.2075 | 0.2607 | 0.3859 | 0.3102 | 0.2512 | 0.3164 | 0.3218 | 0.3206 | 0.3194 | 0.3258 | 0.3507 | 0.3419 | 0.3482 | 0.3520 |
| S46 | 0.2407 | 0.3313 | 0.2072 | 0.2592 | 0.3848 | 0.3072 | 0.2573 | 0.3122 | 0.3199 | 0.3165 | 0.3163 | 0.3222 | 0.3470 | 0.3456 | 0.3466 | 0.3465 |
| S47 | 0.3043 | 0.3934 | 0.2835 | 0.3212 | 0.4026 | 0.3652 | 0.2412 | 0.3787 | 0.3805 | 0.3756 | 0.3746 | 0.3791 | 0.4014 | 0.3383 | 0.4001 | 0.4020 |
| S48 | 0.3196 | 0.3729 | 0.3403 | 0.2783 | 0.3534 | 0.3712 | 0.3392 | 0.3743 | 0.3738 | 0.3743 | 0.3741 | 0.3758 | 0.3876 | 0.2235 | 0.3882 | 0.3878 |
| S49 | 0.3358 | 0.2911 | 0.3281 | 0.3269 | 0.3294 | 0.2904 | 0.4023 | 0.2813 | 0.2817 | 0.2822 | 0.2791 | 0.2879 | 0.1817 | 0.3799 | 0.1821 | 0.1831 |
| S5 | 0.3242 | 0.2738 | 0.3097 | 0.3171 | 0.3276 | 0.2697 | 0.3997 | 0.2596 | 0.2645 | 0.2654 | 0.2596 | 0.2704 | 0.1463 | 0.3811 | 0.1455 | 0.1450 |
| S50 | 0.3136 | 0.2734 | 0.3032 | 0.3098 | 0.3268 | 0.2616 | 0.3932 | 0.2528 | 0.2556 | 0.2566 | 0.2526 | 0.2583 | 0.1646 | 0.3756 | 0.1633 | 0.1675 |
| S51 | 0.2334 | 0.3318 | 0.1913 | 0.2619 | 0.3873 | 0.3054 | 0.2551 | 0.3107 | 0.3159 | 0.3116 | 0.3120 | 0.3197 | 0.3441 | 0.3493 | 0.3432 | 0.3450 |
| S52 | 0.1540 | 0.3016 | 0.2215 | 0.2240 | 0.3607 | 0.2835 | 0.3004 | 0.2835 | 0.2870 | 0.2865 | 0.2884 | 0.2898 | 0.3174 | 0.3181 | 0.3140 | 0.3164 |
| S53 | 0.2860 | 0.3852 | 0.2856 | 0.3119 | 0.3998 | 0.3685 | 0.2143 | 0.3768 | 0.3778 | 0.3758 | 0.3767 | 0.3791 | 0.4003 | 0.3380 | 0.4010 | 0.4011 |
| S54 | 0.2936 | 0.3875 | 0.2785 | 0.3144 | 0.4023 | 0.3676 | 0.2052 | 0.3799 | 0.3777 | 0.3783 | 0.3753 | 0.3787 | 0.4017 | 0.3404 | 0.3994 | 0.4019 |
| S55 | 0.1496 | 0.3111 | 0.2311 | 0.2319 | 0.3685 | 0.2960 | 0.2940 | 0.2955 | 0.2993 | 0.2983 | 0.2986 | 0.3027 | 0.3283 | 0.3226 | 0.3262 | 0.3273 |
| S56 | 0.3037 | 0.3915 | 0.2822 | 0.3214 | 0.4024 | 0.3646 | 0.2426 | 0.3758 | 0.3767 | 0.3762 | 0.3743 | 0.3801 | 0.3993 | 0.3379 | 0.3970 | 0.3991 |
| S57 | 0.3098 | 0.2887 | 0.2961 | 0.3089 | 0.3455 | 0.1914 | 0.3879 | 0.2339 | 0.2387 | 0.2399 | 0.2401 | 0.2465 | 0.2822 | 0.3779 | 0.2814 | 0.2875 |
| S58 | 0.2935 | 0.2672 | 0.2848 | 0.2948 | 0.3229 | 0.1921 | 0.3720 | 0.2331 | 0.2352 | 0.2400 | 0.2372 | 0.2425 | 0.2767 | 0.3563 | 0.2769 | 0.2791 |
| S59 | 0.3076 | 0.2913 | 0.2982 | 0.3075 | 0.3304 | 0.1997 | 0.3732 | 0.2449 | 0.2521 | 0.2503 | 0.2512 | 0.2567 | 0.2898 | 0.3604 | 0.2913 | 0.2935 |
| S6 | 0.2945 | 0.2018 | 0.2898 | 0.2922 | 0.2561 | 0.2511 | 0.3715 | 0.2407 | 0.2454 | 0.2448 | 0.2439 | 0.2499 | 0.2626 | 0.3458 | 0.2607 | 0.2614 |
| S60 | 0.2856 | 0.3812 | 0.2814 | 0.3109 | 0.3963 | 0.3673 | 0.2173 | 0.3703 | 0.3738 | 0.3733 | 0.3705 | 0.3731 | 0.3953 | 0.3394 | 0.3943 | 0.3965 |
| S61 | 0.2841 | 0.2487 | 0.2718 | 0.2778 | 0.3054 | 0.2212 | 0.3636 | 0.2076 | 0.2112 | 0.2111 | 0.2104 | 0.2165 | 0.2434 | 0.3415 | 0.2450 | 0.2458 |
| S62 | 0.2794 | 0.2456 | 0.2661 | 0.2787 | 0.3049 | 0.2135 | 0.3635 | 0.1997 | 0.2013 | 0.2080 | 0.2019 | 0.2088 | 0.2411 | 0.3443 | 0.2385 | 0.2427 |
| S63 | 0.3105 | 0.1436 | 0.3075 | 0.3130 | 0.2474 | 0.2746 | 0.3892 | 0.2655 | 0.2691 | 0.2731 | 0.2690 | 0.2723 | 0.2826 | 0.3646 | 0.2799 | 0.2803 |
| S65 | 0.3228 | 0.3007 | 0.3087 | 0.3241 | 0.3560 | 0.1944 | 0.3979 | 0.2464 | 0.2532 | 0.2549 | 0.2528 | 0.2606 | 0.2970 | 0.3878 | 0.2970 | 0.2996 |
| S7 | 0.3527 | 0.3108 | 0.3403 | 0.3442 | 0.3632 | 0.2946 | 0.4257 | 0.2896 | 0.2914 | 0.2916 | 0.2902 | 0.2967 | 0.2962 | 0.4089 | 0.2940 | 0.2946 |
| S71 | 0.3140 | 0.1447 | 0.3085 | 0.3119 | 0.2435 | 0.2750 | 0.3875 | 0.2697 | 0.2729 | 0.2754 | 0.2708 | 0.2756 | 0.2826 | 0.3642 | 0.2809 | 0.2785 |
| S72 | 0.3126 | 0.1442 | 0.3065 | 0.3159 | 0.2459 | 0.2744 | 0.3863 | 0.2660 | 0.2737 | 0.2742 | 0.2698 | 0.2742 | 0.2813 | 0.3660 | 0.2807 | 0.2802 |
| S73 | 0.3136 | 0.3711 | 0.3373 | 0.2798 | 0.3564 | 0.3648 | 0.3378 | 0.3709 | 0.3728 | 0.3710 | 0.3677 | 0.3720 | 0.3834 | 0.2339 | 0.3850 | 0.3837 |
| S74 | 0.3065 | 0.2879 | 0.2964 | 0.3067 | 0.3323 | 0.1975 | 0.3771 | 0.2391 | 0.2461 | 0.2475 | 0.2465 | 0.2523 | 0.2859 | 0.3620 | 0.2858 | 0.2879 |
| S75 | 0.2945 | 0.2752 | 0.2852 | 0.2987 | 0.3317 | 0.1851 | 0.3731 | 0.2236 | 0.2328 | 0.2331 | 0.2304 | 0.2381 | 0.2746 | 0.3627 | 0.2745 | 0.2793 |
| S76 | 0.3446 | 0.3056 | 0.3349 | 0.3423 | 0.3606 | 0.2906 | 0.4225 | 0.2794 | 0.2862 | 0.2849 | 0.2836 | 0.2913 | 0.2937 | 0.4062 | 0.2894 | 0.2935 |
| S77 | 0.3527 | 0.3079 | 0.3383 | 0.3456 | 0.3597 | 0.2939 | 0.4251 | 0.2874 | 0.2888 | 0.2886 | 0.2843 | 0.2935 | 0.2933 | 0.4094 | 0.2908 | 0.2932 |
| S78 | 0.3491 | 0.3078 | 0.3372 | 0.3451 | 0.3618 | 0.2928 | 0.4248 | 0.2865 | 0.2881 | 0.2883 | 0.2868 | 0.2946 | 0.2961 | 0.4054 | 0.2922 | 0.2941 |
| S79 | 0.2342 | 0.3315 | 0.2071 | 0.2632 | 0.3882 | 0.3074 | 0.2419 | 0.3140 | 0.3169 | 0.3164 | 0.3158 | 0.3194 | 0.3461 | 0.3492 | 0.3463 | 0.3472 |
| S8 | 0.3500 | 0.3106 | 0.3424 | 0.3448 | 0.3633 | 0.2993 | 0.4274 | 0.2918 | 0.2923 | 0.2916 | 0.2904 | 0.2979 | 0.2969 | 0.4085 | 0.2959 | 0.2974 |
| S80 | 0.2897 | 0.3853 | 0.2839 | 0.3072 | 0.4039 | 0.3653 | 0.2146 | 0.3762 | 0.3759 | 0.3767 | 0.3742 | 0.3770 | 0.4003 | 0.3368 | 0.3972 | 0.3996 |
| S84 | 0.0000 | 0.3101 | 0.2276 | 0.2226 | 0.3684 | 0.2912 | 0.2941 | 0.2880 | 0.2948 | 0.2894 | 0.2950 | 0.2973 | 0.3227 | 0.3175 | 0.3201 | 0.3234 |
| S85 | 0.3101 | 0.0000 | 0.3037 | 0.3060 | 0.2403 | 0.2716 | 0.3902 | 0.2595 | 0.2631 | 0.2649 | 0.2618 | 0.2670 | 0.2748 | 0.3601 | 0.2755 | 0.2770 |
| S86 | 0.2276 | 0.3037 | 0.0000 | 0.2472 | 0.3671 | 0.2762 | 0.2818 | 0.2806 | 0.2845 | 0.2804 | 0.2829 | 0.2853 | 0.3109 | 0.3365 | 0.3075 | 0.3115 |
| S87 | 0.2226 | 0.3060 | 0.2472 | 0.0000 | 0.3560 | 0.2931 | 0.3143 | 0.2917 | 0.2970 | 0.2981 | 0.2945 | 0.3011 | 0.3172 | 0.2835 | 0.3169 | 0.3181 |
| S88 | 0.3684 | 0.2403 | 0.3671 | 0.3560 | 0.0000 | 0.3338 | 0.4033 | 0.3296 | 0.3307 | 0.3318 | 0.3271 | 0.3333 | 0.3322 | 0.3499 | 0.3324 | 0.3301 |
| S89 | 0.2912 | 0.2716 | 0.2762 | 0.2931 | 0.3338 | 0.0000 | 0.3705 | 0.2182 | 0.2272 | 0.2243 | 0.2228 | 0.2278 | 0.2704 | 0.3604 | 0.2695 | 0.2729 |
| S9 | 0.2941 | 0.3902 | 0.2818 | 0.3143 | 0.4033 | 0.3705 | 0.0000 | 0.3787 | 0.3803 | 0.3780 | 0.3772 | 0.3814 | 0.4010 | 0.3444 | 0.4011 | 0.4030 |
| S90 | 0.2880 | 0.2595 | 0.2806 | 0.2917 | 0.3296 | 0.2182 | 0.3787 | 0.0000 | 0.1148 | 0.1176 | 0.1157 | 0.1212 | 0.2585 | 0.3639 | 0.2554 | 0.2591 |
| S91 | 0.2948 | 0.2631 | 0.2845 | 0.2970 | 0.3307 | 0.2272 | 0.3803 | 0.1148 | 0.0000 | 0.1182 | 0.1103 | 0.0891 | 0.2595 | 0.3637 | 0.2571 | 0.2615 |
| S92 | 0.2894 | 0.2649 | 0.2804 | 0.2981 | 0.3318 | 0.2243 | 0.3780 | 0.1176 | 0.1182 | 0.0000 | 0.1220 | 0.1263 | 0.2630 | 0.3629 | 0.2608 | 0.2657 |
| S93 | 0.2950 | 0.2618 | 0.2829 | 0.2945 | 0.3271 | 0.2228 | 0.3772 | 0.1157 | 0.1103 | 0.1220 | 0.0000 | 0.1165 | 0.2542 | 0.3604 | 0.2539 | 0.2570 |
| S95 | 0.2973 | 0.2670 | 0.2853 | 0.3011 | 0.3333 | 0.2278 | 0.3814 | 0.1212 | 0.0891 | 0.1263 | 0.1165 | 0.0000 | 0.2655 | 0.3658 | 0.2637 | 0.2694 |
| S96 | 0.3227 | 0.2748 | 0.3109 | 0.3172 | 0.3322 | 0.2704 | 0.4010 | 0.2585 | 0.2595 | 0.2630 | 0.2542 | 0.2655 | 0.0000 | 0.3698 | 0.1051 | 0.1111 |
| S97 | 0.3175 | 0.3601 | 0.3365 | 0.2835 | 0.3499 | 0.3604 | 0.3444 | 0.3639 | 0.3637 | 0.3629 | 0.3604 | 0.3658 | 0.3698 | 0.0000 | 0.3684 | 0.3661 |
| S98 | 0.3201 | 0.2755 | 0.3075 | 0.3169 | 0.3324 | 0.2695 | 0.4011 | 0.2554 | 0.2571 | 0.2608 | 0.2539 | 0.2637 | 0.1051 | 0.3684 | 0.0000 | 0.1092 |
| S99 | 0.3234 | 0.2770 | 0.3115 | 0.3181 | 0.3301 | 0.2729 | 0.4030 | 0.2591 | 0.2615 | 0.2657 | 0.2570 | 0.2694 | 0.1111 | 0.3661 | 0.1092 | 0.0000 |

Table S5 Pairwise phylogenetic distance (patristic distance) matrix among 92 accessions of *Camellia*, based on the rooted maximum-likelihood tree, including 87 focal accessions (sect. *Eriandria* and sect. *Theopsis*) and five outgroup accessions from other sections.

| Sample ID | S1 | S48 | S21 | S73 | S97 | S40 | S41 | S87 | S10 | S51 | S79 | S23 | S45 | S46 | S24 | S32 | S12 | S22 | S44 |
| --- | --- | --- | --- | --- | --- | --- | --- | --- | --- | --- | --- | --- | --- | --- | --- | --- | --- | --- | --- |
| S1 | 0.0000 | 0.0153 | 0.0222 | 0.0231 | 0.0277 | 0.1048 | 0.1113 | 0.1061 | 0.2043 | 0.2119 | 0.1984 | 0.2050 | 0.2060 | 0.2011 | 0.1999 | 0.2026 | 0.1700 | 0.1872 | 0.1858 |
| S48 | 0.0153 | 0.0000 | 0.0220 | 0.0229 | 0.0275 | 0.1045 | 0.1111 | 0.1059 | 0.2041 | 0.2117 | 0.1982 | 0.2048 | 0.2058 | 0.2009 | 0.1996 | 0.2024 | 0.1698 | 0.1869 | 0.1856 |
| S21 | 0.0222 | 0.0220 | 0.0000 | 0.0171 | 0.0275 | 0.1046 | 0.1111 | 0.1059 | 0.2041 | 0.2117 | 0.1982 | 0.2048 | 0.2058 | 0.2009 | 0.1996 | 0.2024 | 0.1698 | 0.1870 | 0.1856 |
| S73 | 0.0231 | 0.0229 | 0.0171 | 0.0000 | 0.0284 | 0.1054 | 0.1120 | 0.1068 | 0.2050 | 0.2126 | 0.1991 | 0.2057 | 0.2067 | 0.2017 | 0.2005 | 0.2033 | 0.1706 | 0.1878 | 0.1865 |
| S97 | 0.0277 | 0.0275 | 0.0275 | 0.0284 | 0.0000 | 0.1019 | 0.1084 | 0.1032 | 0.2015 | 0.2090 | 0.1955 | 0.2021 | 0.2031 | 0.1982 | 0.1970 | 0.1997 | 0.1671 | 0.1843 | 0.1830 |
| S40 | 0.1048 | 0.1045 | 0.1046 | 0.1054 | 0.1019 | 0.0000 | 0.0587 | 0.0535 | 0.2408 | 0.2483 | 0.2348 | 0.2414 | 0.2424 | 0.2375 | 0.2363 | 0.2390 | 0.2064 | 0.2236 | 0.2223 |
| S41 | 0.1113 | 0.1111 | 0.1111 | 0.1120 | 0.1084 | 0.0587 | 0.0000 | 0.0451 | 0.2473 | 0.2549 | 0.2413 | 0.2480 | 0.2490 | 0.2440 | 0.2428 | 0.2456 | 0.2129 | 0.2301 | 0.2288 |
| S87 | 0.1061 | 0.1059 | 0.1059 | 0.1068 | 0.1032 | 0.0535 | 0.0451 | 0.0000 | 0.2421 | 0.2497 | 0.2362 | 0.2428 | 0.2438 | 0.2389 | 0.2376 | 0.2404 | 0.2077 | 0.2249 | 0.2236 |
| S10 | 0.2043 | 0.2041 | 0.2041 | 0.2050 | 0.2015 | 0.2408 | 0.2473 | 0.2421 | 0.0000 | 0.0624 | 0.0785 | 0.1188 | 0.1198 | 0.1149 | 0.1397 | 0.1424 | 0.1324 | 0.1496 | 0.1482 |
| S51 | 0.2119 | 0.2117 | 0.2117 | 0.2126 | 0.2090 | 0.2483 | 0.2549 | 0.2497 | 0.0624 | 0.0000 | 0.0861 | 0.1264 | 0.1274 | 0.1225 | 0.1473 | 0.1500 | 0.1399 | 0.1571 | 0.1558 |
| S79 | 0.1984 | 0.1982 | 0.1982 | 0.1991 | 0.1955 | 0.2348 | 0.2413 | 0.2362 | 0.0785 | 0.0861 | 0.0000 | 0.1129 | 0.1139 | 0.1089 | 0.1337 | 0.1365 | 0.1264 | 0.1436 | 0.1423 |
| S23 | 0.2050 | 0.2048 | 0.2048 | 0.2057 | 0.2021 | 0.2414 | 0.2480 | 0.2428 | 0.1188 | 0.1264 | 0.1129 | 0.0000 | 0.0524 | 0.0673 | 0.1404 | 0.1431 | 0.1330 | 0.1502 | 0.1489 |
| S45 | 0.2060 | 0.2058 | 0.2058 | 0.2067 | 0.2031 | 0.2424 | 0.2490 | 0.2438 | 0.1198 | 0.1274 | 0.1139 | 0.0524 | 0.0000 | 0.0683 | 0.1414 | 0.1441 | 0.1340 | 0.1512 | 0.1499 |
| S46 | 0.2011 | 0.2009 | 0.2009 | 0.2017 | 0.1982 | 0.2375 | 0.2440 | 0.2389 | 0.1149 | 0.1225 | 0.1089 | 0.0673 | 0.0683 | 0.0000 | 0.1364 | 0.1392 | 0.1291 | 0.1463 | 0.1450 |
| S24 | 0.1999 | 0.1996 | 0.1996 | 0.2005 | 0.1970 | 0.2363 | 0.2428 | 0.2376 | 0.1397 | 0.1473 | 0.1337 | 0.1404 | 0.1414 | 0.1364 | 0.0000 | 0.0752 | 0.1279 | 0.1451 | 0.1437 |
| S32 | 0.2026 | 0.2024 | 0.2024 | 0.2033 | 0.1997 | 0.2390 | 0.2456 | 0.2404 | 0.1424 | 0.1500 | 0.1365 | 0.1431 | 0.1441 | 0.1392 | 0.0752 | 0.0000 | 0.1306 | 0.1478 | 0.1465 |
| S12 | 0.1700 | 0.1698 | 0.1698 | 0.1706 | 0.1671 | 0.2064 | 0.2129 | 0.2077 | 0.1324 | 0.1399 | 0.1264 | 0.1330 | 0.1340 | 0.1291 | 0.1279 | 0.1306 | 0.0000 | 0.0715 | 0.0702 |
| S22 | 0.1872 | 0.1869 | 0.1870 | 0.1878 | 0.1843 | 0.2236 | 0.2301 | 0.2249 | 0.1496 | 0.1571 | 0.1436 | 0.1502 | 0.1512 | 0.1463 | 0.1451 | 0.1478 | 0.0715 | 0.0000 | 0.0368 |
| S44 | 0.1858 | 0.1856 | 0.1856 | 0.1865 | 0.1830 | 0.2223 | 0.2288 | 0.2236 | 0.1482 | 0.1558 | 0.1423 | 0.1489 | 0.1499 | 0.1450 | 0.1437 | 0.1465 | 0.0702 | 0.0368 | 0.0000 |
| S47 | 0.1768 | 0.1766 | 0.1766 | 0.1775 | 0.1740 | 0.2133 | 0.2198 | 0.2146 | 0.1392 | 0.1468 | 0.1333 | 0.1399 | 0.1409 | 0.1360 | 0.1348 | 0.1375 | 0.0612 | 0.0560 | 0.0547 |
| S56 | 0.1765 | 0.1763 | 0.1763 | 0.1771 | 0.1736 | 0.2129 | 0.2194 | 0.2142 | 0.1389 | 0.1464 | 0.1329 | 0.1396 | 0.1406 | 0.1356 | 0.1344 | 0.1371 | 0.0609 | 0.0556 | 0.0543 |
| S53 | 0.1766 | 0.1764 | 0.1764 | 0.1773 | 0.1737 | 0.2130 | 0.2195 | 0.2144 | 0.1390 | 0.1466 | 0.1330 | 0.1397 | 0.1407 | 0.1357 | 0.1345 | 0.1373 | 0.0768 | 0.0940 | 0.0927 |
| S60 | 0.1764 | 0.1761 | 0.1761 | 0.1770 | 0.1735 | 0.2128 | 0.2193 | 0.2141 | 0.1387 | 0.1463 | 0.1328 | 0.1394 | 0.1404 | 0.1355 | 0.1343 | 0.1370 | 0.0766 | 0.0938 | 0.0925 |
| S54 | 0.1788 | 0.1786 | 0.1786 | 0.1794 | 0.1759 | 0.2152 | 0.2217 | 0.2165 | 0.1412 | 0.1487 | 0.1352 | 0.1418 | 0.1428 | 0.1379 | 0.1367 | 0.1394 | 0.0790 | 0.0962 | 0.0949 |
| S80 | 0.1780 | 0.1778 | 0.1778 | 0.1786 | 0.1751 | 0.2144 | 0.2209 | 0.2158 | 0.1404 | 0.1480 | 0.1344 | 0.1411 | 0.1421 | 0.1371 | 0.1359 | 0.1386 | 0.0782 | 0.0954 | 0.0941 |
| S9 | 0.1661 | 0.1659 | 0.1659 | 0.1668 | 0.1632 | 0.2025 | 0.2091 | 0.2039 | 0.1285 | 0.1361 | 0.1225 | 0.1292 | 0.1302 | 0.1252 | 0.1240 | 0.1268 | 0.0663 | 0.0835 | 0.0822 |
| S86 | 0.1889 | 0.1887 | 0.1887 | 0.1896 | 0.1861 | 0.2254 | 0.2319 | 0.2267 | 0.1815 | 0.1891 | 0.1756 | 0.1822 | 0.1832 | 0.1783 | 0.1770 | 0.1798 | 0.1472 | 0.1643 | 0.1630 |
| S34 | 0.1745 | 0.1743 | 0.1743 | 0.1751 | 0.1716 | 0.2109 | 0.2174 | 0.2123 | 0.2244 | 0.2320 | 0.2184 | 0.2251 | 0.2261 | 0.2211 | 0.2199 | 0.2227 | 0.1900 | 0.2072 | 0.2059 |
| S84 | 0.1770 | 0.1768 | 0.1768 | 0.1777 | 0.1741 | 0.2134 | 0.2200 | 0.2148 | 0.2269 | 0.2345 | 0.2210 | 0.2276 | 0.2286 | 0.2237 | 0.2224 | 0.2252 | 0.1926 | 0.2098 | 0.2084 |
| S55 | 0.1780 | 0.1778 | 0.1778 | 0.1787 | 0.1751 | 0.2144 | 0.2209 | 0.2158 | 0.2279 | 0.2355 | 0.2219 | 0.2286 | 0.2296 | 0.2246 | 0.2234 | 0.2262 | 0.1935 | 0.2107 | 0.2094 |
| S52 | 0.1652 | 0.1650 | 0.1650 | 0.1658 | 0.1623 | 0.2016 | 0.2081 | 0.2029 | 0.2151 | 0.2227 | 0.2091 | 0.2158 | 0.2168 | 0.2118 | 0.2106 | 0.2134 | 0.1807 | 0.1979 | 0.1966 |
| S38 | 0.1240 | 0.1238 | 0.1238 | 0.1246 | 0.1211 | 0.1604 | 0.1669 | 0.1617 | 0.2201 | 0.2276 | 0.2141 | 0.2208 | 0.2218 | 0.2168 | 0.2156 | 0.2183 | 0.1857 | 0.2029 | 0.2016 |
| S101 | 0.2797 | 0.2795 | 0.2795 | 0.2804 | 0.2768 | 0.3161 | 0.3227 | 0.3175 | 0.3758 | 0.3834 | 0.3699 | 0.3765 | 0.3775 | 0.3726 | 0.3713 | 0.3741 | 0.3415 | 0.3586 | 0.3573 |
| S102 | 0.2813 | 0.2810 | 0.2811 | 0.2819 | 0.2784 | 0.3177 | 0.3242 | 0.3190 | 0.3774 | 0.3849 | 0.3714 | 0.3780 | 0.3790 | 0.3741 | 0.3729 | 0.3756 | 0.3430 | 0.3602 | 0.3589 |
| S85 | 0.2817 | 0.2815 | 0.2815 | 0.2824 | 0.2789 | 0.3182 | 0.3247 | 0.3195 | 0.3779 | 0.3854 | 0.3719 | 0.3785 | 0.3795 | 0.3746 | 0.3734 | 0.3761 | 0.3435 | 0.3607 | 0.3593 |
| S63 | 0.2928 | 0.2926 | 0.2926 | 0.2935 | 0.2900 | 0.3293 | 0.3358 | 0.3306 | 0.3889 | 0.3965 | 0.3830 | 0.3896 | 0.3906 | 0.3857 | 0.3845 | 0.3872 | 0.3546 | 0.3718 | 0.3704 |
| S72 | 0.2911 | 0.2909 | 0.2909 | 0.2918 | 0.2883 | 0.3276 | 0.3341 | 0.3289 | 0.3872 | 0.3948 | 0.3813 | 0.3879 | 0.3889 | 0.3840 | 0.3828 | 0.3855 | 0.3529 | 0.3701 | 0.3687 |
| S71 | 0.2854 | 0.2852 | 0.2852 | 0.2861 | 0.2826 | 0.3219 | 0.3284 | 0.3232 | 0.3815 | 0.3891 | 0.3756 | 0.3822 | 0.3832 | 0.3783 | 0.3771 | 0.3798 | 0.3472 | 0.3644 | 0.3630 |
| S2 | 0.2576 | 0.2574 | 0.2574 | 0.2582 | 0.2547 | 0.2940 | 0.3005 | 0.2953 | 0.3537 | 0.3612 | 0.3477 | 0.3544 | 0.3554 | 0.3504 | 0.3492 | 0.3519 | 0.3193 | 0.3365 | 0.3352 |
| S3 | 0.2479 | 0.2477 | 0.2477 | 0.2486 | 0.2450 | 0.2843 | 0.2909 | 0.2857 | 0.3440 | 0.3516 | 0.3381 | 0.3447 | 0.3457 | 0.3407 | 0.3395 | 0.3423 | 0.3096 | 0.3268 | 0.3255 |
| S26 | 0.2350 | 0.2348 | 0.2348 | 0.2357 | 0.2321 | 0.2714 | 0.2779 | 0.2728 | 0.3311 | 0.3387 | 0.3251 | 0.3318 | 0.3328 | 0.3278 | 0.3266 | 0.3294 | 0.2967 | 0.3139 | 0.3126 |
| S88 | 0.2012 | 0.2010 | 0.2010 | 0.2019 | 0.1984 | 0.2377 | 0.2442 | 0.2390 | 0.2973 | 0.3049 | 0.2914 | 0.2980 | 0.2990 | 0.2941 | 0.2929 | 0.2956 | 0.2630 | 0.2802 | 0.2788 |
| S104 | 0.2177 | 0.2175 | 0.2175 | 0.2183 | 0.2148 | 0.2541 | 0.2606 | 0.2554 | 0.3138 | 0.3213 | 0.3078 | 0.3145 | 0.3155 | 0.3105 | 0.3093 | 0.3120 | 0.2794 | 0.2966 | 0.2953 |
| S4 | 0.2367 | 0.2365 | 0.2365 | 0.2374 | 0.2338 | 0.2731 | 0.2796 | 0.2745 | 0.3328 | 0.3404 | 0.3268 | 0.3335 | 0.3345 | 0.3295 | 0.3283 | 0.3311 | 0.2984 | 0.3156 | 0.3143 |
| S6 | 0.2364 | 0.2362 | 0.2362 | 0.2371 | 0.2335 | 0.2728 | 0.2794 | 0.2742 | 0.3325 | 0.3401 | 0.3266 | 0.3332 | 0.3342 | 0.3293 | 0.3280 | 0.3308 | 0.2981 | 0.3153 | 0.3140 |
| S103 | 0.2968 | 0.2966 | 0.2966 | 0.2975 | 0.2939 | 0.3332 | 0.3397 | 0.3346 | 0.3929 | 0.4005 | 0.3869 | 0.3936 | 0.3946 | 0.3896 | 0.3884 | 0.3912 | 0.3585 | 0.3757 | 0.3744 |
| S7 | 0.3360 | 0.3358 | 0.3358 | 0.3367 | 0.3332 | 0.3725 | 0.3790 | 0.3738 | 0.4321 | 0.4397 | 0.4262 | 0.4328 | 0.4338 | 0.4289 | 0.4277 | 0.4304 | 0.3978 | 0.4150 | 0.4136 |
| S8 | 0.3378 | 0.3376 | 0.3376 | 0.3385 | 0.3349 | 0.3742 | 0.3807 | 0.3756 | 0.4339 | 0.4415 | 0.4279 | 0.4346 | 0.4356 | 0.4306 | 0.4294 | 0.4322 | 0.3995 | 0.4167 | 0.4154 |
| S76 | 0.3409 | 0.3407 | 0.3407 | 0.3416 | 0.3381 | 0.3774 | 0.3839 | 0.3787 | 0.4370 | 0.4446 | 0.4311 | 0.4377 | 0.4387 | 0.4338 | 0.4325 | 0.4353 | 0.4027 | 0.4199 | 0.4185 |
| S78 | 0.3365 | 0.3363 | 0.3363 | 0.3372 | 0.3336 | 0.3729 | 0.3795 | 0.3743 | 0.4326 | 0.4402 | 0.4267 | 0.4333 | 0.4343 | 0.4294 | 0.4281 | 0.4309 | 0.3982 | 0.4154 | 0.4141 |
| S77 | 0.3183 | 0.3181 | 0.3181 | 0.3189 | 0.3154 | 0.3547 | 0.3612 | 0.3560 | 0.4144 | 0.4220 | 0.4084 | 0.4151 | 0.4161 | 0.4111 | 0.4099 | 0.4126 | 0.3800 | 0.3972 | 0.3959 |
| S105 | 0.3009 | 0.3006 | 0.3007 | 0.3015 | 0.2980 | 0.3373 | 0.3438 | 0.3386 | 0.3970 | 0.4045 | 0.3910 | 0.3977 | 0.3986 | 0.3937 | 0.3925 | 0.3952 | 0.3626 | 0.3798 | 0.3785 |
| S15 | 0.3067 | 0.3065 | 0.3065 | 0.3074 | 0.3038 | 0.3431 | 0.3497 | 0.3445 | 0.4028 | 0.4104 | 0.3969 | 0.4035 | 0.4045 | 0.3996 | 0.3983 | 0.4011 | 0.3684 | 0.3856 | 0.3843 |
| S19 | 0.3157 | 0.3155 | 0.3155 | 0.3164 | 0.3128 | 0.3521 | 0.3586 | 0.3535 | 0.4118 | 0.4194 | 0.4058 | 0.4125 | 0.4135 | 0.4085 | 0.4073 | 0.4101 | 0.3774 | 0.3946 | 0.3933 |
| S20 | 0.3163 | 0.3161 | 0.3161 | 0.3170 | 0.3134 | 0.3527 | 0.3593 | 0.3541 | 0.4124 | 0.4200 | 0.4065 | 0.4131 | 0.4141 | 0.4092 | 0.4079 | 0.4107 | 0.3780 | 0.3952 | 0.3939 |
| S29 | 0.3281 | 0.3279 | 0.3279 | 0.3288 | 0.3252 | 0.3645 | 0.3710 | 0.3659 | 0.4242 | 0.4318 | 0.4182 | 0.4249 | 0.4259 | 0.4209 | 0.4197 | 0.4225 | 0.3898 | 0.4070 | 0.4057 |
| S31 | 0.3187 | 0.3184 | 0.3185 | 0.3193 | 0.3158 | 0.3551 | 0.3616 | 0.3564 | 0.4148 | 0.4223 | 0.4088 | 0.4155 | 0.4164 | 0.4115 | 0.4103 | 0.4130 | 0.3804 | 0.3976 | 0.3963 |
| S33 | 0.3129 | 0.3127 | 0.3127 | 0.3136 | 0.3100 | 0.3494 | 0.3559 | 0.3507 | 0.4090 | 0.4166 | 0.4031 | 0.4097 | 0.4107 | 0.4058 | 0.4045 | 0.4073 | 0.3747 | 0.3919 | 0.3905 |
| S35 | 0.3009 | 0.3007 | 0.3007 | 0.3016 | 0.2980 | 0.3373 | 0.3439 | 0.3387 | 0.3970 | 0.4046 | 0.3911 | 0.3977 | 0.3987 | 0.3938 | 0.3925 | 0.3953 | 0.3627 | 0.3798 | 0.3785 |
| S16 | 0.2596 | 0.2594 | 0.2594 | 0.2602 | 0.2567 | 0.2960 | 0.3025 | 0.2973 | 0.3557 | 0.3633 | 0.3497 | 0.3564 | 0.3574 | 0.3524 | 0.3512 | 0.3539 | 0.3213 | 0.3385 | 0.3372 |
| S17 | 0.2603 | 0.2601 | 0.2601 | 0.2610 | 0.2575 | 0.2968 | 0.3033 | 0.2981 | 0.3564 | 0.3640 | 0.3505 | 0.3571 | 0.3581 | 0.3532 | 0.3520 | 0.3547 | 0.3221 | 0.3393 | 0.3379 |
| S18 | 0.2548 | 0.2546 | 0.2546 | 0.2555 | 0.2520 | 0.2913 | 0.2978 | 0.2926 | 0.3509 | 0.3585 | 0.3450 | 0.3516 | 0.3526 | 0.3477 | 0.3464 | 0.3492 | 0.3166 | 0.3338 | 0.3324 |
| S30 | 0.2006 | 0.2004 | 0.2004 | 0.2013 | 0.1977 | 0.2370 | 0.2436 | 0.2384 | 0.2967 | 0.3043 | 0.2908 | 0.2974 | 0.2984 | 0.2935 | 0.2922 | 0.2950 | 0.2623 | 0.2795 | 0.2782 |
| S37 | 0.2741 | 0.2739 | 0.2739 | 0.2748 | 0.2713 | 0.3106 | 0.3171 | 0.3119 | 0.3702 | 0.3778 | 0.3643 | 0.3709 | 0.3719 | 0.3670 | 0.3658 | 0.3685 | 0.3359 | 0.3531 | 0.3517 |
| S49 | 0.2723 | 0.2720 | 0.2721 | 0.2729 | 0.2694 | 0.3087 | 0.3152 | 0.3100 | 0.3684 | 0.3759 | 0.3624 | 0.3691 | 0.3700 | 0.3651 | 0.3639 | 0.3666 | 0.3340 | 0.3512 | 0.3499 |
| S50 | 0.2833 | 0.2831 | 0.2831 | 0.2840 | 0.2804 | 0.3197 | 0.3263 | 0.3211 | 0.3794 | 0.3870 | 0.3735 | 0.3801 | 0.3811 | 0.3762 | 0.3749 | 0.3777 | 0.3451 | 0.3622 | 0.3609 |
| S39 | 0.2822 | 0.2820 | 0.2820 | 0.2829 | 0.2793 | 0.3186 | 0.3251 | 0.3200 | 0.3783 | 0.3859 | 0.3723 | 0.3790 | 0.3800 | 0.3750 | 0.3738 | 0.3766 | 0.3439 | 0.3611 | 0.3598 |
| S96 | 0.2968 | 0.2966 | 0.2966 | 0.2975 | 0.2939 | 0.3332 | 0.3398 | 0.3346 | 0.3929 | 0.4005 | 0.3870 | 0.3936 | 0.3946 | 0.3897 | 0.3884 | 0.3912 | 0.3585 | 0.3757 | 0.3744 |
| S98 | 0.3020 | 0.3018 | 0.3018 | 0.3027 | 0.2992 | 0.3385 | 0.3450 | 0.3398 | 0.3981 | 0.4057 | 0.3922 | 0.3988 | 0.3998 | 0.3949 | 0.3937 | 0.3964 | 0.3638 | 0.3810 | 0.3796 |
| S99 | 0.3048 | 0.3046 | 0.3046 | 0.3055 | 0.3019 | 0.3412 | 0.3478 | 0.3426 | 0.4009 | 0.4085 | 0.3950 | 0.4016 | 0.4026 | 0.3976 | 0.3964 | 0.3992 | 0.3665 | 0.3837 | 0.3824 |
| S5 | 0.2703 | 0.2701 | 0.2701 | 0.2709 | 0.2674 | 0.3067 | 0.3132 | 0.3081 | 0.3664 | 0.3740 | 0.3604 | 0.3671 | 0.3681 | 0.3631 | 0.3619 | 0.3647 | 0.3320 | 0.3492 | 0.3479 |
| S14 | 0.2201 | 0.2199 | 0.2199 | 0.2208 | 0.2172 | 0.2565 | 0.2630 | 0.2579 | 0.3162 | 0.3238 | 0.3102 | 0.3169 | 0.3179 | 0.3129 | 0.3117 | 0.3145 | 0.2818 | 0.2990 | 0.2977 |
| S61 | 0.2308 | 0.2306 | 0.2306 | 0.2315 | 0.2279 | 0.2672 | 0.2737 | 0.2686 | 0.3269 | 0.3345 | 0.3209 | 0.3276 | 0.3286 | 0.3236 | 0.3224 | 0.3252 | 0.2925 | 0.3097 | 0.3084 |
| S62 | 0.2332 | 0.2330 | 0.2330 | 0.2339 | 0.2304 | 0.2697 | 0.2762 | 0.2710 | 0.3293 | 0.3369 | 0.3234 | 0.3300 | 0.3310 | 0.3261 | 0.3248 | 0.3276 | 0.2950 | 0.3122 | 0.3108 |
| S11 | 0.3309 | 0.3307 | 0.3307 | 0.3316 | 0.3280 | 0.3673 | 0.3738 | 0.3687 | 0.4270 | 0.4346 | 0.4210 | 0.4277 | 0.4287 | 0.4237 | 0.4225 | 0.4253 | 0.3926 | 0.4098 | 0.4085 |
| S13 | 0.3365 | 0.3363 | 0.3363 | 0.3372 | 0.3336 | 0.3729 | 0.3794 | 0.3743 | 0.4326 | 0.4402 | 0.4266 | 0.4333 | 0.4343 | 0.4293 | 0.4281 | 0.4309 | 0.3982 | 0.4154 | 0.4141 |
| S65 | 0.3310 | 0.3308 | 0.3308 | 0.3317 | 0.3281 | 0.3674 | 0.3739 | 0.3688 | 0.4271 | 0.4347 | 0.4211 | 0.4278 | 0.4288 | 0.4238 | 0.4226 | 0.4254 | 0.3927 | 0.4099 | 0.4086 |
| S42 | 0.3227 | 0.3224 | 0.3225 | 0.3233 | 0.3198 | 0.3591 | 0.3656 | 0.3604 | 0.4188 | 0.4263 | 0.4128 | 0.4194 | 0.4204 | 0.4155 | 0.4143 | 0.4170 | 0.3844 | 0.4016 | 0.4003 |
| S57 | 0.3086 | 0.3084 | 0.3084 | 0.3093 | 0.3057 | 0.3450 | 0.3516 | 0.3464 | 0.4047 | 0.4123 | 0.3988 | 0.4054 | 0.4064 | 0.4015 | 0.4002 | 0.4030 | 0.3704 | 0.3876 | 0.3862 |
| S36 | 0.2996 | 0.2994 | 0.2994 | 0.3003 | 0.2967 | 0.3360 | 0.3425 | 0.3374 | 0.3957 | 0.4033 | 0.3897 | 0.3964 | 0.3974 | 0.3924 | 0.3912 | 0.3940 | 0.3613 | 0.3785 | 0.3772 |
| S89 | 0.3012 | 0.3010 | 0.3010 | 0.3018 | 0.2983 | 0.3376 | 0.3441 | 0.3389 | 0.3973 | 0.4049 | 0.3913 | 0.3980 | 0.3990 | 0.3940 | 0.3928 | 0.3955 | 0.3629 | 0.3801 | 0.3788 |
| S58 | 0.2706 | 0.2704 | 0.2704 | 0.2713 | 0.2677 | 0.3070 | 0.3136 | 0.3084 | 0.3667 | 0.3743 | 0.3608 | 0.3674 | 0.3684 | 0.3635 | 0.3622 | 0.3650 | 0.3323 | 0.3495 | 0.3482 |
| S59 | 0.2624 | 0.2622 | 0.2622 | 0.2630 | 0.2595 | 0.2988 | 0.3053 | 0.3001 | 0.3585 | 0.3660 | 0.3525 | 0.3592 | 0.3602 | 0.3552 | 0.3540 | 0.3567 | 0.3241 | 0.3413 | 0.3400 |
| S74 | 0.2737 | 0.2735 | 0.2735 | 0.2743 | 0.2708 | 0.3101 | 0.3166 | 0.3115 | 0.3698 | 0.3774 | 0.3638 | 0.3705 | 0.3715 | 0.3665 | 0.3653 | 0.3681 | 0.3354 | 0.3526 | 0.3513 |
| S75 | 0.2802 | 0.2800 | 0.2800 | 0.2809 | 0.2773 | 0.3166 | 0.3232 | 0.3180 | 0.3763 | 0.3839 | 0.3704 | 0.3770 | 0.3780 | 0.3731 | 0.3718 | 0.3746 | 0.3420 | 0.3592 | 0.3578 |
| S27 | 0.2161 | 0.2159 | 0.2159 | 0.2168 | 0.2132 | 0.2525 | 0.2590 | 0.2539 | 0.3122 | 0.3198 | 0.3062 | 0.3129 | 0.3139 | 0.3089 | 0.3077 | 0.3105 | 0.2778 | 0.2950 | 0.2937 |
| S28 | 0.2375 | 0.2373 | 0.2373 | 0.2382 | 0.2346 | 0.2739 | 0.2805 | 0.2753 | 0.3336 | 0.3412 | 0.3277 | 0.3343 | 0.3353 | 0.3304 | 0.3291 | 0.3319 | 0.2992 | 0.3164 | 0.3151 |
| S90 | 0.2782 | 0.2780 | 0.2780 | 0.2788 | 0.2753 | 0.3146 | 0.3211 | 0.3160 | 0.3743 | 0.3819 | 0.3683 | 0.3750 | 0.3760 | 0.3710 | 0.3698 | 0.3726 | 0.3399 | 0.3571 | 0.3558 |
| S91 | 0.2932 | 0.2930 | 0.2930 | 0.2938 | 0.2903 | 0.3296 | 0.3361 | 0.3309 | 0.3893 | 0.3969 | 0.3833 | 0.3900 | 0.3910 | 0.3860 | 0.3848 | 0.3875 | 0.3549 | 0.3721 | 0.3708 |
| S95 | 0.2973 | 0.2971 | 0.2971 | 0.2980 | 0.2944 | 0.3337 | 0.3402 | 0.3351 | 0.3934 | 0.4010 | 0.3874 | 0.3941 | 0.3951 | 0.3901 | 0.3889 | 0.3917 | 0.3590 | 0.3762 | 0.3749 |
| S93 | 0.2886 | 0.2884 | 0.2884 | 0.2893 | 0.2858 | 0.3251 | 0.3316 | 0.3264 | 0.3847 | 0.3923 | 0.3788 | 0.3854 | 0.3864 | 0.3815 | 0.3803 | 0.3830 | 0.3504 | 0.3676 | 0.3662 |
| S92 | 0.2810 | 0.2808 | 0.2808 | 0.2817 | 0.2782 | 0.3175 | 0.3240 | 0.3188 | 0.3771 | 0.3847 | 0.3712 | 0.3778 | 0.3788 | 0.3739 | 0.3726 | 0.3754 | 0.3428 | 0.3600 | 0.3586 |

Table S5 (Continued)

| Sample ID | S47 | S56 | S53 | S60 | S54 | S80 | S9 | S86 | S34 | S84 | S55 | S52 | S38 | S101 | S102 | S85 | S63 | S72 | S71 |
| --- | --- | --- | --- | --- | --- | --- | --- | --- | --- | --- | --- | --- | --- | --- | --- | --- | --- | --- | --- |
| S1 | 0.1768 | 0.1765 | 0.1766 | 0.1764 | 0.1788 | 0.1780 | 0.1661 | 0.1889 | 0.1745 | 0.1770 | 0.1780 | 0.1652 | 0.1240 | 0.2797 | 0.2813 | 0.2817 | 0.2928 | 0.2911 | 0.2854 |
| S48 | 0.1766 | 0.1763 | 0.1764 | 0.1761 | 0.1786 | 0.1778 | 0.1659 | 0.1887 | 0.1743 | 0.1768 | 0.1778 | 0.1650 | 0.1238 | 0.2795 | 0.2810 | 0.2815 | 0.2926 | 0.2909 | 0.2852 |
| S21 | 0.1766 | 0.1763 | 0.1764 | 0.1761 | 0.1786 | 0.1778 | 0.1659 | 0.1887 | 0.1743 | 0.1768 | 0.1778 | 0.1650 | 0.1238 | 0.2795 | 0.2811 | 0.2815 | 0.2926 | 0.2909 | 0.2852 |
| S73 | 0.1775 | 0.1771 | 0.1773 | 0.1770 | 0.1794 | 0.1786 | 0.1668 | 0.1896 | 0.1751 | 0.1777 | 0.1787 | 0.1658 | 0.1246 | 0.2804 | 0.2819 | 0.2824 | 0.2935 | 0.2918 | 0.2861 |
| S97 | 0.1740 | 0.1736 | 0.1737 | 0.1735 | 0.1759 | 0.1751 | 0.1632 | 0.1861 | 0.1716 | 0.1741 | 0.1751 | 0.1623 | 0.1211 | 0.2768 | 0.2784 | 0.2789 | 0.2900 | 0.2883 | 0.2826 |
| S40 | 0.2133 | 0.2129 | 0.2130 | 0.2128 | 0.2152 | 0.2144 | 0.2025 | 0.2254 | 0.2109 | 0.2134 | 0.2144 | 0.2016 | 0.1604 | 0.3161 | 0.3177 | 0.3182 | 0.3293 | 0.3276 | 0.3219 |
| S41 | 0.2198 | 0.2194 | 0.2195 | 0.2193 | 0.2217 | 0.2209 | 0.2091 | 0.2319 | 0.2174 | 0.2200 | 0.2209 | 0.2081 | 0.1669 | 0.3227 | 0.3242 | 0.3247 | 0.3358 | 0.3341 | 0.3284 |
| S87 | 0.2146 | 0.2142 | 0.2144 | 0.2141 | 0.2165 | 0.2158 | 0.2039 | 0.2267 | 0.2123 | 0.2148 | 0.2158 | 0.2029 | 0.1617 | 0.3175 | 0.3190 | 0.3195 | 0.3306 | 0.3289 | 0.3232 |
| S10 | 0.1392 | 0.1389 | 0.1390 | 0.1387 | 0.1412 | 0.1404 | 0.1285 | 0.1815 | 0.2244 | 0.2269 | 0.2279 | 0.2151 | 0.2201 | 0.3758 | 0.3774 | 0.3779 | 0.3889 | 0.3872 | 0.3815 |
| S51 | 0.1468 | 0.1464 | 0.1466 | 0.1463 | 0.1487 | 0.1480 | 0.1361 | 0.1891 | 0.2320 | 0.2345 | 0.2355 | 0.2227 | 0.2276 | 0.3834 | 0.3849 | 0.3854 | 0.3965 | 0.3948 | 0.3891 |
| S79 | 0.1333 | 0.1329 | 0.1330 | 0.1328 | 0.1352 | 0.1344 | 0.1225 | 0.1756 | 0.2184 | 0.2210 | 0.2219 | 0.2091 | 0.2141 | 0.3699 | 0.3714 | 0.3719 | 0.3830 | 0.3813 | 0.3756 |
| S23 | 0.1399 | 0.1396 | 0.1397 | 0.1394 | 0.1418 | 0.1411 | 0.1292 | 0.1822 | 0.2251 | 0.2276 | 0.2286 | 0.2158 | 0.2208 | 0.3765 | 0.3780 | 0.3785 | 0.3896 | 0.3879 | 0.3822 |
| S45 | 0.1409 | 0.1406 | 0.1407 | 0.1404 | 0.1428 | 0.1421 | 0.1302 | 0.1832 | 0.2261 | 0.2286 | 0.2296 | 0.2168 | 0.2218 | 0.3775 | 0.3790 | 0.3795 | 0.3906 | 0.3889 | 0.3832 |
| S46 | 0.1360 | 0.1356 | 0.1357 | 0.1355 | 0.1379 | 0.1371 | 0.1252 | 0.1783 | 0.2211 | 0.2237 | 0.2246 | 0.2118 | 0.2168 | 0.3726 | 0.3741 | 0.3746 | 0.3857 | 0.3840 | 0.3783 |
| S24 | 0.1348 | 0.1344 | 0.1345 | 0.1343 | 0.1367 | 0.1359 | 0.1240 | 0.1770 | 0.2199 | 0.2224 | 0.2234 | 0.2106 | 0.2156 | 0.3713 | 0.3729 | 0.3734 | 0.3845 | 0.3828 | 0.3771 |
| S32 | 0.1375 | 0.1371 | 0.1373 | 0.1370 | 0.1394 | 0.1386 | 0.1268 | 0.1798 | 0.2227 | 0.2252 | 0.2262 | 0.2134 | 0.2183 | 0.3741 | 0.3756 | 0.3761 | 0.3872 | 0.3855 | 0.3798 |
| S12 | 0.0612 | 0.0609 | 0.0768 | 0.0766 | 0.0790 | 0.0782 | 0.0663 | 0.1472 | 0.1900 | 0.1926 | 0.1935 | 0.1807 | 0.1857 | 0.3415 | 0.3430 | 0.3435 | 0.3546 | 0.3529 | 0.3472 |
| S22 | 0.0560 | 0.0556 | 0.0940 | 0.0938 | 0.0962 | 0.0954 | 0.0835 | 0.1643 | 0.2072 | 0.2098 | 0.2107 | 0.1979 | 0.2029 | 0.3586 | 0.3602 | 0.3607 | 0.3718 | 0.3701 | 0.3644 |
| S44 | 0.0547 | 0.0543 | 0.0927 | 0.0925 | 0.0949 | 0.0941 | 0.0822 | 0.1630 | 0.2059 | 0.2084 | 0.2094 | 0.1966 | 0.2016 | 0.3573 | 0.3589 | 0.3593 | 0.3704 | 0.3687 | 0.3630 |
| S47 | 0.0000 | 0.0280 | 0.0837 | 0.0835 | 0.0859 | 0.0851 | 0.0732 | 0.1540 | 0.1969 | 0.1994 | 0.2004 | 0.1876 | 0.1926 | 0.3483 | 0.3499 | 0.3504 | 0.3615 | 0.3598 | 0.3541 |
| S56 | 0.0280 | 0.0000 | 0.0833 | 0.0831 | 0.0855 | 0.0847 | 0.0729 | 0.1537 | 0.1965 | 0.1991 | 0.2000 | 0.1872 | 0.1922 | 0.3480 | 0.3495 | 0.3500 | 0.3611 | 0.3594 | 0.3537 |
| S53 | 0.0837 | 0.0833 | 0.0000 | 0.0288 | 0.0437 | 0.0429 | 0.0458 | 0.1538 | 0.1966 | 0.1992 | 0.2002 | 0.1873 | 0.1923 | 0.3481 | 0.3496 | 0.3501 | 0.3612 | 0.3595 | 0.3538 |
| S60 | 0.0835 | 0.0831 | 0.0288 | 0.0000 | 0.0434 | 0.0427 | 0.0455 | 0.1535 | 0.1964 | 0.1989 | 0.1999 | 0.1871 | 0.1921 | 0.3478 | 0.3494 | 0.3499 | 0.3610 | 0.3593 | 0.3536 |
| S54 | 0.0859 | 0.0855 | 0.0437 | 0.0434 | 0.0000 | 0.0153 | 0.0479 | 0.1560 | 0.1988 | 0.2014 | 0.2023 | 0.1895 | 0.1945 | 0.3503 | 0.3518 | 0.3523 | 0.3634 | 0.3617 | 0.3560 |
| S80 | 0.0851 | 0.0847 | 0.0429 | 0.0427 | 0.0153 | 0.0000 | 0.0471 | 0.1552 | 0.1980 | 0.2006 | 0.2015 | 0.1887 | 0.1937 | 0.3495 | 0.3510 | 0.3515 | 0.3626 | 0.3609 | 0.3552 |
| S9 | 0.0732 | 0.0729 | 0.0458 | 0.0455 | 0.0479 | 0.0471 | 0.0000 | 0.1433 | 0.1862 | 0.1887 | 0.1897 | 0.1769 | 0.1818 | 0.3376 | 0.3391 | 0.3396 | 0.3507 | 0.3490 | 0.3433 |
| S86 | 0.1540 | 0.1537 | 0.1538 | 0.1535 | 0.1560 | 0.1552 | 0.1433 | 0.0000 | 0.2090 | 0.2115 | 0.2125 | 0.1997 | 0.2047 | 0.3604 | 0.3620 | 0.3625 | 0.3736 | 0.3718 | 0.3662 |
| S34 | 0.1969 | 0.1965 | 0.1966 | 0.1964 | 0.1988 | 0.1980 | 0.1862 | 0.2090 | 0.0000 | 0.0481 | 0.0671 | 0.0893 | 0.1902 | 0.3460 | 0.3475 | 0.3480 | 0.3591 | 0.3574 | 0.3517 |
| S84 | 0.1994 | 0.1991 | 0.1992 | 0.1989 | 0.2014 | 0.2006 | 0.1887 | 0.2115 | 0.0481 | 0.0000 | 0.0697 | 0.0918 | 0.1928 | 0.3485 | 0.3500 | 0.3505 | 0.3616 | 0.3599 | 0.3542 |
| S55 | 0.2004 | 0.2000 | 0.2002 | 0.1999 | 0.2023 | 0.2015 | 0.1897 | 0.2125 | 0.0671 | 0.0697 | 0.0000 | 0.0928 | 0.1937 | 0.3495 | 0.3510 | 0.3515 | 0.3626 | 0.3609 | 0.3552 |
| S52 | 0.1876 | 0.1872 | 0.1873 | 0.1871 | 0.1895 | 0.1887 | 0.1769 | 0.1997 | 0.0893 | 0.0918 | 0.0928 | 0.0000 | 0.1809 | 0.3367 | 0.3382 | 0.3387 | 0.3498 | 0.3481 | 0.3424 |
| S38 | 0.1926 | 0.1922 | 0.1923 | 0.1921 | 0.1945 | 0.1937 | 0.1818 | 0.2047 | 0.1902 | 0.1928 | 0.1937 | 0.1809 | 0.0000 | 0.2206 | 0.2221 | 0.2226 | 0.2337 | 0.2320 | 0.2263 |
| S101 | 0.3483 | 0.3480 | 0.3481 | 0.3478 | 0.3503 | 0.3495 | 0.3376 | 0.3604 | 0.3460 | 0.3485 | 0.3495 | 0.3367 | 0.2206 | 0.0000 | 0.0159 | 0.0400 | 0.0808 | 0.0791 | 0.0734 |
| S102 | 0.3499 | 0.3495 | 0.3496 | 0.3494 | 0.3518 | 0.3510 | 0.3391 | 0.3620 | 0.3475 | 0.3500 | 0.3510 | 0.3382 | 0.2221 | 0.0159 | 0.0000 | 0.0415 | 0.0823 | 0.0806 | 0.0749 |
| S85 | 0.3504 | 0.3500 | 0.3501 | 0.3499 | 0.3523 | 0.3515 | 0.3396 | 0.3625 | 0.3480 | 0.3505 | 0.3515 | 0.3387 | 0.2226 | 0.0400 | 0.0415 | 0.0000 | 0.0828 | 0.0811 | 0.0754 |
| S63 | 0.3615 | 0.3611 | 0.3612 | 0.3610 | 0.3634 | 0.3626 | 0.3507 | 0.3736 | 0.3591 | 0.3616 | 0.3626 | 0.3498 | 0.2337 | 0.0808 | 0.0823 | 0.0828 | 0.0000 | 0.0350 | 0.0474 |
| S72 | 0.3598 | 0.3594 | 0.3595 | 0.3593 | 0.3617 | 0.3609 | 0.3490 | 0.3718 | 0.3574 | 0.3599 | 0.3609 | 0.3481 | 0.2320 | 0.0791 | 0.0806 | 0.0811 | 0.0350 | 0.0000 | 0.0457 |
| S71 | 0.3541 | 0.3537 | 0.3538 | 0.3536 | 0.3560 | 0.3552 | 0.3433 | 0.3662 | 0.3517 | 0.3542 | 0.3552 | 0.3424 | 0.2263 | 0.0734 | 0.0749 | 0.0754 | 0.0474 | 0.0457 | 0.0000 |
| S2 | 0.3262 | 0.3258 | 0.3259 | 0.3257 | 0.3281 | 0.3273 | 0.3154 | 0.3383 | 0.3238 | 0.3264 | 0.3273 | 0.3145 | 0.1984 | 0.1350 | 0.1365 | 0.1370 | 0.1481 | 0.1464 | 0.1407 |
| S3 | 0.3165 | 0.3161 | 0.3163 | 0.3160 | 0.3184 | 0.3176 | 0.3058 | 0.3286 | 0.3141 | 0.3167 | 0.3177 | 0.3048 | 0.1888 | 0.1253 | 0.1269 | 0.1274 | 0.1385 | 0.1368 | 0.1311 |
| S26 | 0.3036 | 0.3032 | 0.3033 | 0.3031 | 0.3055 | 0.3047 | 0.2929 | 0.3157 | 0.3012 | 0.3038 | 0.3047 | 0.2919 | 0.1758 | 0.1124 | 0.1140 | 0.1145 | 0.1255 | 0.1238 | 0.1181 |
| S88 | 0.2699 | 0.2695 | 0.2696 | 0.2694 | 0.2718 | 0.2710 | 0.2591 | 0.2820 | 0.2675 | 0.2700 | 0.2710 | 0.2582 | 0.1421 | 0.1006 | 0.1021 | 0.1026 | 0.1137 | 0.1120 | 0.1063 |
| S104 | 0.2863 | 0.2859 | 0.2860 | 0.2858 | 0.2882 | 0.2874 | 0.2755 | 0.2984 | 0.2839 | 0.2865 | 0.2874 | 0.2746 | 0.1585 | 0.1531 | 0.1546 | 0.1551 | 0.1662 | 0.1645 | 0.1588 |
| S4 | 0.3053 | 0.3049 | 0.3050 | 0.3048 | 0.3072 | 0.3064 | 0.2946 | 0.3174 | 0.3029 | 0.3055 | 0.3064 | 0.2936 | 0.1775 | 0.1721 | 0.1737 | 0.1742 | 0.1852 | 0.1835 | 0.1778 |
| S6 | 0.3050 | 0.3046 | 0.3048 | 0.3045 | 0.3069 | 0.3061 | 0.2943 | 0.3171 | 0.3026 | 0.3052 | 0.3062 | 0.2933 | 0.1773 | 0.1719 | 0.1734 | 0.1739 | 0.1850 | 0.1833 | 0.1776 |
| S103 | 0.3654 | 0.3650 | 0.3652 | 0.3649 | 0.3673 | 0.3665 | 0.3547 | 0.3775 | 0.3630 | 0.3656 | 0.3666 | 0.3537 | 0.2376 | 0.3204 | 0.3219 | 0.3224 | 0.3335 | 0.3318 | 0.3261 |
| S7 | 0.4047 | 0.4043 | 0.4044 | 0.4042 | 0.4066 | 0.4058 | 0.3939 | 0.4168 | 0.4023 | 0.4048 | 0.4058 | 0.3930 | 0.2769 | 0.3596 | 0.3612 | 0.3617 | 0.3728 | 0.3710 | 0.3654 |
| S8 | 0.4064 | 0.4060 | 0.4061 | 0.4059 | 0.4083 | 0.4075 | 0.3957 | 0.4185 | 0.4040 | 0.4066 | 0.4075 | 0.3947 | 0.2786 | 0.3614 | 0.3629 | 0.3634 | 0.3745 | 0.3728 | 0.3671 |
| S76 | 0.4095 | 0.4092 | 0.4093 | 0.4090 | 0.4115 | 0.4107 | 0.3988 | 0.4216 | 0.4072 | 0.4097 | 0.4107 | 0.3979 | 0.2818 | 0.3645 | 0.3661 | 0.3665 | 0.3776 | 0.3759 | 0.3702 |
| S78 | 0.4051 | 0.4048 | 0.4049 | 0.4046 | 0.4071 | 0.4063 | 0.3944 | 0.4172 | 0.4028 | 0.4053 | 0.4063 | 0.3935 | 0.2774 | 0.3601 | 0.3616 | 0.3621 | 0.3732 | 0.3715 | 0.3658 |
| S77 | 0.3869 | 0.3865 | 0.3866 | 0.3864 | 0.3888 | 0.3880 | 0.3761 | 0.3990 | 0.3845 | 0.3871 | 0.3880 | 0.3752 | 0.2591 | 0.3419 | 0.3434 | 0.3439 | 0.3550 | 0.3533 | 0.3476 |
| S105 | 0.3695 | 0.3691 | 0.3692 | 0.3690 | 0.3714 | 0.3706 | 0.3587 | 0.3816 | 0.3671 | 0.3696 | 0.3706 | 0.3578 | 0.2417 | 0.3244 | 0.3260 | 0.3265 | 0.3376 | 0.3359 | 0.3302 |
| S15 | 0.3753 | 0.3750 | 0.3751 | 0.3748 | 0.3772 | 0.3765 | 0.3646 | 0.3874 | 0.3730 | 0.3755 | 0.3765 | 0.3637 | 0.2476 | 0.3303 | 0.3318 | 0.3323 | 0.3434 | 0.3417 | 0.3360 |
| S19 | 0.3843 | 0.3839 | 0.3840 | 0.3838 | 0.3862 | 0.3854 | 0.3736 | 0.3964 | 0.3819 | 0.3845 | 0.3854 | 0.3726 | 0.2565 | 0.3393 | 0.3408 | 0.3413 | 0.3524 | 0.3507 | 0.3450 |
| S20 | 0.3849 | 0.3846 | 0.3847 | 0.3844 | 0.3869 | 0.3861 | 0.3742 | 0.3970 | 0.3826 | 0.3851 | 0.3861 | 0.3733 | 0.2572 | 0.3399 | 0.3414 | 0.3419 | 0.3530 | 0.3513 | 0.3456 |
| S29 | 0.3967 | 0.3963 | 0.3965 | 0.3962 | 0.3986 | 0.3978 | 0.3860 | 0.4088 | 0.3943 | 0.3969 | 0.3979 | 0.3850 | 0.2689 | 0.3517 | 0.3532 | 0.3537 | 0.3648 | 0.3631 | 0.3574 |
| S31 | 0.3873 | 0.3869 | 0.3870 | 0.3868 | 0.3892 | 0.3884 | 0.3765 | 0.3994 | 0.3849 | 0.3874 | 0.3884 | 0.3756 | 0.2595 | 0.3422 | 0.3438 | 0.3443 | 0.3554 | 0.3537 | 0.3480 |
| S33 | 0.3815 | 0.3812 | 0.3813 | 0.3810 | 0.3835 | 0.3827 | 0.3708 | 0.3936 | 0.3792 | 0.3817 | 0.3827 | 0.3699 | 0.2538 | 0.3365 | 0.3381 | 0.3385 | 0.3496 | 0.3479 | 0.3422 |
| S35 | 0.3695 | 0.3692 | 0.3693 | 0.3690 | 0.3715 | 0.3707 | 0.3588 | 0.3816 | 0.3672 | 0.3697 | 0.3707 | 0.3579 | 0.2418 | 0.3245 | 0.3260 | 0.3265 | 0.3376 | 0.3359 | 0.3302 |
| S16 | 0.3282 | 0.3278 | 0.3279 | 0.3277 | 0.3301 | 0.3293 | 0.3174 | 0.3403 | 0.3258 | 0.3284 | 0.3293 | 0.3165 | 0.2004 | 0.2832 | 0.2847 | 0.2852 | 0.2963 | 0.2946 | 0.2889 |
| S17 | 0.3290 | 0.3286 | 0.3287 | 0.3285 | 0.3309 | 0.3301 | 0.3182 | 0.3411 | 0.3266 | 0.3291 | 0.3301 | 0.3173 | 0.2012 | 0.2839 | 0.2855 | 0.2860 | 0.2970 | 0.2953 | 0.2896 |
| S18 | 0.3234 | 0.3231 | 0.3232 | 0.3229 | 0.3254 | 0.3246 | 0.3127 | 0.3355 | 0.3211 | 0.3236 | 0.3246 | 0.3118 | 0.1957 | 0.2784 | 0.2800 | 0.2804 | 0.2915 | 0.2898 | 0.2841 |
| S30 | 0.2692 | 0.2689 | 0.2690 | 0.2687 | 0.2711 | 0.2704 | 0.2585 | 0.2813 | 0.2669 | 0.2694 | 0.2704 | 0.2576 | 0.1415 | 0.2242 | 0.2257 | 0.2262 | 0.2373 | 0.2356 | 0.2299 |
| S37 | 0.3428 | 0.3424 | 0.3425 | 0.3423 | 0.3447 | 0.3439 | 0.3320 | 0.3549 | 0.3404 | 0.3429 | 0.3439 | 0.3311 | 0.2150 | 0.2977 | 0.2993 | 0.2998 | 0.3109 | 0.3091 | 0.3035 |
| S49 | 0.3409 | 0.3405 | 0.3406 | 0.3404 | 0.3428 | 0.3420 | 0.3301 | 0.3530 | 0.3385 | 0.3410 | 0.3420 | 0.3292 | 0.2131 | 0.2958 | 0.2974 | 0.2979 | 0.3090 | 0.3073 | 0.3016 |
| S50 | 0.3519 | 0.3516 | 0.3517 | 0.3514 | 0.3539 | 0.3531 | 0.3412 | 0.3640 | 0.3496 | 0.3521 | 0.3531 | 0.3403 | 0.2242 | 0.3069 | 0.3084 | 0.3089 | 0.3200 | 0.3183 | 0.3126 |
| S39 | 0.3508 | 0.3504 | 0.3505 | 0.3503 | 0.3527 | 0.3519 | 0.3401 | 0.3629 | 0.3484 | 0.3510 | 0.3519 | 0.3391 | 0.2230 | 0.3058 | 0.3073 | 0.3078 | 0.3189 | 0.3172 | 0.3115 |
| S96 | 0.3654 | 0.3651 | 0.3652 | 0.3649 | 0.3673 | 0.3666 | 0.3547 | 0.3775 | 0.3631 | 0.3656 | 0.3666 | 0.3538 | 0.2377 | 0.3204 | 0.3219 | 0.3224 | 0.3335 | 0.3318 | 0.3261 |
| S98 | 0.3706 | 0.3703 | 0.3704 | 0.3702 | 0.3726 | 0.3718 | 0.3599 | 0.3827 | 0.3683 | 0.3708 | 0.3718 | 0.3590 | 0.2429 | 0.3256 | 0.3272 | 0.3277 | 0.3387 | 0.3370 | 0.3313 |
| S99 | 0.3734 | 0.3730 | 0.3732 | 0.3729 | 0.3753 | 0.3745 | 0.3627 | 0.3855 | 0.3710 | 0.3736 | 0.3746 | 0.3617 | 0.2457 | 0.3284 | 0.3299 | 0.3304 | 0.3415 | 0.3398 | 0.3341 |
| S5 | 0.3389 | 0.3385 | 0.3386 | 0.3384 | 0.3408 | 0.3400 | 0.3281 | 0.3510 | 0.3365 | 0.3391 | 0.3400 | 0.3272 | 0.2111 | 0.2939 | 0.2954 | 0.2959 | 0.3070 | 0.3053 | 0.2996 |
| S14 | 0.2887 | 0.2883 | 0.2885 | 0.2882 | 0.2906 | 0.2898 | 0.2780 | 0.3008 | 0.2863 | 0.2889 | 0.2898 | 0.2770 | 0.1609 | 0.2437 | 0.2452 | 0.2457 | 0.2568 | 0.2551 | 0.2494 |
| S61 | 0.2994 | 0.2990 | 0.2991 | 0.2989 | 0.3013 | 0.3005 | 0.2887 | 0.3115 | 0.2970 | 0.2996 | 0.3005 | 0.2877 | 0.1716 | 0.2544 | 0.2559 | 0.2564 | 0.2675 | 0.2658 | 0.2601 |
| S62 | 0.3018 | 0.3015 | 0.3016 | 0.3013 | 0.3038 | 0.3030 | 0.2911 | 0.3139 | 0.2995 | 0.3020 | 0.3030 | 0.2902 | 0.1741 | 0.2568 | 0.2584 | 0.2588 | 0.2699 | 0.2682 | 0.2625 |
| S11 | 0.3995 | 0.3991 | 0.3993 | 0.3990 | 0.4014 | 0.4006 | 0.3888 | 0.4116 | 0.3971 | 0.3997 | 0.4006 | 0.3878 | 0.2717 | 0.3545 | 0.3560 | 0.3565 | 0.3676 | 0.3659 | 0.3602 |
| S13 | 0.4051 | 0.4047 | 0.4049 | 0.4046 | 0.4070 | 0.4062 | 0.3944 | 0.4172 | 0.4027 | 0.4053 | 0.4062 | 0.3934 | 0.2773 | 0.3601 | 0.3616 | 0.3621 | 0.3732 | 0.3715 | 0.3658 |
| S65 | 0.3996 | 0.3992 | 0.3993 | 0.3991 | 0.4015 | 0.4007 | 0.3889 | 0.4117 | 0.3972 | 0.3998 | 0.4007 | 0.3879 | 0.2718 | 0.3546 | 0.3561 | 0.3566 | 0.3677 | 0.3660 | 0.3603 |
| S42 | 0.3913 | 0.3909 | 0.3910 | 0.3908 | 0.3932 | 0.3924 | 0.3805 | 0.4034 | 0.3889 | 0.3914 | 0.3924 | 0.3796 | 0.2635 | 0.3462 | 0.3478 | 0.3483 | 0.3594 | 0.3577 | 0.3520 |
| S57 | 0.3772 | 0.3769 | 0.3770 | 0.3767 | 0.3792 | 0.3784 | 0.3665 | 0.3893 | 0.3749 | 0.3774 | 0.3784 | 0.3656 | 0.2495 | 0.3322 | 0.3338 | 0.3342 | 0.3453 | 0.3436 | 0.3379 |
| S36 | 0.3682 | 0.3678 | 0.3680 | 0.3677 | 0.3701 | 0.3693 | 0.3575 | 0.3803 | 0.3658 | 0.3684 | 0.3693 | 0.3565 | 0.2404 | 0.3232 | 0.3247 | 0.3252 | 0.3363 | 0.3346 | 0.3289 |
| S89 | 0.3698 | 0.3694 | 0.3695 | 0.3693 | 0.3717 | 0.3709 | 0.3590 | 0.3819 | 0.3674 | 0.3700 | 0.3709 | 0.3581 | 0.2420 | 0.3248 | 0.3263 | 0.3268 | 0.3379 | 0.3362 | 0.3305 |
| S58 | 0.3392 | 0.3389 | 0.3390 | 0.3387 | 0.3411 | 0.3404 | 0.3285 | 0.3513 | 0.3369 | 0.3394 | 0.3404 | 0.3276 | 0.2115 | 0.2942 | 0.2957 | 0.2962 | 0.3073 | 0.3056 | 0.2999 |
| S59 | 0.3310 | 0.3306 | 0.3307 | 0.3305 | 0.3329 | 0.3321 | 0.3202 | 0.3431 | 0.3286 | 0.3312 | 0.3321 | 0.3193 | 0.2032 | 0.2860 | 0.2875 | 0.2880 | 0.2991 | 0.2974 | 0.2917 |
| S74 | 0.3423 | 0.3419 | 0.3420 | 0.3418 | 0.3442 | 0.3434 | 0.3316 | 0.3544 | 0.3399 | 0.3425 | 0.3434 | 0.3306 | 0.2145 | 0.2973 | 0.2988 | 0.2993 | 0.3104 | 0.3087 | 0.3030 |
| S75 | 0.3488 | 0.3485 | 0.3486 | 0.3483 | 0.3508 | 0.3500 | 0.3381 | 0.3609 | 0.3465 | 0.3490 | 0.3500 | 0.3372 | 0.2211 | 0.3038 | 0.3053 | 0.3058 | 0.3169 | 0.3152 | 0.3095 |
| S27 | 0.2847 | 0.2843 | 0.2845 | 0.2842 | 0.2866 | 0.2858 | 0.2740 | 0.2968 | 0.2823 | 0.2849 | 0.2859 | 0.2730 | 0.1569 | 0.2397 | 0.2412 | 0.2417 | 0.2528 | 0.2511 | 0.2454 |
| S28 | 0.3061 | 0.3057 | 0.3059 | 0.3056 | 0.3080 | 0.3073 | 0.2954 | 0.3182 | 0.3038 | 0.3063 | 0.3073 | 0.2944 | 0.1784 | 0.2611 | 0.2626 | 0.2631 | 0.2742 | 0.2725 | 0.2668 |
| S90 | 0.3468 | 0.3464 | 0.3465 | 0.3463 | 0.3487 | 0.3479 | 0.3360 | 0.3589 | 0.3444 | 0.3470 | 0.3479 | 0.3351 | 0.2190 | 0.3018 | 0.3033 | 0.3038 | 0.3149 | 0.3132 | 0.3075 |
| S91 | 0.3618 | 0.3614 | 0.3615 | 0.3613 | 0.3637 | 0.3629 | 0.3510 | 0.3739 | 0.3594 | 0.3620 | 0.3629 | 0.3501 | 0.2340 | 0.3168 | 0.3183 | 0.3188 | 0.3299 | 0.3282 | 0.3225 |
| S95 | 0.3659 | 0.3655 | 0.3657 | 0.3654 | 0.3678 | 0.3670 | 0.3552 | 0.3780 | 0.3635 | 0.3661 | 0.3671 | 0.3542 | 0.2381 | 0.3209 | 0.3224 | 0.3229 | 0.3340 | 0.3323 | 0.3266 |
| S93 | 0.3573 | 0.3569 | 0.3570 | 0.3568 | 0.3592 | 0.3584 | 0.3465 | 0.3694 | 0.3549 | 0.3574 | 0.3584 | 0.3456 | 0.2295 | 0.3122 | 0.3138 | 0.3143 | 0.3254 | 0.3236 | 0.3180 |
| S92 | 0.3496 | 0.3493 | 0.3494 | 0.3491 | 0.3516 | 0.3508 | 0.3389 | 0.3617 | 0.3473 | 0.3498 | 0.3508 | 0.3380 | 0.2219 | 0.3046 | 0.3062 | 0.3066 | 0.3177 | 0.3160 | 0.3103 |

Table S5 (Continued)

| Sample ID | S2 | S3 | S26 | S88 | S104 | S4 | S6 | S103 | S7 | S8 | S76 | S78 | S77 | S105 | S15 | S19 | S20 | S29 | S31 |
| --- | --- | --- | --- | --- | --- | --- | --- | --- | --- | --- | --- | --- | --- | --- | --- | --- | --- | --- | --- |
| S1 | 0.2576 | 0.2479 | 0.2350 | 0.2012 | 0.2177 | 0.2367 | 0.2364 | 0.2968 | 0.3360 | 0.3378 | 0.3409 | 0.3365 | 0.3183 | 0.3009 | 0.3067 | 0.3157 | 0.3163 | 0.3281 | 0.3187 |
| S48 | 0.2574 | 0.2477 | 0.2348 | 0.2010 | 0.2175 | 0.2365 | 0.2362 | 0.2966 | 0.3358 | 0.3376 | 0.3407 | 0.3363 | 0.3181 | 0.3006 | 0.3065 | 0.3155 | 0.3161 | 0.3279 | 0.3184 |
| S21 | 0.2574 | 0.2477 | 0.2348 | 0.2010 | 0.2175 | 0.2365 | 0.2362 | 0.2966 | 0.3358 | 0.3376 | 0.3407 | 0.3363 | 0.3181 | 0.3007 | 0.3065 | 0.3155 | 0.3161 | 0.3279 | 0.3185 |
| S73 | 0.2582 | 0.2486 | 0.2357 | 0.2019 | 0.2183 | 0.2374 | 0.2371 | 0.2975 | 0.3367 | 0.3385 | 0.3416 | 0.3372 | 0.3189 | 0.3015 | 0.3074 | 0.3164 | 0.3170 | 0.3288 | 0.3193 |
| S97 | 0.2547 | 0.2450 | 0.2321 | 0.1984 | 0.2148 | 0.2338 | 0.2335 | 0.2939 | 0.3332 | 0.3349 | 0.3381 | 0.3336 | 0.3154 | 0.2980 | 0.3038 | 0.3128 | 0.3134 | 0.3252 | 0.3158 |
| S40 | 0.2940 | 0.2843 | 0.2714 | 0.2377 | 0.2541 | 0.2731 | 0.2728 | 0.3332 | 0.3725 | 0.3742 | 0.3774 | 0.3729 | 0.3547 | 0.3373 | 0.3431 | 0.3521 | 0.3527 | 0.3645 | 0.3551 |
| S41 | 0.3005 | 0.2909 | 0.2779 | 0.2442 | 0.2606 | 0.2796 | 0.2794 | 0.3397 | 0.3790 | 0.3807 | 0.3839 | 0.3795 | 0.3612 | 0.3438 | 0.3497 | 0.3586 | 0.3593 | 0.3710 | 0.3616 |
| S87 | 0.2953 | 0.2857 | 0.2728 | 0.2390 | 0.2554 | 0.2745 | 0.2742 | 0.3346 | 0.3738 | 0.3756 | 0.3787 | 0.3743 | 0.3560 | 0.3386 | 0.3445 | 0.3535 | 0.3541 | 0.3659 | 0.3564 |
| S10 | 0.3537 | 0.3440 | 0.3311 | 0.2973 | 0.3138 | 0.3328 | 0.3325 | 0.3929 | 0.4321 | 0.4339 | 0.4370 | 0.4326 | 0.4144 | 0.3970 | 0.4028 | 0.4118 | 0.4124 | 0.4242 | 0.4148 |
| S51 | 0.3612 | 0.3516 | 0.3387 | 0.3049 | 0.3213 | 0.3404 | 0.3401 | 0.4005 | 0.4397 | 0.4415 | 0.4446 | 0.4402 | 0.4220 | 0.4045 | 0.4104 | 0.4194 | 0.4200 | 0.4318 | 0.4223 |
| S79 | 0.3477 | 0.3381 | 0.3251 | 0.2914 | 0.3078 | 0.3268 | 0.3266 | 0.3869 | 0.4262 | 0.4279 | 0.4311 | 0.4267 | 0.4084 | 0.3910 | 0.3969 | 0.4058 | 0.4065 | 0.4182 | 0.4088 |
| S23 | 0.3544 | 0.3447 | 0.3318 | 0.2980 | 0.3145 | 0.3335 | 0.3332 | 0.3936 | 0.4328 | 0.4346 | 0.4377 | 0.4333 | 0.4151 | 0.3977 | 0.4035 | 0.4125 | 0.4131 | 0.4249 | 0.4155 |
| S45 | 0.3554 | 0.3457 | 0.3328 | 0.2990 | 0.3155 | 0.3345 | 0.3342 | 0.3946 | 0.4338 | 0.4356 | 0.4387 | 0.4343 | 0.4161 | 0.3986 | 0.4045 | 0.4135 | 0.4141 | 0.4259 | 0.4164 |
| S46 | 0.3504 | 0.3407 | 0.3278 | 0.2941 | 0.3105 | 0.3295 | 0.3293 | 0.3896 | 0.4289 | 0.4306 | 0.4338 | 0.4294 | 0.4111 | 0.3937 | 0.3996 | 0.4085 | 0.4092 | 0.4209 | 0.4115 |
| S24 | 0.3492 | 0.3395 | 0.3266 | 0.2929 | 0.3093 | 0.3283 | 0.3280 | 0.3884 | 0.4277 | 0.4294 | 0.4325 | 0.4281 | 0.4099 | 0.3925 | 0.3983 | 0.4073 | 0.4079 | 0.4197 | 0.4103 |
| S32 | 0.3519 | 0.3423 | 0.3294 | 0.2956 | 0.3120 | 0.3311 | 0.3308 | 0.3912 | 0.4304 | 0.4322 | 0.4353 | 0.4309 | 0.4126 | 0.3952 | 0.4011 | 0.4101 | 0.4107 | 0.4225 | 0.4130 |
| S12 | 0.3193 | 0.3096 | 0.2967 | 0.2630 | 0.2794 | 0.2984 | 0.2981 | 0.3585 | 0.3978 | 0.3995 | 0.4027 | 0.3982 | 0.3800 | 0.3626 | 0.3684 | 0.3774 | 0.3780 | 0.3898 | 0.3804 |
| S22 | 0.3365 | 0.3268 | 0.3139 | 0.2802 | 0.2966 | 0.3156 | 0.3153 | 0.3757 | 0.4150 | 0.4167 | 0.4199 | 0.4154 | 0.3972 | 0.3798 | 0.3856 | 0.3946 | 0.3952 | 0.4070 | 0.3976 |
| S44 | 0.3352 | 0.3255 | 0.3126 | 0.2788 | 0.2953 | 0.3143 | 0.3140 | 0.3744 | 0.4136 | 0.4154 | 0.4185 | 0.4141 | 0.3959 | 0.3785 | 0.3843 | 0.3933 | 0.3939 | 0.4057 | 0.3963 |
| S47 | 0.3262 | 0.3165 | 0.3036 | 0.2699 | 0.2863 | 0.3053 | 0.3050 | 0.3654 | 0.4047 | 0.4064 | 0.4095 | 0.4051 | 0.3869 | 0.3695 | 0.3753 | 0.3843 | 0.3849 | 0.3967 | 0.3873 |
| S56 | 0.3258 | 0.3161 | 0.3032 | 0.2695 | 0.2859 | 0.3049 | 0.3046 | 0.3650 | 0.4043 | 0.4060 | 0.4092 | 0.4048 | 0.3865 | 0.3691 | 0.3750 | 0.3839 | 0.3846 | 0.3963 | 0.3869 |
| S53 | 0.3259 | 0.3163 | 0.3033 | 0.2696 | 0.2860 | 0.3050 | 0.3048 | 0.3652 | 0.4044 | 0.4061 | 0.4093 | 0.4049 | 0.3866 | 0.3692 | 0.3751 | 0.3840 | 0.3847 | 0.3965 | 0.3870 |
| S60 | 0.3257 | 0.3160 | 0.3031 | 0.2694 | 0.2858 | 0.3048 | 0.3045 | 0.3649 | 0.4042 | 0.4059 | 0.4090 | 0.4046 | 0.3864 | 0.3690 | 0.3748 | 0.3838 | 0.3844 | 0.3962 | 0.3868 |
| S54 | 0.3281 | 0.3184 | 0.3055 | 0.2718 | 0.2882 | 0.3072 | 0.3069 | 0.3673 | 0.4066 | 0.4083 | 0.4115 | 0.4071 | 0.3888 | 0.3714 | 0.3772 | 0.3862 | 0.3869 | 0.3986 | 0.3892 |
| S80 | 0.3273 | 0.3176 | 0.3047 | 0.2710 | 0.2874 | 0.3064 | 0.3061 | 0.3665 | 0.4058 | 0.4075 | 0.4107 | 0.4063 | 0.3880 | 0.3706 | 0.3765 | 0.3854 | 0.3861 | 0.3978 | 0.3884 |
| S9 | 0.3154 | 0.3058 | 0.2929 | 0.2591 | 0.2755 | 0.2946 | 0.2943 | 0.3547 | 0.3939 | 0.3957 | 0.3988 | 0.3944 | 0.3761 | 0.3587 | 0.3646 | 0.3736 | 0.3742 | 0.3860 | 0.3765 |
| S86 | 0.3383 | 0.3286 | 0.3157 | 0.2820 | 0.2984 | 0.3174 | 0.3171 | 0.3775 | 0.4168 | 0.4185 | 0.4216 | 0.4172 | 0.3990 | 0.3816 | 0.3874 | 0.3964 | 0.3970 | 0.4088 | 0.3994 |
| S34 | 0.3238 | 0.3141 | 0.3012 | 0.2675 | 0.2839 | 0.3029 | 0.3026 | 0.3630 | 0.4023 | 0.4040 | 0.4072 | 0.4028 | 0.3845 | 0.3671 | 0.3730 | 0.3819 | 0.3826 | 0.3943 | 0.3849 |
| S84 | 0.3264 | 0.3167 | 0.3038 | 0.2700 | 0.2865 | 0.3055 | 0.3052 | 0.3656 | 0.4048 | 0.4066 | 0.4097 | 0.4053 | 0.3871 | 0.3696 | 0.3755 | 0.3845 | 0.3851 | 0.3969 | 0.3874 |
| S55 | 0.3273 | 0.3177 | 0.3047 | 0.2710 | 0.2874 | 0.3064 | 0.3062 | 0.3666 | 0.4058 | 0.4075 | 0.4107 | 0.4063 | 0.3880 | 0.3706 | 0.3765 | 0.3854 | 0.3861 | 0.3979 | 0.3884 |
| S52 | 0.3145 | 0.3048 | 0.2919 | 0.2582 | 0.2746 | 0.2936 | 0.2933 | 0.3537 | 0.3930 | 0.3947 | 0.3979 | 0.3935 | 0.3752 | 0.3578 | 0.3637 | 0.3726 | 0.3733 | 0.3850 | 0.3756 |
| S38 | 0.1984 | 0.1888 | 0.1758 | 0.1421 | 0.1585 | 0.1775 | 0.1773 | 0.2376 | 0.2769 | 0.2786 | 0.2818 | 0.2774 | 0.2591 | 0.2417 | 0.2476 | 0.2565 | 0.2572 | 0.2689 | 0.2595 |
| S101 | 0.1350 | 0.1253 | 0.1124 | 0.1006 | 0.1531 | 0.1721 | 0.1719 | 0.3204 | 0.3596 | 0.3614 | 0.3645 | 0.3601 | 0.3419 | 0.3244 | 0.3303 | 0.3393 | 0.3399 | 0.3517 | 0.3422 |
| S102 | 0.1365 | 0.1269 | 0.1140 | 0.1021 | 0.1546 | 0.1737 | 0.1734 | 0.3219 | 0.3612 | 0.3629 | 0.3661 | 0.3616 | 0.3434 | 0.3260 | 0.3318 | 0.3408 | 0.3414 | 0.3532 | 0.3438 |
| S85 | 0.1370 | 0.1274 | 0.1145 | 0.1026 | 0.1551 | 0.1742 | 0.1739 | 0.3224 | 0.3617 | 0.3634 | 0.3665 | 0.3621 | 0.3439 | 0.3265 | 0.3323 | 0.3413 | 0.3419 | 0.3537 | 0.3443 |
| S63 | 0.1481 | 0.1385 | 0.1255 | 0.1137 | 0.1662 | 0.1852 | 0.1850 | 0.3335 | 0.3728 | 0.3745 | 0.3776 | 0.3732 | 0.3550 | 0.3376 | 0.3434 | 0.3524 | 0.3530 | 0.3648 | 0.3554 |
| S72 | 0.1464 | 0.1368 | 0.1238 | 0.1120 | 0.1645 | 0.1835 | 0.1833 | 0.3318 | 0.3710 | 0.3728 | 0.3759 | 0.3715 | 0.3533 | 0.3359 | 0.3417 | 0.3507 | 0.3513 | 0.3631 | 0.3537 |
| S71 | 0.1407 | 0.1311 | 0.1181 | 0.1063 | 0.1588 | 0.1778 | 0.1776 | 0.3261 | 0.3654 | 0.3671 | 0.3702 | 0.3658 | 0.3476 | 0.3302 | 0.3360 | 0.3450 | 0.3456 | 0.3574 | 0.3480 |
| S2 | 0.0000 | 0.0303 | 0.0627 | 0.0784 | 0.1309 | 0.1500 | 0.1497 | 0.2982 | 0.3375 | 0.3392 | 0.3424 | 0.3379 | 0.3197 | 0.3023 | 0.3081 | 0.3171 | 0.3177 | 0.3295 | 0.3201 |
| S3 | 0.0303 | 0.0000 | 0.0531 | 0.0688 | 0.1213 | 0.1403 | 0.1400 | 0.2886 | 0.3278 | 0.3296 | 0.3327 | 0.3283 | 0.3100 | 0.2926 | 0.2985 | 0.3075 | 0.3081 | 0.3199 | 0.3104 |
| S26 | 0.0627 | 0.0531 | 0.0000 | 0.0558 | 0.1084 | 0.1274 | 0.1271 | 0.2757 | 0.3149 | 0.3166 | 0.3198 | 0.3154 | 0.2971 | 0.2797 | 0.2856 | 0.2945 | 0.2952 | 0.3070 | 0.2975 |
| S88 | 0.0784 | 0.0688 | 0.0558 | 0.0000 | 0.0746 | 0.0936 | 0.0934 | 0.2419 | 0.2811 | 0.2829 | 0.2860 | 0.2816 | 0.2634 | 0.2460 | 0.2518 | 0.2608 | 0.2614 | 0.2732 | 0.2638 |
| S104 | 0.1309 | 0.1213 | 0.1084 | 0.0746 | 0.0000 | 0.0530 | 0.0527 | 0.2583 | 0.2976 | 0.2993 | 0.3025 | 0.2980 | 0.2798 | 0.2624 | 0.2682 | 0.2772 | 0.2778 | 0.2896 | 0.2802 |
| S4 | 0.1500 | 0.1403 | 0.1274 | 0.0936 | 0.0530 | 0.0000 | 0.0372 | 0.2774 | 0.3166 | 0.3183 | 0.3215 | 0.3171 | 0.2988 | 0.2814 | 0.2873 | 0.2962 | 0.2969 | 0.3086 | 0.2992 |
| S6 | 0.1497 | 0.1400 | 0.1271 | 0.0934 | 0.0527 | 0.0372 | 0.0000 | 0.2771 | 0.3163 | 0.3181 | 0.3212 | 0.3168 | 0.2985 | 0.2811 | 0.2870 | 0.2960 | 0.2966 | 0.3084 | 0.2989 |
| S103 | 0.2982 | 0.2886 | 0.2757 | 0.2419 | 0.2583 | 0.2774 | 0.2771 | 0.0000 | 0.0758 | 0.0775 | 0.0807 | 0.0763 | 0.0580 | 0.0590 | 0.1156 | 0.1245 | 0.1252 | 0.1370 | 0.1275 |
| S7 | 0.3375 | 0.3278 | 0.3149 | 0.2811 | 0.2976 | 0.3166 | 0.3163 | 0.0758 | 0.0000 | 0.0440 | 0.0880 | 0.0835 | 0.0840 | 0.0982 | 0.1548 | 0.1638 | 0.1644 | 0.1762 | 0.1668 |
| S8 | 0.3392 | 0.3296 | 0.3166 | 0.2829 | 0.2993 | 0.3183 | 0.3181 | 0.0775 | 0.0440 | 0.0000 | 0.0897 | 0.0853 | 0.0857 | 0.1000 | 0.1566 | 0.1655 | 0.1662 | 0.1779 | 0.1685 |
| S76 | 0.3424 | 0.3327 | 0.3198 | 0.2860 | 0.3025 | 0.3215 | 0.3212 | 0.0807 | 0.0880 | 0.0897 | 0.0000 | 0.0385 | 0.0888 | 0.1031 | 0.1597 | 0.1687 | 0.1693 | 0.1811 | 0.1716 |
| S78 | 0.3379 | 0.3283 | 0.3154 | 0.2816 | 0.2980 | 0.3171 | 0.3168 | 0.0763 | 0.0835 | 0.0853 | 0.0385 | 0.0000 | 0.0844 | 0.0987 | 0.1553 | 0.1643 | 0.1649 | 0.1767 | 0.1672 |
| S77 | 0.3197 | 0.3100 | 0.2971 | 0.2634 | 0.2798 | 0.2988 | 0.2985 | 0.0580 | 0.0840 | 0.0857 | 0.0888 | 0.0844 | 0.0000 | 0.0804 | 0.1370 | 0.1460 | 0.1466 | 0.1584 | 0.1490 |
| S105 | 0.3023 | 0.2926 | 0.2797 | 0.2460 | 0.2624 | 0.2814 | 0.2811 | 0.0590 | 0.0982 | 0.1000 | 0.1031 | 0.0987 | 0.0804 | 0.0000 | 0.1196 | 0.1286 | 0.1292 | 0.1410 | 0.1316 |
| S15 | 0.3081 | 0.2985 | 0.2856 | 0.2518 | 0.2682 | 0.2873 | 0.2870 | 0.1156 | 0.1548 | 0.1566 | 0.1597 | 0.1553 | 0.1370 | 0.1196 | 0.0000 | 0.0761 | 0.0767 | 0.1206 | 0.1112 |
| S19 | 0.3171 | 0.3075 | 0.2945 | 0.2608 | 0.2772 | 0.2962 | 0.2960 | 0.1245 | 0.1638 | 0.1655 | 0.1687 | 0.1643 | 0.1460 | 0.1286 | 0.0761 | 0.0000 | 0.0496 | 0.1296 | 0.1201 |
| S20 | 0.3177 | 0.3081 | 0.2952 | 0.2614 | 0.2778 | 0.2969 | 0.2966 | 0.1252 | 0.1644 | 0.1662 | 0.1693 | 0.1649 | 0.1466 | 0.1292 | 0.0767 | 0.0496 | 0.0000 | 0.1302 | 0.1208 |
| S29 | 0.3295 | 0.3199 | 0.3070 | 0.2732 | 0.2896 | 0.3086 | 0.3084 | 0.1370 | 0.1762 | 0.1779 | 0.1811 | 0.1767 | 0.1584 | 0.1410 | 0.1206 | 0.1296 | 0.1302 | 0.0000 | 0.0689 |
| S31 | 0.3201 | 0.3104 | 0.2975 | 0.2638 | 0.2802 | 0.2992 | 0.2989 | 0.1275 | 0.1668 | 0.1685 | 0.1716 | 0.1672 | 0.1490 | 0.1316 | 0.1112 | 0.1201 | 0.1208 | 0.0689 | 0.0000 |
| S33 | 0.3144 | 0.3047 | 0.2918 | 0.2580 | 0.2745 | 0.2935 | 0.2932 | 0.1218 | 0.1610 | 0.1628 | 0.1659 | 0.1615 | 0.1432 | 0.1258 | 0.1054 | 0.1144 | 0.1150 | 0.0847 | 0.0753 |
| S35 | 0.3024 | 0.2927 | 0.2798 | 0.2460 | 0.2625 | 0.2815 | 0.2812 | 0.1098 | 0.1490 | 0.1508 | 0.1539 | 0.1495 | 0.1312 | 0.1138 | 0.0934 | 0.1024 | 0.1030 | 0.0965 | 0.0871 |
| S16 | 0.2610 | 0.2513 | 0.2384 | 0.2047 | 0.2211 | 0.2401 | 0.2398 | 0.0982 | 0.1375 | 0.1392 | 0.1423 | 0.1379 | 0.1197 | 0.1023 | 0.1081 | 0.1171 | 0.1177 | 0.1295 | 0.1201 |
| S17 | 0.2618 | 0.2521 | 0.2392 | 0.2054 | 0.2219 | 0.2409 | 0.2406 | 0.0990 | 0.1382 | 0.1400 | 0.1431 | 0.1387 | 0.1204 | 0.1030 | 0.1089 | 0.1179 | 0.1185 | 0.1303 | 0.1208 |
| S18 | 0.2563 | 0.2466 | 0.2337 | 0.1999 | 0.2164 | 0.2354 | 0.2351 | 0.0935 | 0.1327 | 0.1345 | 0.1376 | 0.1332 | 0.1149 | 0.0975 | 0.1034 | 0.1124 | 0.1130 | 0.1248 | 0.1153 |
| S30 | 0.2020 | 0.1924 | 0.1795 | 0.1457 | 0.1621 | 0.1812 | 0.1809 | 0.1402 | 0.1795 | 0.1812 | 0.1844 | 0.1800 | 0.1617 | 0.1443 | 0.1501 | 0.1591 | 0.1598 | 0.1715 | 0.1621 |
| S37 | 0.2756 | 0.2659 | 0.2530 | 0.2192 | 0.2357 | 0.2547 | 0.2544 | 0.2780 | 0.3172 | 0.3189 | 0.3221 | 0.3177 | 0.2994 | 0.2820 | 0.2879 | 0.2968 | 0.2975 | 0.3093 | 0.2998 |
| S49 | 0.2737 | 0.2640 | 0.2511 | 0.2174 | 0.2338 | 0.2528 | 0.2525 | 0.2761 | 0.3153 | 0.3171 | 0.3202 | 0.3158 | 0.2975 | 0.2801 | 0.2860 | 0.2950 | 0.2956 | 0.3074 | 0.2979 |
| S50 | 0.2848 | 0.2751 | 0.2622 | 0.2284 | 0.2449 | 0.2639 | 0.2636 | 0.2871 | 0.3264 | 0.3281 | 0.3313 | 0.3269 | 0.3086 | 0.2912 | 0.2971 | 0.3060 | 0.3067 | 0.3184 | 0.3090 |
| S39 | 0.2836 | 0.2740 | 0.2610 | 0.2273 | 0.2437 | 0.2627 | 0.2625 | 0.2860 | 0.3252 | 0.3270 | 0.3301 | 0.3257 | 0.3075 | 0.2901 | 0.2959 | 0.3049 | 0.3055 | 0.3173 | 0.3079 |
| S96 | 0.2982 | 0.2886 | 0.2757 | 0.2419 | 0.2583 | 0.2774 | 0.2771 | 0.3006 | 0.3399 | 0.3416 | 0.3448 | 0.3404 | 0.3221 | 0.3047 | 0.3106 | 0.3195 | 0.3202 | 0.3319 | 0.3225 |
| S98 | 0.3035 | 0.2938 | 0.2809 | 0.2471 | 0.2636 | 0.2826 | 0.2823 | 0.3059 | 0.3451 | 0.3468 | 0.3500 | 0.3456 | 0.3273 | 0.3099 | 0.3158 | 0.3247 | 0.3254 | 0.3372 | 0.3277 |
| S99 | 0.3062 | 0.2966 | 0.2837 | 0.2499 | 0.2663 | 0.2854 | 0.2851 | 0.3086 | 0.3479 | 0.3496 | 0.3528 | 0.3483 | 0.3301 | 0.3127 | 0.3185 | 0.3275 | 0.3281 | 0.3399 | 0.3305 |
| S5 | 0.2717 | 0.2620 | 0.2491 | 0.2154 | 0.2318 | 0.2508 | 0.2506 | 0.2741 | 0.3133 | 0.3151 | 0.3182 | 0.3138 | 0.2956 | 0.2782 | 0.2840 | 0.2930 | 0.2936 | 0.3054 | 0.2960 |
| S14 | 0.2215 | 0.2119 | 0.1989 | 0.1652 | 0.1816 | 0.2006 | 0.2004 | 0.2454 | 0.2846 | 0.2864 | 0.2895 | 0.2851 | 0.2668 | 0.2494 | 0.2553 | 0.2642 | 0.2649 | 0.2767 | 0.2672 |
| S61 | 0.2322 | 0.2226 | 0.2096 | 0.1759 | 0.1923 | 0.2113 | 0.2111 | 0.2561 | 0.2953 | 0.2970 | 0.3002 | 0.2958 | 0.2775 | 0.2601 | 0.2660 | 0.2749 | 0.2756 | 0.2874 | 0.2779 |
| S62 | 0.2347 | 0.2250 | 0.2121 | 0.1783 | 0.1948 | 0.2138 | 0.2135 | 0.2585 | 0.2977 | 0.2995 | 0.3026 | 0.2982 | 0.2800 | 0.2626 | 0.2684 | 0.2774 | 0.2780 | 0.2898 | 0.2804 |
| S11 | 0.3323 | 0.3227 | 0.3097 | 0.2760 | 0.2924 | 0.3114 | 0.3112 | 0.3562 | 0.3954 | 0.3972 | 0.4003 | 0.3959 | 0.3776 | 0.3602 | 0.3661 | 0.3750 | 0.3757 | 0.3875 | 0.3780 |
| S13 | 0.3379 | 0.3283 | 0.3153 | 0.2816 | 0.2980 | 0.3170 | 0.3168 | 0.3618 | 0.4010 | 0.4028 | 0.4059 | 0.4015 | 0.3832 | 0.3658 | 0.3717 | 0.3806 | 0.3813 | 0.3931 | 0.3836 |
| S65 | 0.3324 | 0.3228 | 0.3098 | 0.2761 | 0.2925 | 0.3115 | 0.3113 | 0.3563 | 0.3955 | 0.3972 | 0.4004 | 0.3960 | 0.3777 | 0.3603 | 0.3662 | 0.3751 | 0.3758 | 0.3876 | 0.3781 |
| S42 | 0.3241 | 0.3144 | 0.3015 | 0.2678 | 0.2842 | 0.3032 | 0.3029 | 0.3479 | 0.3872 | 0.3889 | 0.3921 | 0.3876 | 0.3694 | 0.3520 | 0.3578 | 0.3668 | 0.3674 | 0.3792 | 0.3698 |
| S57 | 0.3101 | 0.3004 | 0.2875 | 0.2537 | 0.2702 | 0.2892 | 0.2889 | 0.3339 | 0.3731 | 0.3749 | 0.3780 | 0.3736 | 0.3554 | 0.3380 | 0.3438 | 0.3528 | 0.3534 | 0.3652 | 0.3558 |
| S36 | 0.3010 | 0.2914 | 0.2784 | 0.2447 | 0.2611 | 0.2801 | 0.2799 | 0.3249 | 0.3641 | 0.3658 | 0.3690 | 0.3646 | 0.3463 | 0.3289 | 0.3348 | 0.3437 | 0.3444 | 0.3562 | 0.3467 |
| S89 | 0.3026 | 0.2929 | 0.2800 | 0.2463 | 0.2627 | 0.2817 | 0.2814 | 0.3264 | 0.3657 | 0.3674 | 0.3706 | 0.3662 | 0.3479 | 0.3305 | 0.3364 | 0.3453 | 0.3460 | 0.3577 | 0.3483 |
| S58 | 0.2720 | 0.2624 | 0.2495 | 0.2157 | 0.2321 | 0.2512 | 0.2509 | 0.2959 | 0.3351 | 0.3369 | 0.3400 | 0.3356 | 0.3174 | 0.2999 | 0.3058 | 0.3148 | 0.3154 | 0.3272 | 0.3177 |
| S59 | 0.2638 | 0.2541 | 0.2412 | 0.2075 | 0.2239 | 0.2429 | 0.2426 | 0.2876 | 0.3269 | 0.3286 | 0.3318 | 0.3274 | 0.3091 | 0.2917 | 0.2976 | 0.3065 | 0.3072 | 0.3189 | 0.3095 |
| S74 | 0.2751 | 0.2654 | 0.2525 | 0.2188 | 0.2352 | 0.2542 | 0.2540 | 0.2989 | 0.3382 | 0.3399 | 0.3431 | 0.3387 | 0.3204 | 0.3030 | 0.3089 | 0.3178 | 0.3185 | 0.3302 | 0.3208 |
| S75 | 0.2817 | 0.2720 | 0.2591 | 0.2253 | 0.2418 | 0.2608 | 0.2605 | 0.3055 | 0.3447 | 0.3465 | 0.3496 | 0.3452 | 0.3270 | 0.3096 | 0.3154 | 0.3244 | 0.3250 | 0.3368 | 0.3274 |
| S27 | 0.2175 | 0.2079 | 0.1949 | 0.1612 | 0.1776 | 0.1966 | 0.1964 | 0.2414 | 0.2806 | 0.2824 | 0.2855 | 0.2811 | 0.2628 | 0.2454 | 0.2513 | 0.2602 | 0.2609 | 0.2727 | 0.2632 |
| S28 | 0.2389 | 0.2293 | 0.2164 | 0.1826 | 0.1990 | 0.2181 | 0.2178 | 0.2628 | 0.3020 | 0.3038 | 0.3069 | 0.3025 | 0.2842 | 0.2668 | 0.2727 | 0.2817 | 0.2823 | 0.2941 | 0.2846 |
| S90 | 0.2796 | 0.2699 | 0.2570 | 0.2233 | 0.2397 | 0.2587 | 0.2585 | 0.3034 | 0.3427 | 0.3444 | 0.3476 | 0.3432 | 0.3249 | 0.3075 | 0.3134 | 0.3223 | 0.3230 | 0.3347 | 0.3253 |
| S91 | 0.2946 | 0.2849 | 0.2720 | 0.2383 | 0.2547 | 0.2737 | 0.2734 | 0.3184 | 0.3577 | 0.3594 | 0.3626 | 0.3582 | 0.3399 | 0.3225 | 0.3284 | 0.3373 | 0.3380 | 0.3497 | 0.3403 |
| S95 | 0.2987 | 0.2891 | 0.2761 | 0.2424 | 0.2588 | 0.2778 | 0.2776 | 0.3226 | 0.3618 | 0.3636 | 0.3667 | 0.3623 | 0.3440 | 0.3266 | 0.3325 | 0.3415 | 0.3421 | 0.3539 | 0.3444 |
| S93 | 0.2901 | 0.2804 | 0.2675 | 0.2337 | 0.2502 | 0.2692 | 0.2689 | 0.3139 | 0.3532 | 0.3549 | 0.3580 | 0.3536 | 0.3354 | 0.3180 | 0.3238 | 0.3328 | 0.3334 | 0.3452 | 0.3358 |
| S92 | 0.2825 | 0.2728 | 0.2599 | 0.2261 | 0.2426 | 0.2616 | 0.2613 | 0.3063 | 0.3455 | 0.3473 | 0.3504 | 0.3460 | 0.3278 | 0.3104 | 0.3162 | 0.3252 | 0.3258 | 0.3376 | 0.3282 |

Table S5 (Continued)

| Sample ID | S33 | S35 | S16 | S17 | S18 | S30 | S37 | S49 | S50 | S39 | S96 | S98 | S99 | S5 | S14 | S61 | S62 | S11 | S13 |
| --- | --- | --- | --- | --- | --- | --- | --- | --- | --- | --- | --- | --- | --- | --- | --- | --- | --- | --- | --- |
| S1 | 0.3129 | 0.3009 | 0.2596 | 0.2603 | 0.2548 | 0.2006 | 0.2741 | 0.2723 | 0.2833 | 0.2822 | 0.2968 | 0.3020 | 0.3048 | 0.2703 | 0.2201 | 0.2308 | 0.2332 | 0.3309 | 0.3365 |
| S48 | 0.3127 | 0.3007 | 0.2594 | 0.2601 | 0.2546 | 0.2004 | 0.2739 | 0.2720 | 0.2831 | 0.2820 | 0.2966 | 0.3018 | 0.3046 | 0.2701 | 0.2199 | 0.2306 | 0.2330 | 0.3307 | 0.3363 |
| S21 | 0.3127 | 0.3007 | 0.2594 | 0.2601 | 0.2546 | 0.2004 | 0.2739 | 0.2721 | 0.2831 | 0.2820 | 0.2966 | 0.3018 | 0.3046 | 0.2701 | 0.2199 | 0.2306 | 0.2330 | 0.3307 | 0.3363 |
| S73 | 0.3136 | 0.3016 | 0.2602 | 0.2610 | 0.2555 | 0.2013 | 0.2748 | 0.2729 | 0.2840 | 0.2829 | 0.2975 | 0.3027 | 0.3055 | 0.2709 | 0.2208 | 0.2315 | 0.2339 | 0.3316 | 0.3372 |
| S97 | 0.3100 | 0.2980 | 0.2567 | 0.2575 | 0.2520 | 0.1977 | 0.2713 | 0.2694 | 0.2804 | 0.2793 | 0.2939 | 0.2992 | 0.3019 | 0.2674 | 0.2172 | 0.2279 | 0.2304 | 0.3280 | 0.3336 |
| S40 | 0.3494 | 0.3373 | 0.2960 | 0.2968 | 0.2913 | 0.2370 | 0.3106 | 0.3087 | 0.3197 | 0.3186 | 0.3332 | 0.3385 | 0.3412 | 0.3067 | 0.2565 | 0.2672 | 0.2697 | 0.3673 | 0.3729 |
| S41 | 0.3559 | 0.3439 | 0.3025 | 0.3033 | 0.2978 | 0.2436 | 0.3171 | 0.3152 | 0.3263 | 0.3251 | 0.3398 | 0.3450 | 0.3478 | 0.3132 | 0.2630 | 0.2737 | 0.2762 | 0.3738 | 0.3794 |
| S87 | 0.3507 | 0.3387 | 0.2973 | 0.2981 | 0.2926 | 0.2384 | 0.3119 | 0.3100 | 0.3211 | 0.3200 | 0.3346 | 0.3398 | 0.3426 | 0.3081 | 0.2579 | 0.2686 | 0.2710 | 0.3687 | 0.3743 |
| S10 | 0.4090 | 0.3970 | 0.3557 | 0.3564 | 0.3509 | 0.2967 | 0.3702 | 0.3684 | 0.3794 | 0.3783 | 0.3929 | 0.3981 | 0.4009 | 0.3664 | 0.3162 | 0.3269 | 0.3293 | 0.4270 | 0.4326 |
| S51 | 0.4166 | 0.4046 | 0.3633 | 0.3640 | 0.3585 | 0.3043 | 0.3778 | 0.3759 | 0.3870 | 0.3859 | 0.4005 | 0.4057 | 0.4085 | 0.3740 | 0.3238 | 0.3345 | 0.3369 | 0.4346 | 0.4402 |
| S79 | 0.4031 | 0.3911 | 0.3497 | 0.3505 | 0.3450 | 0.2908 | 0.3643 | 0.3624 | 0.3735 | 0.3723 | 0.3870 | 0.3922 | 0.3950 | 0.3604 | 0.3102 | 0.3209 | 0.3234 | 0.4210 | 0.4266 |
| S23 | 0.4097 | 0.3977 | 0.3564 | 0.3571 | 0.3516 | 0.2974 | 0.3709 | 0.3691 | 0.3801 | 0.3790 | 0.3936 | 0.3988 | 0.4016 | 0.3671 | 0.3169 | 0.3276 | 0.3300 | 0.4277 | 0.4333 |
| S45 | 0.4107 | 0.3987 | 0.3574 | 0.3581 | 0.3526 | 0.2984 | 0.3719 | 0.3700 | 0.3811 | 0.3800 | 0.3946 | 0.3998 | 0.4026 | 0.3681 | 0.3179 | 0.3286 | 0.3310 | 0.4287 | 0.4343 |
| S46 | 0.4058 | 0.3938 | 0.3524 | 0.3532 | 0.3477 | 0.2935 | 0.3670 | 0.3651 | 0.3762 | 0.3750 | 0.3897 | 0.3949 | 0.3976 | 0.3631 | 0.3129 | 0.3236 | 0.3261 | 0.4237 | 0.4293 |
| S24 | 0.4045 | 0.3925 | 0.3512 | 0.3520 | 0.3464 | 0.2922 | 0.3658 | 0.3639 | 0.3749 | 0.3738 | 0.3884 | 0.3937 | 0.3964 | 0.3619 | 0.3117 | 0.3224 | 0.3248 | 0.4225 | 0.4281 |
| S32 | 0.4073 | 0.3953 | 0.3539 | 0.3547 | 0.3492 | 0.2950 | 0.3685 | 0.3666 | 0.3777 | 0.3766 | 0.3912 | 0.3964 | 0.3992 | 0.3647 | 0.3145 | 0.3252 | 0.3276 | 0.4253 | 0.4309 |
| S12 | 0.3747 | 0.3627 | 0.3213 | 0.3221 | 0.3166 | 0.2623 | 0.3359 | 0.3340 | 0.3451 | 0.3439 | 0.3585 | 0.3638 | 0.3665 | 0.3320 | 0.2818 | 0.2925 | 0.2950 | 0.3926 | 0.3982 |
| S22 | 0.3919 | 0.3798 | 0.3385 | 0.3393 | 0.3338 | 0.2795 | 0.3531 | 0.3512 | 0.3622 | 0.3611 | 0.3757 | 0.3810 | 0.3837 | 0.3492 | 0.2990 | 0.3097 | 0.3122 | 0.4098 | 0.4154 |
| S44 | 0.3905 | 0.3785 | 0.3372 | 0.3379 | 0.3324 | 0.2782 | 0.3517 | 0.3499 | 0.3609 | 0.3598 | 0.3744 | 0.3796 | 0.3824 | 0.3479 | 0.2977 | 0.3084 | 0.3108 | 0.4085 | 0.4141 |
| S47 | 0.3815 | 0.3695 | 0.3282 | 0.3290 | 0.3234 | 0.2692 | 0.3428 | 0.3409 | 0.3519 | 0.3508 | 0.3654 | 0.3706 | 0.3734 | 0.3389 | 0.2887 | 0.2994 | 0.3018 | 0.3995 | 0.4051 |
| S56 | 0.3812 | 0.3692 | 0.3278 | 0.3286 | 0.3231 | 0.2689 | 0.3424 | 0.3405 | 0.3516 | 0.3504 | 0.3651 | 0.3703 | 0.3730 | 0.3385 | 0.2883 | 0.2990 | 0.3015 | 0.3991 | 0.4047 |
| S53 | 0.3813 | 0.3693 | 0.3279 | 0.3287 | 0.3232 | 0.2690 | 0.3425 | 0.3406 | 0.3517 | 0.3505 | 0.3652 | 0.3704 | 0.3732 | 0.3386 | 0.2885 | 0.2991 | 0.3016 | 0.3993 | 0.4049 |
| S60 | 0.3810 | 0.3690 | 0.3277 | 0.3285 | 0.3229 | 0.2687 | 0.3423 | 0.3404 | 0.3514 | 0.3503 | 0.3649 | 0.3702 | 0.3729 | 0.3384 | 0.2882 | 0.2989 | 0.3013 | 0.3990 | 0.4046 |
| S54 | 0.3835 | 0.3715 | 0.3301 | 0.3309 | 0.3254 | 0.2711 | 0.3447 | 0.3428 | 0.3539 | 0.3527 | 0.3673 | 0.3726 | 0.3753 | 0.3408 | 0.2906 | 0.3013 | 0.3038 | 0.4014 | 0.4070 |
| S80 | 0.3827 | 0.3707 | 0.3293 | 0.3301 | 0.3246 | 0.2704 | 0.3439 | 0.3420 | 0.3531 | 0.3519 | 0.3666 | 0.3718 | 0.3745 | 0.3400 | 0.2898 | 0.3005 | 0.3030 | 0.4006 | 0.4062 |
| S9 | 0.3708 | 0.3588 | 0.3174 | 0.3182 | 0.3127 | 0.2585 | 0.3320 | 0.3301 | 0.3412 | 0.3401 | 0.3547 | 0.3599 | 0.3627 | 0.3281 | 0.2780 | 0.2887 | 0.2911 | 0.3888 | 0.3944 |
| S86 | 0.3936 | 0.3816 | 0.3403 | 0.3411 | 0.3355 | 0.2813 | 0.3549 | 0.3530 | 0.3640 | 0.3629 | 0.3775 | 0.3827 | 0.3855 | 0.3510 | 0.3008 | 0.3115 | 0.3139 | 0.4116 | 0.4172 |
| S34 | 0.3792 | 0.3672 | 0.3258 | 0.3266 | 0.3211 | 0.2669 | 0.3404 | 0.3385 | 0.3496 | 0.3484 | 0.3631 | 0.3683 | 0.3710 | 0.3365 | 0.2863 | 0.2970 | 0.2995 | 0.3971 | 0.4027 |
| S84 | 0.3817 | 0.3697 | 0.3284 | 0.3291 | 0.3236 | 0.2694 | 0.3429 | 0.3410 | 0.3521 | 0.3510 | 0.3656 | 0.3708 | 0.3736 | 0.3391 | 0.2889 | 0.2996 | 0.3020 | 0.3997 | 0.4053 |
| S55 | 0.3827 | 0.3707 | 0.3293 | 0.3301 | 0.3246 | 0.2704 | 0.3439 | 0.3420 | 0.3531 | 0.3519 | 0.3666 | 0.3718 | 0.3746 | 0.3400 | 0.2898 | 0.3005 | 0.3030 | 0.4006 | 0.4062 |
| S52 | 0.3699 | 0.3579 | 0.3165 | 0.3173 | 0.3118 | 0.2576 | 0.3311 | 0.3292 | 0.3403 | 0.3391 | 0.3538 | 0.3590 | 0.3617 | 0.3272 | 0.2770 | 0.2877 | 0.2902 | 0.3878 | 0.3934 |
| S38 | 0.2538 | 0.2418 | 0.2004 | 0.2012 | 0.1957 | 0.1415 | 0.2150 | 0.2131 | 0.2242 | 0.2230 | 0.2377 | 0.2429 | 0.2457 | 0.2111 | 0.1609 | 0.1716 | 0.1741 | 0.2717 | 0.2773 |
| S101 | 0.3365 | 0.3245 | 0.2832 | 0.2839 | 0.2784 | 0.2242 | 0.2977 | 0.2958 | 0.3069 | 0.3058 | 0.3204 | 0.3256 | 0.3284 | 0.2939 | 0.2437 | 0.2544 | 0.2568 | 0.3545 | 0.3601 |
| S102 | 0.3381 | 0.3260 | 0.2847 | 0.2855 | 0.2800 | 0.2257 | 0.2993 | 0.2974 | 0.3084 | 0.3073 | 0.3219 | 0.3272 | 0.3299 | 0.2954 | 0.2452 | 0.2559 | 0.2584 | 0.3560 | 0.3616 |
| S85 | 0.3385 | 0.3265 | 0.2852 | 0.2860 | 0.2804 | 0.2262 | 0.2998 | 0.2979 | 0.3089 | 0.3078 | 0.3224 | 0.3277 | 0.3304 | 0.2959 | 0.2457 | 0.2564 | 0.2588 | 0.3565 | 0.3621 |
| S63 | 0.3496 | 0.3376 | 0.2963 | 0.2970 | 0.2915 | 0.2373 | 0.3109 | 0.3090 | 0.3200 | 0.3189 | 0.3335 | 0.3387 | 0.3415 | 0.3070 | 0.2568 | 0.2675 | 0.2699 | 0.3676 | 0.3732 |
| S72 | 0.3479 | 0.3359 | 0.2946 | 0.2953 | 0.2898 | 0.2356 | 0.3091 | 0.3073 | 0.3183 | 0.3172 | 0.3318 | 0.3370 | 0.3398 | 0.3053 | 0.2551 | 0.2658 | 0.2682 | 0.3659 | 0.3715 |
| S71 | 0.3422 | 0.3302 | 0.2889 | 0.2896 | 0.2841 | 0.2299 | 0.3035 | 0.3016 | 0.3126 | 0.3115 | 0.3261 | 0.3313 | 0.3341 | 0.2996 | 0.2494 | 0.2601 | 0.2625 | 0.3602 | 0.3658 |
| S2 | 0.3144 | 0.3024 | 0.2610 | 0.2618 | 0.2563 | 0.2020 | 0.2756 | 0.2737 | 0.2848 | 0.2836 | 0.2982 | 0.3035 | 0.3062 | 0.2717 | 0.2215 | 0.2322 | 0.2347 | 0.3323 | 0.3379 |
| S3 | 0.3047 | 0.2927 | 0.2513 | 0.2521 | 0.2466 | 0.1924 | 0.2659 | 0.2640 | 0.2751 | 0.2740 | 0.2886 | 0.2938 | 0.2966 | 0.2620 | 0.2119 | 0.2226 | 0.2250 | 0.3227 | 0.3283 |
| S26 | 0.2918 | 0.2798 | 0.2384 | 0.2392 | 0.2337 | 0.1795 | 0.2530 | 0.2511 | 0.2622 | 0.2610 | 0.2757 | 0.2809 | 0.2837 | 0.2491 | 0.1989 | 0.2096 | 0.2121 | 0.3097 | 0.3153 |
| S88 | 0.2580 | 0.2460 | 0.2047 | 0.2054 | 0.1999 | 0.1457 | 0.2192 | 0.2174 | 0.2284 | 0.2273 | 0.2419 | 0.2471 | 0.2499 | 0.2154 | 0.1652 | 0.1759 | 0.1783 | 0.2760 | 0.2816 |
| S104 | 0.2745 | 0.2625 | 0.2211 | 0.2219 | 0.2164 | 0.1621 | 0.2357 | 0.2338 | 0.2449 | 0.2437 | 0.2583 | 0.2636 | 0.2663 | 0.2318 | 0.1816 | 0.1923 | 0.1948 | 0.2924 | 0.2980 |
| S4 | 0.2935 | 0.2815 | 0.2401 | 0.2409 | 0.2354 | 0.1812 | 0.2547 | 0.2528 | 0.2639 | 0.2627 | 0.2774 | 0.2826 | 0.2854 | 0.2508 | 0.2006 | 0.2113 | 0.2138 | 0.3114 | 0.3170 |
| S6 | 0.2932 | 0.2812 | 0.2398 | 0.2406 | 0.2351 | 0.1809 | 0.2544 | 0.2525 | 0.2636 | 0.2625 | 0.2771 | 0.2823 | 0.2851 | 0.2506 | 0.2004 | 0.2111 | 0.2135 | 0.3112 | 0.3168 |
| S103 | 0.1218 | 0.1098 | 0.0982 | 0.0990 | 0.0935 | 0.1402 | 0.2780 | 0.2761 | 0.2871 | 0.2860 | 0.3006 | 0.3059 | 0.3086 | 0.2741 | 0.2454 | 0.2561 | 0.2585 | 0.3562 | 0.3618 |
| S7 | 0.1610 | 0.1490 | 0.1375 | 0.1382 | 0.1327 | 0.1795 | 0.3172 | 0.3153 | 0.3264 | 0.3252 | 0.3399 | 0.3451 | 0.3479 | 0.3133 | 0.2846 | 0.2953 | 0.2977 | 0.3954 | 0.4010 |
| S8 | 0.1628 | 0.1508 | 0.1392 | 0.1400 | 0.1345 | 0.1812 | 0.3189 | 0.3171 | 0.3281 | 0.3270 | 0.3416 | 0.3468 | 0.3496 | 0.3151 | 0.2864 | 0.2970 | 0.2995 | 0.3972 | 0.4028 |
| S76 | 0.1659 | 0.1539 | 0.1423 | 0.1431 | 0.1376 | 0.1844 | 0.3221 | 0.3202 | 0.3313 | 0.3301 | 0.3448 | 0.3500 | 0.3528 | 0.3182 | 0.2895 | 0.3002 | 0.3026 | 0.4003 | 0.4059 |
| S78 | 0.1615 | 0.1495 | 0.1379 | 0.1387 | 0.1332 | 0.1800 | 0.3177 | 0.3158 | 0.3269 | 0.3257 | 0.3404 | 0.3456 | 0.3483 | 0.3138 | 0.2851 | 0.2958 | 0.2982 | 0.3959 | 0.4015 |
| S77 | 0.1432 | 0.1312 | 0.1197 | 0.1204 | 0.1149 | 0.1617 | 0.2994 | 0.2975 | 0.3086 | 0.3075 | 0.3221 | 0.3273 | 0.3301 | 0.2956 | 0.2668 | 0.2775 | 0.2800 | 0.3776 | 0.3832 |
| S105 | 0.1258 | 0.1138 | 0.1023 | 0.1030 | 0.0975 | 0.1443 | 0.2820 | 0.2801 | 0.2912 | 0.2901 | 0.3047 | 0.3099 | 0.3127 | 0.2782 | 0.2494 | 0.2601 | 0.2626 | 0.3602 | 0.3658 |
| S15 | 0.1054 | 0.0934 | 0.1081 | 0.1089 | 0.1034 | 0.1501 | 0.2879 | 0.2860 | 0.2971 | 0.2959 | 0.3106 | 0.3158 | 0.3185 | 0.2840 | 0.2553 | 0.2660 | 0.2684 | 0.3661 | 0.3717 |
| S19 | 0.1144 | 0.1024 | 0.1171 | 0.1179 | 0.1124 | 0.1591 | 0.2968 | 0.2950 | 0.3060 | 0.3049 | 0.3195 | 0.3247 | 0.3275 | 0.2930 | 0.2642 | 0.2749 | 0.2774 | 0.3750 | 0.3806 |
| S20 | 0.1150 | 0.1030 | 0.1177 | 0.1185 | 0.1130 | 0.1598 | 0.2975 | 0.2956 | 0.3067 | 0.3055 | 0.3202 | 0.3254 | 0.3281 | 0.2936 | 0.2649 | 0.2756 | 0.2780 | 0.3757 | 0.3813 |
| S29 | 0.0847 | 0.0965 | 0.1295 | 0.1303 | 0.1248 | 0.1715 | 0.3093 | 0.3074 | 0.3184 | 0.3173 | 0.3319 | 0.3372 | 0.3399 | 0.3054 | 0.2767 | 0.2874 | 0.2898 | 0.3875 | 0.3931 |
| S31 | 0.0753 | 0.0871 | 0.1201 | 0.1208 | 0.1153 | 0.1621 | 0.2998 | 0.2979 | 0.3090 | 0.3079 | 0.3225 | 0.3277 | 0.3305 | 0.2960 | 0.2672 | 0.2779 | 0.2804 | 0.3780 | 0.3836 |
| S33 | 0.0000 | 0.0813 | 0.1143 | 0.1151 | 0.1096 | 0.1564 | 0.2941 | 0.2922 | 0.3033 | 0.3021 | 0.3168 | 0.3220 | 0.3248 | 0.2902 | 0.2615 | 0.2722 | 0.2746 | 0.3723 | 0.3779 |
| S35 | 0.0813 | 0.0000 | 0.1023 | 0.1031 | 0.0976 | 0.1444 | 0.2821 | 0.2802 | 0.2913 | 0.2901 | 0.3048 | 0.3100 | 0.3128 | 0.2782 | 0.2495 | 0.2602 | 0.2626 | 0.3603 | 0.3659 |
| S16 | 0.1143 | 0.1023 | 0.0000 | 0.0226 | 0.0274 | 0.1030 | 0.2407 | 0.2388 | 0.2499 | 0.2488 | 0.2634 | 0.2686 | 0.2714 | 0.2369 | 0.2081 | 0.2188 | 0.2213 | 0.3189 | 0.3245 |
| S17 | 0.1151 | 0.1031 | 0.0226 | 0.0000 | 0.0281 | 0.1038 | 0.2415 | 0.2396 | 0.2507 | 0.2495 | 0.2642 | 0.2694 | 0.2722 | 0.2376 | 0.2089 | 0.2196 | 0.2220 | 0.3197 | 0.3253 |
| S18 | 0.1096 | 0.0976 | 0.0274 | 0.0281 | 0.0000 | 0.0983 | 0.2360 | 0.2341 | 0.2452 | 0.2440 | 0.2587 | 0.2639 | 0.2667 | 0.2321 | 0.2034 | 0.2141 | 0.2165 | 0.3142 | 0.3198 |
| S30 | 0.1564 | 0.1444 | 0.1030 | 0.1038 | 0.0983 | 0.0000 | 0.1818 | 0.1799 | 0.1910 | 0.1898 | 0.2045 | 0.2097 | 0.2124 | 0.1779 | 0.1492 | 0.1599 | 0.1623 | 0.2600 | 0.2656 |
| S37 | 0.2941 | 0.2821 | 0.2407 | 0.2415 | 0.2360 | 0.1818 | 0.0000 | 0.0628 | 0.0738 | 0.0973 | 0.1120 | 0.1172 | 0.1200 | 0.0854 | 0.2227 | 0.2334 | 0.2358 | 0.3335 | 0.3391 |
| S49 | 0.2922 | 0.2802 | 0.2388 | 0.2396 | 0.2341 | 0.1799 | 0.0628 | 0.0000 | 0.0252 | 0.0954 | 0.1101 | 0.1153 | 0.1181 | 0.0835 | 0.2208 | 0.2315 | 0.2340 | 0.3316 | 0.3372 |
| S50 | 0.3033 | 0.2913 | 0.2499 | 0.2507 | 0.2452 | 0.1910 | 0.0738 | 0.0252 | 0.0000 | 0.1065 | 0.1212 | 0.1264 | 0.1291 | 0.0946 | 0.2319 | 0.2426 | 0.2450 | 0.3427 | 0.3483 |
| S39 | 0.3021 | 0.2901 | 0.2488 | 0.2495 | 0.2440 | 0.1898 | 0.0973 | 0.0954 | 0.1065 | 0.0000 | 0.0614 | 0.0666 | 0.0694 | 0.0562 | 0.2307 | 0.2414 | 0.2439 | 0.3415 | 0.3471 |
| S96 | 0.3168 | 0.3048 | 0.2634 | 0.2642 | 0.2587 | 0.2045 | 0.1120 | 0.1101 | 0.1212 | 0.0614 | 0.0000 | 0.0357 | 0.0385 | 0.0709 | 0.2454 | 0.2561 | 0.2585 | 0.3562 | 0.3618 |
| S98 | 0.3220 | 0.3100 | 0.2686 | 0.2694 | 0.2639 | 0.2097 | 0.1172 | 0.1153 | 0.1264 | 0.0666 | 0.0357 | 0.0000 | 0.0325 | 0.0761 | 0.2506 | 0.2613 | 0.2637 | 0.3614 | 0.3670 |
| S99 | 0.3248 | 0.3128 | 0.2714 | 0.2722 | 0.2667 | 0.2124 | 0.1200 | 0.1181 | 0.1291 | 0.0694 | 0.0385 | 0.0325 | 0.0000 | 0.0789 | 0.2534 | 0.2641 | 0.2665 | 0.3642 | 0.3698 |
| S5 | 0.2902 | 0.2782 | 0.2369 | 0.2376 | 0.2321 | 0.1779 | 0.0854 | 0.0835 | 0.0946 | 0.0562 | 0.0709 | 0.0761 | 0.0789 | 0.0000 | 0.2188 | 0.2295 | 0.2320 | 0.3296 | 0.3352 |
| S14 | 0.2615 | 0.2495 | 0.2081 | 0.2089 | 0.2034 | 0.1492 | 0.2227 | 0.2208 | 0.2319 | 0.2307 | 0.2454 | 0.2506 | 0.2534 | 0.2188 | 0.0000 | 0.0352 | 0.0572 | 0.2546 | 0.2602 |
| S61 | 0.2722 | 0.2602 | 0.2188 | 0.2196 | 0.2141 | 0.1599 | 0.2334 | 0.2315 | 0.2426 | 0.2414 | 0.2561 | 0.2613 | 0.2641 | 0.2295 | 0.0352 | 0.0000 | 0.0679 | 0.2653 | 0.2709 |
| S62 | 0.2746 | 0.2626 | 0.2213 | 0.2220 | 0.2165 | 0.1623 | 0.2358 | 0.2340 | 0.2450 | 0.2439 | 0.2585 | 0.2637 | 0.2665 | 0.2320 | 0.0572 | 0.0679 | 0.0000 | 0.2677 | 0.2733 |
| S11 | 0.3723 | 0.3603 | 0.3189 | 0.3197 | 0.3142 | 0.2600 | 0.3335 | 0.3316 | 0.3427 | 0.3415 | 0.3562 | 0.3614 | 0.3642 | 0.3296 | 0.2546 | 0.2653 | 0.2677 | 0.0000 | 0.0341 |
| S13 | 0.3779 | 0.3659 | 0.3245 | 0.3253 | 0.3198 | 0.2656 | 0.3391 | 0.3372 | 0.3483 | 0.3471 | 0.3618 | 0.3670 | 0.3698 | 0.3352 | 0.2602 | 0.2709 | 0.2733 | 0.0341 | 0.0000 |
| S65 | 0.3724 | 0.3604 | 0.3190 | 0.3198 | 0.3143 | 0.2601 | 0.3336 | 0.3317 | 0.3428 | 0.3416 | 0.3563 | 0.3615 | 0.3643 | 0.3297 | 0.2547 | 0.2654 | 0.2678 | 0.0286 | 0.0212 |
| S42 | 0.3641 | 0.3521 | 0.3107 | 0.3115 | 0.3060 | 0.2517 | 0.3253 | 0.3234 | 0.3344 | 0.3333 | 0.3479 | 0.3532 | 0.3559 | 0.3214 | 0.2464 | 0.2571 | 0.2595 | 0.0309 | 0.0365 |
| S57 | 0.3500 | 0.3380 | 0.2967 | 0.2974 | 0.2919 | 0.2377 | 0.3112 | 0.3094 | 0.3204 | 0.3193 | 0.3339 | 0.3391 | 0.3419 | 0.3074 | 0.2323 | 0.2430 | 0.2455 | 0.0778 | 0.0834 |
| S36 | 0.3410 | 0.3290 | 0.2876 | 0.2884 | 0.2829 | 0.2287 | 0.3022 | 0.3003 | 0.3114 | 0.3102 | 0.3249 | 0.3301 | 0.3329 | 0.2983 | 0.2233 | 0.2340 | 0.2364 | 0.1842 | 0.1898 |
| S89 | 0.3426 | 0.3306 | 0.2892 | 0.2900 | 0.2845 | 0.2303 | 0.3038 | 0.3019 | 0.3130 | 0.3118 | 0.3265 | 0.3317 | 0.3344 | 0.2999 | 0.2249 | 0.2356 | 0.2380 | 0.1858 | 0.1914 |
| S58 | 0.3120 | 0.3000 | 0.2587 | 0.2594 | 0.2539 | 0.1997 | 0.2732 | 0.2713 | 0.2824 | 0.2813 | 0.2959 | 0.3011 | 0.3039 | 0.2694 | 0.1943 | 0.2050 | 0.2075 | 0.1904 | 0.1960 |
| S59 | 0.3038 | 0.2918 | 0.2504 | 0.2512 | 0.2457 | 0.1914 | 0.2650 | 0.2631 | 0.2742 | 0.2730 | 0.2877 | 0.2929 | 0.2956 | 0.2611 | 0.1861 | 0.1968 | 0.1992 | 0.1822 | 0.1878 |
| S74 | 0.3151 | 0.3031 | 0.2617 | 0.2625 | 0.2570 | 0.2028 | 0.2763 | 0.2744 | 0.2855 | 0.2843 | 0.2990 | 0.3042 | 0.3070 | 0.2724 | 0.1974 | 0.2081 | 0.2105 | 0.1935 | 0.1991 |
| S75 | 0.3216 | 0.3096 | 0.2683 | 0.2690 | 0.2635 | 0.2093 | 0.2828 | 0.2810 | 0.2920 | 0.2909 | 0.3055 | 0.3107 | 0.3135 | 0.2790 | 0.2039 | 0.2146 | 0.2171 | 0.2001 | 0.2057 |
| S27 | 0.2575 | 0.2455 | 0.2041 | 0.2049 | 0.1994 | 0.1452 | 0.2187 | 0.2168 | 0.2279 | 0.2267 | 0.2414 | 0.2466 | 0.2494 | 0.2148 | 0.1398 | 0.1505 | 0.1529 | 0.1935 | 0.1991 |
| S28 | 0.2789 | 0.2669 | 0.2255 | 0.2263 | 0.2208 | 0.1666 | 0.2401 | 0.2382 | 0.2493 | 0.2482 | 0.2628 | 0.2680 | 0.2708 | 0.2363 | 0.1612 | 0.1719 | 0.1744 | 0.2149 | 0.2205 |
| S90 | 0.3196 | 0.3076 | 0.2662 | 0.2670 | 0.2615 | 0.2073 | 0.2808 | 0.2789 | 0.2900 | 0.2888 | 0.3035 | 0.3087 | 0.3115 | 0.2769 | 0.2019 | 0.2126 | 0.2150 | 0.2917 | 0.2973 |
| S91 | 0.3346 | 0.3226 | 0.2812 | 0.2820 | 0.2765 | 0.2223 | 0.2958 | 0.2939 | 0.3050 | 0.3038 | 0.3185 | 0.3237 | 0.3264 | 0.2919 | 0.2169 | 0.2276 | 0.2300 | 0.3067 | 0.3123 |
| S95 | 0.3387 | 0.3267 | 0.2853 | 0.2861 | 0.2806 | 0.2264 | 0.2999 | 0.2980 | 0.3091 | 0.3079 | 0.3226 | 0.3278 | 0.3306 | 0.2960 | 0.2210 | 0.2317 | 0.2342 | 0.3109 | 0.3165 |
| S93 | 0.3300 | 0.3180 | 0.2767 | 0.2775 | 0.2719 | 0.2177 | 0.2913 | 0.2894 | 0.3004 | 0.2993 | 0.3139 | 0.3192 | 0.3219 | 0.2874 | 0.2124 | 0.2230 | 0.2255 | 0.3022 | 0.3078 |
| S92 | 0.3224 | 0.3104 | 0.2691 | 0.2698 | 0.2643 | 0.2101 | 0.2836 | 0.2818 | 0.2928 | 0.2917 | 0.3063 | 0.3115 | 0.3143 | 0.2798 | 0.2047 | 0.2154 | 0.2179 | 0.2946 | 0.3002 |

Table S5 (Continued)

| Sample ID | S65 | S42 | S57 | S36 | S89 | S58 | S59 | S74 | S75 | S27 | S28 | S90 | S91 | S95 | S93 | S92 |
| --- | --- | --- | --- | --- | --- | --- | --- | --- | --- | --- | --- | --- | --- | --- | --- | --- |
| S1 | 0.3310 | 0.3227 | 0.3086 | 0.2996 | 0.3012 | 0.2706 | 0.2624 | 0.2737 | 0.2802 | 0.2161 | 0.2375 | 0.2782 | 0.2932 | 0.2973 | 0.2886 | 0.2810 |
| S48 | 0.3308 | 0.3224 | 0.3084 | 0.2994 | 0.3010 | 0.2704 | 0.2622 | 0.2735 | 0.2800 | 0.2159 | 0.2373 | 0.2780 | 0.2930 | 0.2971 | 0.2884 | 0.2808 |
| S21 | 0.3308 | 0.3225 | 0.3084 | 0.2994 | 0.3010 | 0.2704 | 0.2622 | 0.2735 | 0.2800 | 0.2159 | 0.2373 | 0.2780 | 0.2930 | 0.2971 | 0.2884 | 0.2808 |
| S73 | 0.3317 | 0.3233 | 0.3093 | 0.3003 | 0.3018 | 0.2713 | 0.2630 | 0.2743 | 0.2809 | 0.2168 | 0.2382 | 0.2788 | 0.2938 | 0.2980 | 0.2893 | 0.2817 |
| S97 | 0.3281 | 0.3198 | 0.3057 | 0.2967 | 0.2983 | 0.2677 | 0.2595 | 0.2708 | 0.2773 | 0.2132 | 0.2346 | 0.2753 | 0.2903 | 0.2944 | 0.2858 | 0.2782 |
| S40 | 0.3674 | 0.3591 | 0.3450 | 0.3360 | 0.3376 | 0.3070 | 0.2988 | 0.3101 | 0.3166 | 0.2525 | 0.2739 | 0.3146 | 0.3296 | 0.3337 | 0.3251 | 0.3175 |
| S41 | 0.3739 | 0.3656 | 0.3516 | 0.3425 | 0.3441 | 0.3136 | 0.3053 | 0.3166 | 0.3232 | 0.2590 | 0.2805 | 0.3211 | 0.3361 | 0.3402 | 0.3316 | 0.3240 |
| S87 | 0.3688 | 0.3604 | 0.3464 | 0.3374 | 0.3389 | 0.3084 | 0.3001 | 0.3115 | 0.3180 | 0.2539 | 0.2753 | 0.3160 | 0.3309 | 0.3351 | 0.3264 | 0.3188 |
| S10 | 0.4271 | 0.4188 | 0.4047 | 0.3957 | 0.3973 | 0.3667 | 0.3585 | 0.3698 | 0.3763 | 0.3122 | 0.3336 | 0.3743 | 0.3893 | 0.3934 | 0.3847 | 0.3771 |
| S51 | 0.4347 | 0.4263 | 0.4123 | 0.4033 | 0.4049 | 0.3743 | 0.3660 | 0.3774 | 0.3839 | 0.3198 | 0.3412 | 0.3819 | 0.3969 | 0.4010 | 0.3923 | 0.3847 |
| S79 | 0.4211 | 0.4128 | 0.3988 | 0.3897 | 0.3913 | 0.3608 | 0.3525 | 0.3638 | 0.3704 | 0.3062 | 0.3277 | 0.3683 | 0.3833 | 0.3874 | 0.3788 | 0.3712 |
| S23 | 0.4278 | 0.4194 | 0.4054 | 0.3964 | 0.3980 | 0.3674 | 0.3592 | 0.3705 | 0.3770 | 0.3129 | 0.3343 | 0.3750 | 0.3900 | 0.3941 | 0.3854 | 0.3778 |
| S45 | 0.4288 | 0.4204 | 0.4064 | 0.3974 | 0.3990 | 0.3684 | 0.3602 | 0.3715 | 0.3780 | 0.3139 | 0.3353 | 0.3760 | 0.3910 | 0.3951 | 0.3864 | 0.3788 |
| S46 | 0.4238 | 0.4155 | 0.4015 | 0.3924 | 0.3940 | 0.3635 | 0.3552 | 0.3665 | 0.3731 | 0.3089 | 0.3304 | 0.3710 | 0.3860 | 0.3901 | 0.3815 | 0.3739 |
| S24 | 0.4226 | 0.4143 | 0.4002 | 0.3912 | 0.3928 | 0.3622 | 0.3540 | 0.3653 | 0.3718 | 0.3077 | 0.3291 | 0.3698 | 0.3848 | 0.3889 | 0.3803 | 0.3726 |
| S32 | 0.4254 | 0.4170 | 0.4030 | 0.3940 | 0.3955 | 0.3650 | 0.3567 | 0.3681 | 0.3746 | 0.3105 | 0.3319 | 0.3726 | 0.3875 | 0.3917 | 0.3830 | 0.3754 |
| S12 | 0.3927 | 0.3844 | 0.3704 | 0.3613 | 0.3629 | 0.3323 | 0.3241 | 0.3354 | 0.3420 | 0.2778 | 0.2992 | 0.3399 | 0.3549 | 0.3590 | 0.3504 | 0.3428 |
| S22 | 0.4099 | 0.4016 | 0.3876 | 0.3785 | 0.3801 | 0.3495 | 0.3413 | 0.3526 | 0.3592 | 0.2950 | 0.3164 | 0.3571 | 0.3721 | 0.3762 | 0.3676 | 0.3600 |
| S44 | 0.4086 | 0.4003 | 0.3862 | 0.3772 | 0.3788 | 0.3482 | 0.3400 | 0.3513 | 0.3578 | 0.2937 | 0.3151 | 0.3558 | 0.3708 | 0.3749 | 0.3662 | 0.3586 |
| S47 | 0.3996 | 0.3913 | 0.3772 | 0.3682 | 0.3698 | 0.3392 | 0.3310 | 0.3423 | 0.3488 | 0.2847 | 0.3061 | 0.3468 | 0.3618 | 0.3659 | 0.3573 | 0.3496 |
| S56 | 0.3992 | 0.3909 | 0.3769 | 0.3678 | 0.3694 | 0.3389 | 0.3306 | 0.3419 | 0.3485 | 0.2843 | 0.3057 | 0.3464 | 0.3614 | 0.3655 | 0.3569 | 0.3493 |
| S53 | 0.3993 | 0.3910 | 0.3770 | 0.3680 | 0.3695 | 0.3390 | 0.3307 | 0.3420 | 0.3486 | 0.2845 | 0.3059 | 0.3465 | 0.3615 | 0.3657 | 0.3570 | 0.3494 |
| S60 | 0.3991 | 0.3908 | 0.3767 | 0.3677 | 0.3693 | 0.3387 | 0.3305 | 0.3418 | 0.3483 | 0.2842 | 0.3056 | 0.3463 | 0.3613 | 0.3654 | 0.3568 | 0.3491 |
| S54 | 0.4015 | 0.3932 | 0.3792 | 0.3701 | 0.3717 | 0.3411 | 0.3329 | 0.3442 | 0.3508 | 0.2866 | 0.3080 | 0.3487 | 0.3637 | 0.3678 | 0.3592 | 0.3516 |
| S80 | 0.4007 | 0.3924 | 0.3784 | 0.3693 | 0.3709 | 0.3404 | 0.3321 | 0.3434 | 0.3500 | 0.2858 | 0.3073 | 0.3479 | 0.3629 | 0.3670 | 0.3584 | 0.3508 |
| S9 | 0.3889 | 0.3805 | 0.3665 | 0.3575 | 0.3590 | 0.3285 | 0.3202 | 0.3316 | 0.3381 | 0.2740 | 0.2954 | 0.3360 | 0.3510 | 0.3552 | 0.3465 | 0.3389 |
| S86 | 0.4117 | 0.4034 | 0.3893 | 0.3803 | 0.3819 | 0.3513 | 0.3431 | 0.3544 | 0.3609 | 0.2968 | 0.3182 | 0.3589 | 0.3739 | 0.3780 | 0.3694 | 0.3617 |
| S34 | 0.3972 | 0.3889 | 0.3749 | 0.3658 | 0.3674 | 0.3369 | 0.3286 | 0.3399 | 0.3465 | 0.2823 | 0.3038 | 0.3444 | 0.3594 | 0.3635 | 0.3549 | 0.3473 |
| S84 | 0.3998 | 0.3914 | 0.3774 | 0.3684 | 0.3700 | 0.3394 | 0.3312 | 0.3425 | 0.3490 | 0.2849 | 0.3063 | 0.3470 | 0.3620 | 0.3661 | 0.3574 | 0.3498 |
| S55 | 0.4007 | 0.3924 | 0.3784 | 0.3693 | 0.3709 | 0.3404 | 0.3321 | 0.3434 | 0.3500 | 0.2859 | 0.3073 | 0.3479 | 0.3629 | 0.3671 | 0.3584 | 0.3508 |
| S52 | 0.3879 | 0.3796 | 0.3656 | 0.3565 | 0.3581 | 0.3276 | 0.3193 | 0.3306 | 0.3372 | 0.2730 | 0.2944 | 0.3351 | 0.3501 | 0.3542 | 0.3456 | 0.3380 |
| S38 | 0.2718 | 0.2635 | 0.2495 | 0.2404 | 0.2420 | 0.2115 | 0.2032 | 0.2145 | 0.2211 | 0.1569 | 0.1784 | 0.2190 | 0.2340 | 0.2381 | 0.2295 | 0.2219 |
| S101 | 0.3546 | 0.3462 | 0.3322 | 0.3232 | 0.3248 | 0.2942 | 0.2860 | 0.2973 | 0.3038 | 0.2397 | 0.2611 | 0.3018 | 0.3168 | 0.3209 | 0.3122 | 0.3046 |
| S102 | 0.3561 | 0.3478 | 0.3338 | 0.3247 | 0.3263 | 0.2957 | 0.2875 | 0.2988 | 0.3053 | 0.2412 | 0.2626 | 0.3033 | 0.3183 | 0.3224 | 0.3138 | 0.3062 |
| S85 | 0.3566 | 0.3483 | 0.3342 | 0.3252 | 0.3268 | 0.2962 | 0.2880 | 0.2993 | 0.3058 | 0.2417 | 0.2631 | 0.3038 | 0.3188 | 0.3229 | 0.3143 | 0.3066 |
| S63 | 0.3677 | 0.3594 | 0.3453 | 0.3363 | 0.3379 | 0.3073 | 0.2991 | 0.3104 | 0.3169 | 0.2528 | 0.2742 | 0.3149 | 0.3299 | 0.3340 | 0.3254 | 0.3177 |
| S72 | 0.3660 | 0.3577 | 0.3436 | 0.3346 | 0.3362 | 0.3056 | 0.2974 | 0.3087 | 0.3152 | 0.2511 | 0.2725 | 0.3132 | 0.3282 | 0.3323 | 0.3236 | 0.3160 |
| S71 | 0.3603 | 0.3520 | 0.3379 | 0.3289 | 0.3305 | 0.2999 | 0.2917 | 0.3030 | 0.3095 | 0.2454 | 0.2668 | 0.3075 | 0.3225 | 0.3266 | 0.3180 | 0.3103 |
| S2 | 0.3324 | 0.3241 | 0.3101 | 0.3010 | 0.3026 | 0.2720 | 0.2638 | 0.2751 | 0.2817 | 0.2175 | 0.2389 | 0.2796 | 0.2946 | 0.2987 | 0.2901 | 0.2825 |
| S3 | 0.3228 | 0.3144 | 0.3004 | 0.2914 | 0.2929 | 0.2624 | 0.2541 | 0.2654 | 0.2720 | 0.2079 | 0.2293 | 0.2699 | 0.2849 | 0.2891 | 0.2804 | 0.2728 |
| S26 | 0.3098 | 0.3015 | 0.2875 | 0.2784 | 0.2800 | 0.2495 | 0.2412 | 0.2525 | 0.2591 | 0.1949 | 0.2164 | 0.2570 | 0.2720 | 0.2761 | 0.2675 | 0.2599 |
| S88 | 0.2761 | 0.2678 | 0.2537 | 0.2447 | 0.2463 | 0.2157 | 0.2075 | 0.2188 | 0.2253 | 0.1612 | 0.1826 | 0.2233 | 0.2383 | 0.2424 | 0.2337 | 0.2261 |
| S104 | 0.2925 | 0.2842 | 0.2702 | 0.2611 | 0.2627 | 0.2321 | 0.2239 | 0.2352 | 0.2418 | 0.1776 | 0.1990 | 0.2397 | 0.2547 | 0.2588 | 0.2502 | 0.2426 |
| S4 | 0.3115 | 0.3032 | 0.2892 | 0.2801 | 0.2817 | 0.2512 | 0.2429 | 0.2542 | 0.2608 | 0.1966 | 0.2181 | 0.2587 | 0.2737 | 0.2778 | 0.2692 | 0.2616 |
| S6 | 0.3113 | 0.3029 | 0.2889 | 0.2799 | 0.2814 | 0.2509 | 0.2426 | 0.2540 | 0.2605 | 0.1964 | 0.2178 | 0.2585 | 0.2734 | 0.2776 | 0.2689 | 0.2613 |
| S103 | 0.3563 | 0.3479 | 0.3339 | 0.3249 | 0.3264 | 0.2959 | 0.2876 | 0.2989 | 0.3055 | 0.2414 | 0.2628 | 0.3034 | 0.3184 | 0.3226 | 0.3139 | 0.3063 |
| S7 | 0.3955 | 0.3872 | 0.3731 | 0.3641 | 0.3657 | 0.3351 | 0.3269 | 0.3382 | 0.3447 | 0.2806 | 0.3020 | 0.3427 | 0.3577 | 0.3618 | 0.3532 | 0.3455 |
| S8 | 0.3972 | 0.3889 | 0.3749 | 0.3658 | 0.3674 | 0.3369 | 0.3286 | 0.3399 | 0.3465 | 0.2824 | 0.3038 | 0.3444 | 0.3594 | 0.3636 | 0.3549 | 0.3473 |
| S76 | 0.4004 | 0.3921 | 0.3780 | 0.3690 | 0.3706 | 0.3400 | 0.3318 | 0.3431 | 0.3496 | 0.2855 | 0.3069 | 0.3476 | 0.3626 | 0.3667 | 0.3580 | 0.3504 |
| S78 | 0.3960 | 0.3876 | 0.3736 | 0.3646 | 0.3662 | 0.3356 | 0.3274 | 0.3387 | 0.3452 | 0.2811 | 0.3025 | 0.3432 | 0.3582 | 0.3623 | 0.3536 | 0.3460 |
| S77 | 0.3777 | 0.3694 | 0.3554 | 0.3463 | 0.3479 | 0.3174 | 0.3091 | 0.3204 | 0.3270 | 0.2628 | 0.2842 | 0.3249 | 0.3399 | 0.3440 | 0.3354 | 0.3278 |
| S105 | 0.3603 | 0.3520 | 0.3380 | 0.3289 | 0.3305 | 0.2999 | 0.2917 | 0.3030 | 0.3096 | 0.2454 | 0.2668 | 0.3075 | 0.3225 | 0.3266 | 0.3180 | 0.3104 |
| S15 | 0.3662 | 0.3578 | 0.3438 | 0.3348 | 0.3364 | 0.3058 | 0.2976 | 0.3089 | 0.3154 | 0.2513 | 0.2727 | 0.3134 | 0.3284 | 0.3325 | 0.3238 | 0.3162 |
| S19 | 0.3751 | 0.3668 | 0.3528 | 0.3437 | 0.3453 | 0.3148 | 0.3065 | 0.3178 | 0.3244 | 0.2602 | 0.2817 | 0.3223 | 0.3373 | 0.3415 | 0.3328 | 0.3252 |
| S20 | 0.3758 | 0.3674 | 0.3534 | 0.3444 | 0.3460 | 0.3154 | 0.3072 | 0.3185 | 0.3250 | 0.2609 | 0.2823 | 0.3230 | 0.3380 | 0.3421 | 0.3334 | 0.3258 |
| S29 | 0.3876 | 0.3792 | 0.3652 | 0.3562 | 0.3577 | 0.3272 | 0.3189 | 0.3302 | 0.3368 | 0.2727 | 0.2941 | 0.3347 | 0.3497 | 0.3539 | 0.3452 | 0.3376 |
| S31 | 0.3781 | 0.3698 | 0.3558 | 0.3467 | 0.3483 | 0.3177 | 0.3095 | 0.3208 | 0.3274 | 0.2632 | 0.2846 | 0.3253 | 0.3403 | 0.3444 | 0.3358 | 0.3282 |
| S33 | 0.3724 | 0.3641 | 0.3500 | 0.3410 | 0.3426 | 0.3120 | 0.3038 | 0.3151 | 0.3216 | 0.2575 | 0.2789 | 0.3196 | 0.3346 | 0.3387 | 0.3300 | 0.3224 |
| S35 | 0.3604 | 0.3521 | 0.3380 | 0.3290 | 0.3306 | 0.3000 | 0.2918 | 0.3031 | 0.3096 | 0.2455 | 0.2669 | 0.3076 | 0.3226 | 0.3267 | 0.3180 | 0.3104 |
| S16 | 0.3190 | 0.3107 | 0.2967 | 0.2876 | 0.2892 | 0.2587 | 0.2504 | 0.2617 | 0.2683 | 0.2041 | 0.2255 | 0.2662 | 0.2812 | 0.2853 | 0.2767 | 0.2691 |
| S17 | 0.3198 | 0.3115 | 0.2974 | 0.2884 | 0.2900 | 0.2594 | 0.2512 | 0.2625 | 0.2690 | 0.2049 | 0.2263 | 0.2670 | 0.2820 | 0.2861 | 0.2775 | 0.2698 |
| S18 | 0.3143 | 0.3060 | 0.2919 | 0.2829 | 0.2845 | 0.2539 | 0.2457 | 0.2570 | 0.2635 | 0.1994 | 0.2208 | 0.2615 | 0.2765 | 0.2806 | 0.2719 | 0.2643 |
| S30 | 0.2601 | 0.2517 | 0.2377 | 0.2287 | 0.2303 | 0.1997 | 0.1914 | 0.2028 | 0.2093 | 0.1452 | 0.1666 | 0.2073 | 0.2223 | 0.2264 | 0.2177 | 0.2101 |
| S37 | 0.3336 | 0.3253 | 0.3112 | 0.3022 | 0.3038 | 0.2732 | 0.2650 | 0.2763 | 0.2828 | 0.2187 | 0.2401 | 0.2808 | 0.2958 | 0.2999 | 0.2913 | 0.2836 |
| S49 | 0.3317 | 0.3234 | 0.3094 | 0.3003 | 0.3019 | 0.2713 | 0.2631 | 0.2744 | 0.2810 | 0.2168 | 0.2382 | 0.2789 | 0.2939 | 0.2980 | 0.2894 | 0.2818 |
| S50 | 0.3428 | 0.3344 | 0.3204 | 0.3114 | 0.3130 | 0.2824 | 0.2742 | 0.2855 | 0.2920 | 0.2279 | 0.2493 | 0.2900 | 0.3050 | 0.3091 | 0.3004 | 0.2928 |
| S39 | 0.3416 | 0.3333 | 0.3193 | 0.3102 | 0.3118 | 0.2813 | 0.2730 | 0.2843 | 0.2909 | 0.2267 | 0.2482 | 0.2888 | 0.3038 | 0.3079 | 0.2993 | 0.2917 |
| S96 | 0.3563 | 0.3479 | 0.3339 | 0.3249 | 0.3265 | 0.2959 | 0.2877 | 0.2990 | 0.3055 | 0.2414 | 0.2628 | 0.3035 | 0.3185 | 0.3226 | 0.3139 | 0.3063 |
| S98 | 0.3615 | 0.3532 | 0.3391 | 0.3301 | 0.3317 | 0.3011 | 0.2929 | 0.3042 | 0.3107 | 0.2466 | 0.2680 | 0.3087 | 0.3237 | 0.3278 | 0.3192 | 0.3115 |
| S99 | 0.3643 | 0.3559 | 0.3419 | 0.3329 | 0.3344 | 0.3039 | 0.2956 | 0.3070 | 0.3135 | 0.2494 | 0.2708 | 0.3115 | 0.3264 | 0.3306 | 0.3219 | 0.3143 |
| S5 | 0.3297 | 0.3214 | 0.3074 | 0.2983 | 0.2999 | 0.2694 | 0.2611 | 0.2724 | 0.2790 | 0.2148 | 0.2363 | 0.2769 | 0.2919 | 0.2960 | 0.2874 | 0.2798 |
| S14 | 0.2547 | 0.2464 | 0.2323 | 0.2233 | 0.2249 | 0.1943 | 0.1861 | 0.1974 | 0.2039 | 0.1398 | 0.1612 | 0.2019 | 0.2169 | 0.2210 | 0.2124 | 0.2047 |
| S61 | 0.2654 | 0.2571 | 0.2430 | 0.2340 | 0.2356 | 0.2050 | 0.1968 | 0.2081 | 0.2146 | 0.1505 | 0.1719 | 0.2126 | 0.2276 | 0.2317 | 0.2230 | 0.2154 |
| S62 | 0.2678 | 0.2595 | 0.2455 | 0.2364 | 0.2380 | 0.2075 | 0.1992 | 0.2105 | 0.2171 | 0.1529 | 0.1744 | 0.2150 | 0.2300 | 0.2342 | 0.2255 | 0.2179 |
| S11 | 0.0286 | 0.0309 | 0.0778 | 0.1842 | 0.1858 | 0.1904 | 0.1822 | 0.1935 | 0.2001 | 0.1935 | 0.2149 | 0.2917 | 0.3067 | 0.3109 | 0.3022 | 0.2946 |
| S13 | 0.0212 | 0.0365 | 0.0834 | 0.1898 | 0.1914 | 0.1960 | 0.1878 | 0.1991 | 0.2057 | 0.1991 | 0.2205 | 0.2973 | 0.3123 | 0.3165 | 0.3078 | 0.3002 |
| S65 | 0.0000 | 0.0310 | 0.0779 | 0.1843 | 0.1859 | 0.1905 | 0.1823 | 0.1936 | 0.2001 | 0.1935 | 0.2150 | 0.2918 | 0.3068 | 0.3110 | 0.3023 | 0.2947 |
| S42 | 0.0310 | 0.0000 | 0.0695 | 0.1760 | 0.1776 | 0.1822 | 0.1740 | 0.1853 | 0.1918 | 0.1852 | 0.2066 | 0.2835 | 0.2985 | 0.3026 | 0.2940 | 0.2864 |
| S57 | 0.0779 | 0.0695 | 0.0000 | 0.1620 | 0.1635 | 0.1682 | 0.1599 | 0.1712 | 0.1778 | 0.1712 | 0.1926 | 0.2695 | 0.2845 | 0.2886 | 0.2800 | 0.2723 |
| S36 | 0.1843 | 0.1760 | 0.1620 | 0.0000 | 0.0139 | 0.1591 | 0.1509 | 0.1622 | 0.1688 | 0.1622 | 0.1836 | 0.2604 | 0.2754 | 0.2796 | 0.2709 | 0.2633 |
| S89 | 0.1859 | 0.1776 | 0.1635 | 0.0139 | 0.0000 | 0.1607 | 0.1525 | 0.1638 | 0.1703 | 0.1637 | 0.1851 | 0.2620 | 0.2770 | 0.2811 | 0.2725 | 0.2649 |
| S58 | 0.1905 | 0.1822 | 0.1682 | 0.1591 | 0.1607 | 0.0000 | 0.0461 | 0.0574 | 0.0640 | 0.1332 | 0.1546 | 0.2315 | 0.2465 | 0.2506 | 0.2419 | 0.2343 |
| S59 | 0.1823 | 0.1740 | 0.1599 | 0.1509 | 0.1525 | 0.0461 | 0.0000 | 0.0318 | 0.0384 | 0.1249 | 0.1463 | 0.2232 | 0.2382 | 0.2423 | 0.2337 | 0.2261 |
| S74 | 0.1936 | 0.1853 | 0.1712 | 0.1622 | 0.1638 | 0.0574 | 0.0318 | 0.0000 | 0.0242 | 0.1362 | 0.1577 | 0.2345 | 0.2495 | 0.2537 | 0.2450 | 0.2374 |
| S75 | 0.2001 | 0.1918 | 0.1778 | 0.1688 | 0.1703 | 0.0640 | 0.0384 | 0.0242 | 0.0000 | 0.1428 | 0.1642 | 0.2411 | 0.2561 | 0.2602 | 0.2516 | 0.2439 |
| S27 | 0.1935 | 0.1852 | 0.1712 | 0.1622 | 0.1637 | 0.1332 | 0.1249 | 0.1362 | 0.1428 | 0.0000 | 0.0519 | 0.1770 | 0.1919 | 0.1961 | 0.1874 | 0.1798 |
| S28 | 0.2150 | 0.2066 | 0.1926 | 0.1836 | 0.1851 | 0.1546 | 0.1463 | 0.1577 | 0.1642 | 0.0519 | 0.0000 | 0.1984 | 0.2134 | 0.2175 | 0.2088 | 0.2012 |
| S90 | 0.2918 | 0.2835 | 0.2695 | 0.2604 | 0.2620 | 0.2315 | 0.2232 | 0.2345 | 0.2411 | 0.1770 | 0.1984 | 0.0000 | 0.1158 | 0.1199 | 0.1112 | 0.1036 |
| S91 | 0.3068 | 0.2985 | 0.2845 | 0.2754 | 0.2770 | 0.2465 | 0.2382 | 0.2495 | 0.2561 | 0.1919 | 0.2134 | 0.1158 | 0.0000 | 0.0577 | 0.0880 | 0.1002 |
| S95 | 0.3110 | 0.3026 | 0.2886 | 0.2796 | 0.2811 | 0.2506 | 0.2423 | 0.2537 | 0.2602 | 0.1961 | 0.2175 | 0.1199 | 0.0577 | 0.0000 | 0.0921 | 0.1043 |
| S93 | 0.3023 | 0.2940 | 0.2800 | 0.2709 | 0.2725 | 0.2419 | 0.2337 | 0.2450 | 0.2516 | 0.1874 | 0.2088 | 0.1112 | 0.0880 | 0.0921 | 0.0000 | 0.0956 |
| S92 | 0.2947 | 0.2864 | 0.2723 | 0.2633 | 0.2649 | 0.2343 | 0.2261 | 0.2374 | 0.2439 | 0.1798 | 0.2012 | 0.1036 | 0.1002 | 0.1043 | 0.0956 | 0.0000 |
